# Co‐Extinctions and Co‐Compensatory Species Responses to Climate Change Moderate Ecosystem Futures

**DOI:** 10.1111/gcb.70539

**Published:** 2025-10-08

**Authors:** Thomas J. Williams, Clement R. Garcia, Jasmin A. Godbold, Philippe Archambault, Martin Solan

**Affiliations:** ^1^ School of Ocean and Earth Science, National Oceanography Centre Southampton University of Southampton Southampton UK; ^2^ Centre for Environment Fisheries and Aquaculture Science (Cefas) Suffolk UK; ^3^ Takuvik, ArcticNet, Québec Océan, Département de Biologie Université Laval Québec Canada

**Keywords:** cascade extinction, co‐extinction, extinction risk, secondary extinction, trait covariance

## Abstract

Consensus has been reached that the sequential loss of biodiversity leads to a non‐linear and accelerating decline in ecosystem properties. The form of this relationship, however, is based on theory and empirically derived observations that do not include species co‐extinctions. Here, we use data from marine benthic invertebrate communities to parameterise trait‐based extinction models that adjust the probability of species extirpation and compensation by including the dependencies between different species across a gradient of climate‐driven environmental change. Our simulations reveal that the inclusion of static co‐extinctions leads to more pronounced declines in the trajectories of sediment bioturbation—a process of great importance to the functioning of marine ecosystems—than those observed with sequential losses of single species. Compensatory mechanisms and the allowance of the formation of new interactions derived from local and regional species pools moderate the compounding influence of co‐extinction but introduce additional variability in community response depending on the composition and functional role of incoming and outgoing species. Our observations emphasise the importance of accounting for local and regional community dynamics, especially in highly connected systems that are prone to extinction cascades when projecting the ecosystem consequences of altered biodiversity.

## Graphical abstract

Predictions of what happens to an ecosystem following the loss of species often assume species disappear one by one, but some drivers of extinction target multiple species at a time that share certain attributes. Our study shows that when such co‐extinctions occur, the consequences of biodiversity loss can differ sharply from previous expectations. Species also vary in how they respond to co‐extinction, further shaping ecological outcomes. Only by accounting for these patterns can we better predict how human activity and climate change will alter ecosystems.

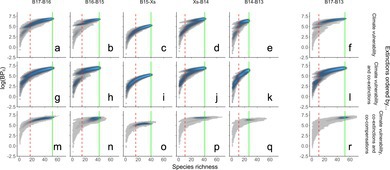

## 1 Introduction

Localised species loss is generally expected to reduce ecosystem functioning (Cardinale et al. 2012). Evidence consistently indicates that the magnitude and trajectory of species loss will reflect the relative vulnerabilities of species to extinction (Payne et al. 2016), the covariance between extinction risk and the functional traits of individual species (Solan et al. 2004) and the influence of post‐extinction community dynamics (McIntyre et al. 2007; Thomsen et al. 2017; Thomsen et al. 2019). Whilst the inclusion of these sources of variation can lead to more relevant and realistic ecological projections (Naeem 2008), they remain insufficient to explain observed patterns in the biodiversity–function relationship. This is because the ecological consequences of biodiversity adjustments are also expressed through longer term changes in species interactions (Hughes 2012), including those related to niche emergence (Cazzolla Gatti et al. 2018), that are disconnected from the initial cause of extinction (Brook et al. 2008). Co‐extinctions of obligate species, likely the most common (Koh et al. 2004) but underappreciated (Stork and Lyal 1993) form of extinction, can arise during and after the initial wave of primary extinctions (Koh et al. 2004; Dunne and Williams 2009; Brodie et al. 2014) and have additional functional consequences (Luza et al. 2024). Yet, the ecosystem implications of these secondary extinctions (Sanders et al. 2015; Valiente‐Banuet et al. 2015; Strona and Bradshaw 2018; Strona and Bradshaw 2022) have not been measured directly (Kehoe et al. 2021; Raine et al. 2018; Blanchard and Munoz 2022), compared to those of singular extinctions (Ives and Cardinale 2004) or been incorporated into projections of the ecosystem consequences of biodiversity loss (Cardinale et al. 2012). Theory suggests that the ecosystem effects of co‐extinctions are a reflection of network connectivity and community structure (Thébault et al. 2007; Dallas and Cornelius 2015; Morton et al. 2022), but conflicting conclusions exist regarding how co‐extinctions affect functional diversity (Vieira et al. 2013; Petchey et al. 2008) and redundancy (Sanders et al. 2018; Biggs et al. 2020), making it challenging to determine the most likely ecosystem consequences of biodiversity loss.

Failure to adopt a full community perspective and consider processes, such as co‐extinction and co‐compensation, means that the generalised biodiversity–function curve is unlikely to generate robust estimates of future ecosystem properties (Thomsen et al. 2017; Naeem 2008; Garcia et al. 2021). Post‐extinction performance of a surviving community will depend not only on the absolute loss of species but on how associated adjustments to network connectivity and structure alter species interactions (Morton et al. 2022) and the likelihood and/or expression of compensatory mechanisms (Thomsen et al. 2017; Thomsen et al. 2019; Gonzalez and Loreau 2009; Pan et al. 2016) across different contexts (Garcia et al. 2021). Species with a low population density, slow life history, high trophic level, and small geographical range size tend to be associated with a high extinction risk (Purvis et al. 2000) and low compensatory capacity because the species' range and niche are out of equilibrium (Sheth and Angert 2018). In contrast, the number and probability of extinction cascades are minimised in regions exhibiting high levels of geographic isolation (Albouy et al. 2019) and maximised when species are most connected (Eklöf and Ebenman 2006; Curtsdotter et al. 2011; Baumgartner et al. 2020). The ecosystem consequences of species loss, compensation, and secondary extinctions, however, do not necessarily reflect modifications to community structure (Thomsen et al. 2019; Brodie et al. 2014). Rather, they reflect the extent to which alterations to functional roles (Fetzer et al. 2015), trait expression (Wohlgemuth et al. 2017; Cassidy et al. 2020; Sanders et al. 2024) and adjusted interactions with the environment (Dolbeth et al. 2019) and other species (Bimler et al. 2018) are presented over time (months to years; Godbold and Solan 2013; Touchton and Smith 2011). Hence, a species with a low (or high) probability of co‐extinction may have a high (or low) potential to compensate through interactions with other surviving species (Vieira and Almeida‐Neto 2015) but, in terms of ecosystem functioning, may deliver no compensation (Davies et al. 2012), partial, complete, or overcompensation (Pan et al. 2016). Should a species survive primary and secondary bouts of extinction, emergent compensatory responses (e.g., competitive release, alterations to resource partitioning, assemblage reorganisation; Thomsen et al. 2017; Thomsen et al. 2019) increase the likelihood of alternative ecosystem outcomes (Thomsen et al. 2017) both within and across a range of spatio‐temporal contexts (Thomsen et al. 2017; Albouy et al. 2019; Cassidy et al. 2020; Wardle and Zackrisson 2005).

Here, we use data from marine benthic invertebrate communities from a region of the Barents Sea currently facing amplified climatic forcing (Lind et al. 2018) to parameterise trait‐based extinction models that adjust the probability of species extirpation and compensation by including the dependencies between different species across an environmental gradient (Solan et al. 2020). In doing so, we explicitly recognise that the sequential loss of species is ordered, first, by extinction risk associated with the transition to increasingly boreal environmental conditions (Wang et al. 2020) and second, by the likelihood that the modified diversity and structure of the community associated with primary extinctions will lead to interaction‐driven co‐extinctions followed by numeric compensation from multiple taxa. We also assume, should compensation from the local species pool not be realised, that immigration of boreal‐adapted species from the regional pool will introduce new interactions that revise extinction probabilities and modify local community dynamics (Albouy et al. 2019; Grebmeier 2012; Kortsch et al. 2015) and associated ecosystem properties (Csapó et al. 2021; Ingvaldsen et al. 2021). In line with expectation, we find that including co‐extinctions in our simulations hastens the decline in sediment bioturbation—a process crucial to the functioning of marine communities—regardless of extinction scenario. However, we show that the influence of co‐extinction depends on location‐specific interdependencies (Garcia et al. 2021; Albouy et al. 2019) between species interactions (Bimler et al. 2018), their vulnerability to change (McLean et al. 2019), and the degree of functional redundancy in the community (Thomsen et al. 2017; Naeem and Wright 2003). We had anticipated that the influence of co‐extinction on bioturbation would be maximised at the polar front, an area where boreal and polar communities converge, but the form of the biodiversity–function relationship varies along the length of the environmental gradient, reflecting differences in species turnover and community structure. Our models also reveal that the greatest declines in sediment bioturbation occur at low levels of environmental forcing, despite elevated numbers of compensating species, presumably because species are close to, or at, the limit of their range distribution (Boakes et al. 2018). These findings emphasise the importance of including the full suite of species responses to perturbations when attempting to project the most likely ecosystem consequences of environmental forcing.

## 2 Methods

### 2.1 Study Location and Environmental Gradient

We use macroinvertebrate data (Data Records S1, Solan et al. 2020) collected as part of a benthic survey of 6 stations (B17, B16, B15, Xs, B14, B13), each sampled four times using 0.1 m^2^ USNL (United States Naval Laboratory) box cores in the northwestern part of the Barents Sea shelf (Figure S1), to parameterise models that predict how alterations to biodiversity associated with climate‐driven change in environmental conditions affect seabed function. To minimise the effect of non‐climatic drivers of change, stations were selected with comparable water depths (228–360 m), sediment type, and bottom fishing activity along the 30° E meridian. The transect of stations—from B13 in the south to B17 in the north, and with station Xs located between B14 and B15 at the location of the average southernmost extent of sea ice (Figure S1)—intersects an established polar front (Jørgensen et al. 2015; Loeng 1991) and exhibits a clear north–south separation in faunal assemblage structure (Solan et al. 2020). Although the precise physical location of the front is contested (Oziel et al. 2016; Oziel et al. 2017), the zone exhibits a relatively stationary behaviour (Onarheim and Teigen 2018) and is becoming more persistent (Barton et al. 2018). We argue that this transect serves as a present‐day gradient of climate‐driven change and use it to parameterise models that predict how associated stepped changes in biodiversity affect seabed function. We investigate differences in the magnitude and extent of forcing by parameterising our models with sequential station‐to‐station species vulnerabilities (based on interstation transitions from: B17‐B16 | B16‐B15 | B15‐Xs | Xs‐B14 | B14‐B13) and compare these simulations to equivalent extinctions based on change across the entire gradient (B17‐B13). Hence, the most northerly (B17) and most southerly (B13) stations in our transect represent the most polar and most boreal community respectively. In stepped scenarios between neighbouring stations, we assume a northward advance of climate change forcing such that the northernmost station represents the pre‐extinction community (closest to pole) and the southernmost station represents the post‐extinction community (closest to boreal).

### 2.2 Probabilistic Modelling

We developed a probabilistic trait‐based model to explore the effects of local extinction scenarios and the associated compensatory response of natural communities (Solan et al. 2004) and to predict how altered diversity associated with climatic‐driven environmental change may affect seabed functioning in the Arctic. We establish the relationships between an index of community‐level bioturbation potential (${\mathrm{BP}}_{c}$, Solan et al. 2004), estimated from *per capita* contributions of sediment‐dwelling invertebrates to sediment reworking (Figure S2) based on root‐transformed mean body‐size (across the entire transect; $B_{i}^{0.5}$, range: 0.008–1.225 g), abundance ($A_{i}$, range: 6–1350 m^⁻^^2^), predefined mobility ($M_{i}$, range: 1–4) and sediment reworking mode ($R_{i}$, range: 1–4):

$${\mathrm{BP}}_{i}=B_{i}^{0.5}\times M_{i}\times R_{i}$$

$${\mathrm{BP}}_{p}={\mathrm{BP}}_{i}\times A_{i}$$

$${\mathrm{BP}}_{c}={\mathrm{\Sigma BP}}_{p}$$

where ${\mathrm{BP}}_{i}$ is the bioturbation potential of an individual, ${\mathrm{BP}}_{p}$ is the bioturbation potential of a population of individuals and ${\mathrm{BP}}_{c}$ is the bioturbation potential of the community (Solan et al. 2004). Following Solan et al. (2004), we use ${\mathrm{BP}}_{c}$ as a means to generate benthic ecosystem processes under novel scenarios. A summary table of species‐specific $B_{i}^{0.5}$, $A_{i}$, $M_{i}$ and $R_{i}$ is provided in Table S1.

As climate‐driven changes across the Arctic will transform benthic communities through the selective removal of vulnerable taxa (Jørgensen et al. 2019), subsequently triggering compensatory responses, co‐extinctions, and increasing dominance of boreal‐adapted taxa (Csapó et al. 2021), we selectively eliminate taxa from the pre‐extinction species pool before calculating the response of the surviving community through compensatory mechanisms established for the regional species pool. As specific tolerances of Arctic invertebrates to climatic drivers are scarce (Degen and Faulwetter 2019), we derive probability‐based orders of species extinction and, reciprocally, their likelihood to compensate from ranked vulnerabilities calculated across each pair of neighbouring stations based on the percentage difference in biomass between the pre‐ and post‐extinction communities for all taxa in the regional species pool (Table S2 Code S1). Hence, a taxon with a high vulnerability score (i.e., highest biomass at the pre‐extinction station and lowest biomass or absence at the post‐extinction station) would have both a high probability of going extinct and a low probability to compensate. In adopting this approach, we explicitly recognise realistic, non‐random changes in biodiversity that emerge as forcing progresses through multiple stages to avoid having to prescribe a single directional species‐specific vulnerability that spans the entirety of the forcing (Bracken et al. 2008).

As taxa are sequentially extirpated and the surviving community numerically compensates to replenish biomass, a revised ${\mathrm{BP}}_{c}$ is calculated and taxa‐specific contributions to ${\mathrm{BP}}_{c}$ are modified when they increase or decrease in abundance. At each iteration we calculate *per capita* contributions (${\mathrm{BP}}_{p}$) for all taxa in the regional community and run our simulations (*n* = 500 per scenario) until all taxa become locally extinct. However, each simulation is only valid to the level of biodiversity typically observed at the respective post‐extinction station. That is, we assume the median species richness of each station reflects the existing local community which, in turn, is regulated within their temporal fluctuations (Gotelli et al. 2017). Similarly, we only allow for species to compensate up to the median abundance observed within the regional cluster of northern versus southern stations (Northern cluster: B17, B16; Southern cluster: B15, Xs, B14, B13; Figure S3; Solan et al. 2020) to prevent any taxa increasing in abundance to improbable levels (Code S2). As any alteration in local communities associated with climate change may be offset by more resilient taxa from a wider area (Ingvaldsen et al. 2021), we allow for taxa present in regional cluster species pools that were not present in the pre‐extinction assemblage to be introduced and compensate (Garcia et al. 2021). This allows for the possibility that taxa from the regional pool can arrive and increase species richness, as would occur in a natural system.

#### 2.2.1 Correlations, Co‐Extinctions and Co‐Compensations

As biotic interactions build up complex ecological networks through which the loss of one species can alter the vulnerability of other species (co‐extinction, Sanders et al. 2015; Valiente‐Banuet et al. 2015; Strona and Bradshaw 2018; Strona and Bradshaw 2022), we estimated interactions between taxa from positive and negative correlations in biomass across all station replicates (*n* = 24) and for each species (abundance > 1, *n* = 69, Figure S4a). Whilst it has been argued that species correlations carry limited information on network interactions (Pinto et al. 2022; Loreau and de Mazancourt 2013), they nevertheless provide a conservative starting point in the absence of such information. Hence, as correlation does not necessarily indicate co‐dependency, we adopted a prudent approach by only selecting correlations that were 1.5 standard deviations outside the mean correlation score (0.0397; Figure S4b Table S3; 466 correlations, Code S3). This reasonably assumes that the strongest correlations are more likely to indicate a genuine biotic interaction, as opposed to spurious and/or environmentally driven taxa co‐occurrences. Within each extinction iteration, we used these positive correlations to calculate the co‐extinction risk of other taxa (multiple taxa can be simultaneously selected, Code S4) or, in the absence of co‐extinction, to reduce their probability of compensating. This approach ensures that only the highest correlations are selected whilst allowing for indirect effects, such as competitive and/or predator release. To account for the greater chance of surviving taxa contributing to compensation following co‐dependent release, we recalculate the probability of compensation within the community using the negative correlations of the extirpated co‐dependent taxa (Code S5). Following local extinction, we assume conditions are no longer supportive (Code S5) to avoid compensation through reintroduction. The model is constrained to secondary extinction and compensatory mechanisms to avoid an uncontrollable cascade from the primary cause of extinction.

We acknowledge that multiple species can contribute to compensation, particularly when lost biomass is not entirely replaced by the initial responding species (Code S6; Figure S5). To improve the realism of our simulations of biodiversity change (Naeem 2008), we limit the amount of compensation of each taxon to the median abundance observed in the regional species pool to allow several compensators to respond to an extirpation. In doing so, we avoid overinflation of the total biomass following compensation whilst allowing biomass to vary with the removal and addition of species. When the median abundance of all taxa is reached during a simulation, biomass is lost from the system and a sequence of uncompensated extinction events is initiated until the next taxon from the regional species pool is introduced into the system. This follows the expectation that climate change will have negative consequences for seafloor biomass (Jones et al. 2014).

### 2.3 Statistical Analyses

To examine the effect of extinctions on ecosystem functioning, we ran Generalised Additive Models (GAMs) with ${\mathrm{BP}}_{c}$ as the response variable given the non‐linear nature of biodiversity–function relationships (Gross and Cardinale 2005). A smooth term of species richness within each extinction scenario (by = scenario) and a smooth term of species richness in isolation were the main explanatory variables of interest. The extinction scenario was also included as a factorial covariate and the model was estimated using the fast restricted maximum likelihood (fREML) method, which is designed for fitting generalised additive models (GAMs) on large datasets (Wood et al. 2015). The interaction was included to investigate whether the effect of extinctions differs with each spatially explicit extinction scenario (Code S7). To deduce the best GAM fit, we conducted a backward stepwise selection on models estimated with the maximum likelihood (ML) method, informed by the Akaike information criteria (AIC), the deviance explained and inspection of model residual patterns using the visreg 2.7.0 and lmtest 0.9–40 packages (Breheny and Burchett 2017; Zeileis and Hothorn 2002; Table S4). To improve result standardisation and comparability, we ran linear models with the same structure of the best GAM estimated with fREML, and visually compared the partial estimated slopes of ${\mathrm{BP}}_{c}$ as a response to species richness using the lm function in base R (Figure S6).

To investigate differences in emergent adjustments to extinction probabilities as species are lost from the community within each extinction scenario, we used a series of linear models to examine changes in the climate vulnerability of all species going extinct (_ALL_), species still present within the community (_PRESENT_), species going extinct as a result of climate vulnerabilities (_CLIMATE_), and species going extinct as a result of species co‐dependencies (_CO‐EXT_) as species richness declined (Table S5). All statistical analyses, data exploration, and plotting were performed using the R statistical and programming environment (R Core Team 2023) and the R packages ‘qgraph’ (visual correlation networks; Epskamp et al. 2012), ‘MetBrewer’ (formatting graphical outputs; Mills 2022), ‘mgcv’ (Generalised Additive Models; Wood et al. 2015; Simon N. Wood 2011; S. N. Wood 2017), ‘parallel’ (cluster computing of GAMs; R Core Team 2023), ‘stats’ (correlation calculations and matrices; R Core Team 2023), and ‘tidyverse’ (data exploration and plotting; Wickham et al. 2019). Code for creating model output figures can be found at the end of the supporting information (Code S8).

## 3 Results

### 3.1 Simulated Ecosystem Futures

**FIGURE 1:**
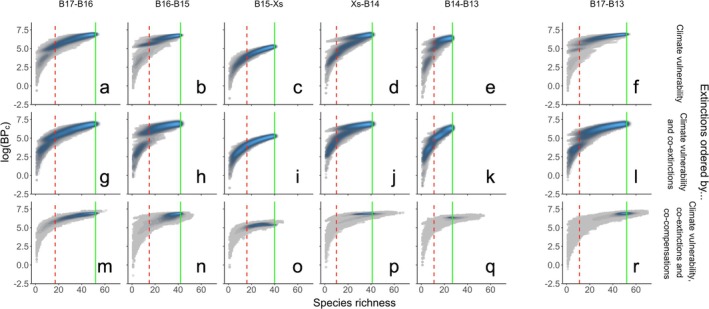
Changes in community bioturbation potential (log(${\mathrm{BP}}_{c}$)) following climate‐driven extinctions (upper panels), combined with interaction‐derived co‐extinctions (middle panels) and post‐extinction compensations (bottom panels) associated with step‐wise environmental transitions from stations (a, g, m) B17‐ B16, (b, h, n) B16 ‐B15 (c, i, o) B15 ‐Xs (d, j, p) Xs‐B14 (e, k, q) B14‐B13 and the transect‐wide transition from stations (f, l, r) B17‐ B13 in the Barents Sea. Colour intensity (grey–blue) reflects an increasing density (low to high) of data points with the pre‐extinction species richness (vertical green solid line) and predicted post‐extinction species richness (vertical red dashed line) represented. Co‐extinctions lead to an increase in colour intensity along the main species‐function trajectory, whilst compensations increase the spread of data points. Simulations, *n* = 500 per panel.

**FIGURE 2:**
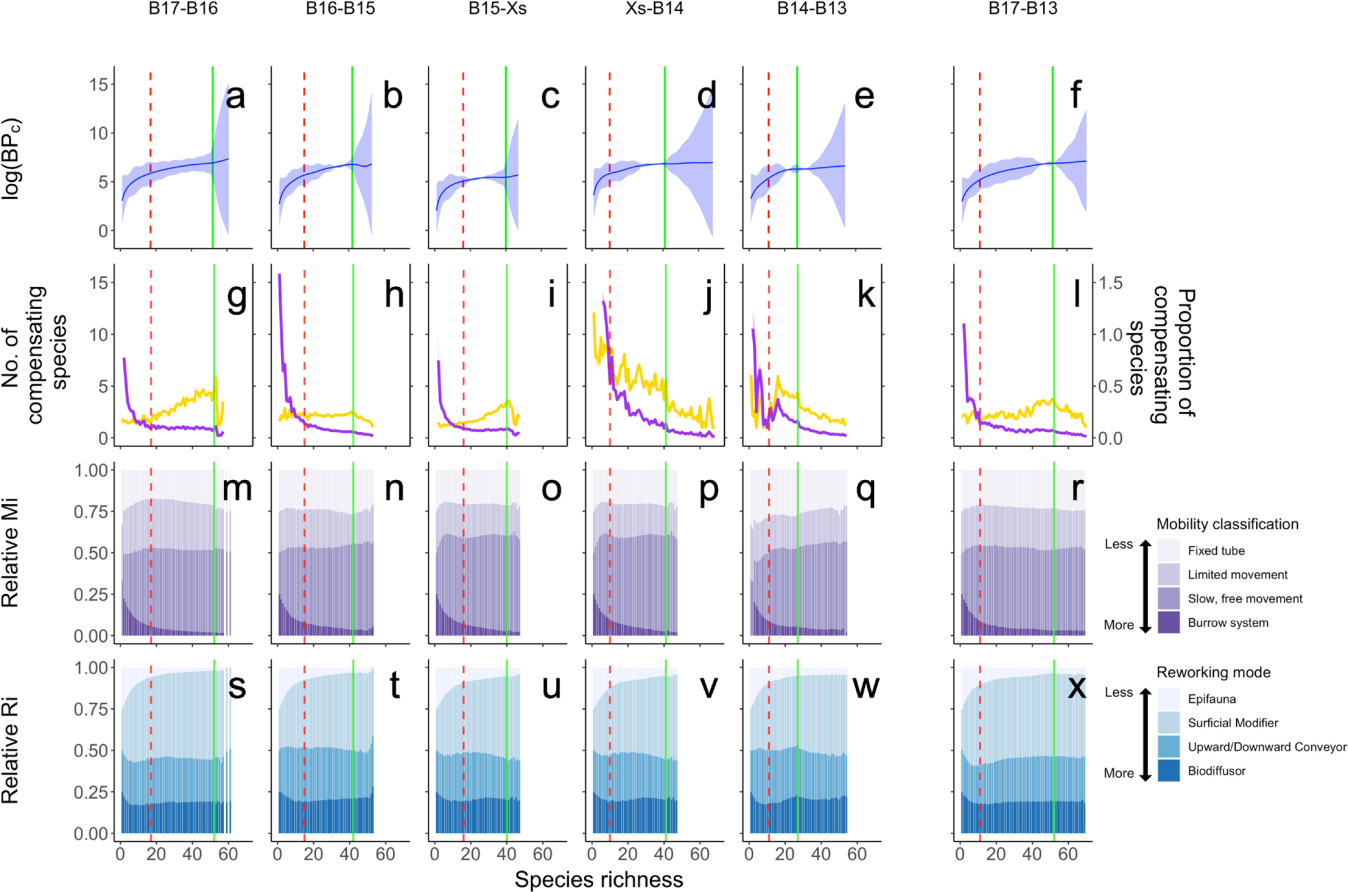
Predicted biodiversity–ecosystem function relation curves (mean ± SE, first row) represented with a generalised additive model (GAM, solid lines) and the standard error (shaded areas). In the second row, post‐extinction compensations (mean ± SE, number, in yellow) and the proportion of species (purple, > 1 when the number of compensating taxa relies on greater influx from the regional species pool) are shown. The reorganisation of functional groups characterised by their mobility (third row) and sediment reworking (fourth row) associated with step‐wise environmental transitions are shown between stations (B17‐B16, panels a, g, m, s; B16‐B15, panels b, h, n, t; B15‐Xs, panels c, i, o, u; Xs‐B14, panels d, j, p, v; B14‐B13, panels e, k, q, w) and the transect‐wide transition from stations B17‐B13 (panels f, l, r, x) in the Barents Sea. The pre‐extinction species richness (vertical green solid line) and predicted (median of observed data) post‐extinction species richness (vertical red dashed line) define the upper and lower boundaries of the most ecologically realistic output.

**FIGURE 3:**
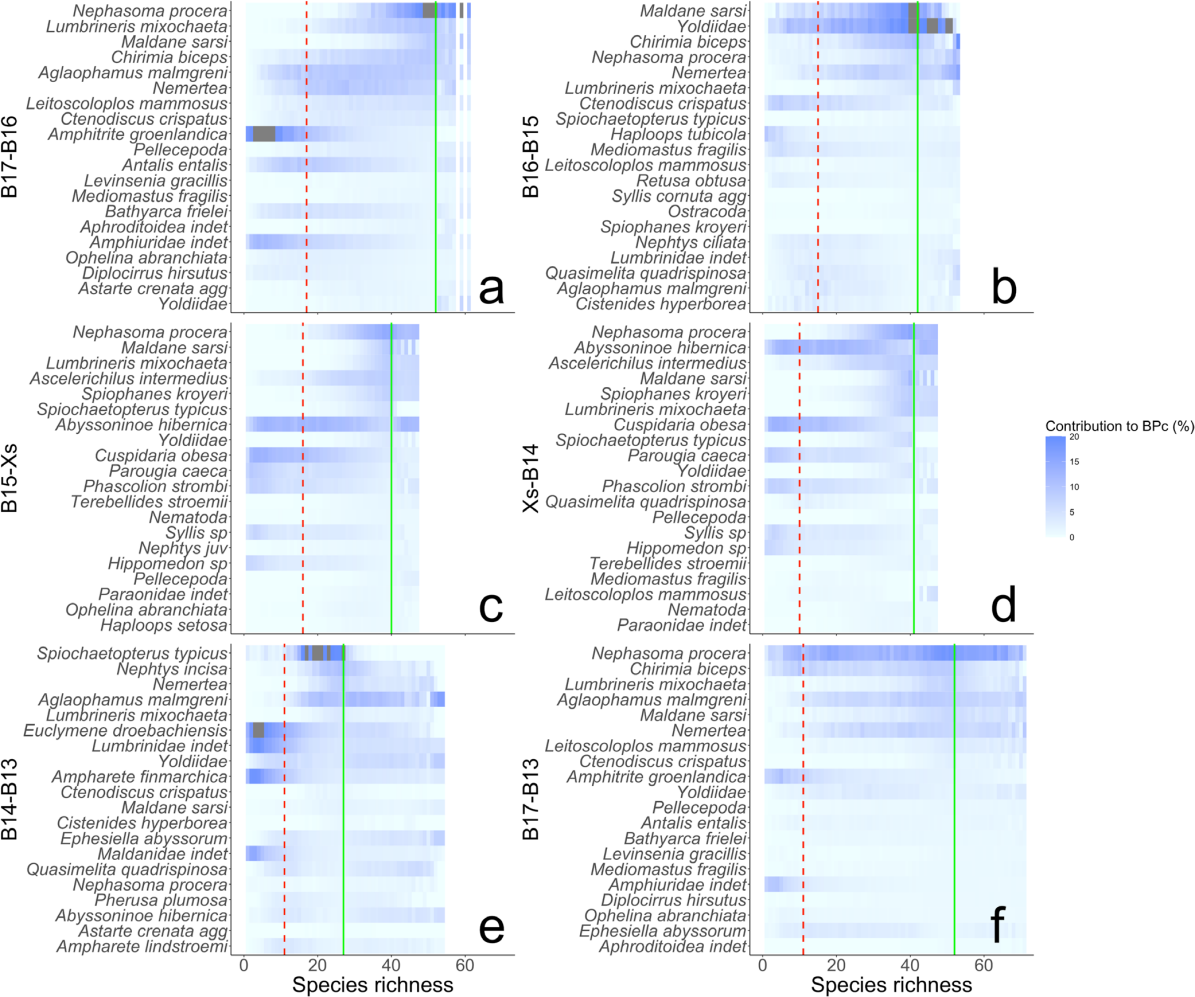
Taxonomic reorganisation during simulated extinction events following step‐wise environmental transitions from station (a) B17‐B16, (b) B16‐B15 (c) B15‐Xs (d) Xs‐B14 (e) B14‐B13 and the transect‐wide transition from stations (f) B17‐B13 in the Barents Sea. Colour shading (white, low⏤dark blue, high) represents the relative contributions of individual taxa to ${\mathrm{BP}}_{c}$ at each sequential level of local extinction. The pre‐extinction species richness (vertical green solid line), predicted post‐extinction species richness (vertical red dashed line) and subset of twenty taxa that contribute most to functioning pre‐extinctions are represented. Contributions above 20% greyed.

In the absence of co‐extinction and compensatory dynamics (Figure 1a–f), we find that the form of the biodiversity‐function curve approximates expectations (accelerating reductions in functioning with declining species richness) with notable climate‐dependent differences in the form of the curve. Our simulations also commonly feature (except B15 to Xs, Figure 1c) step changes within the species‐function trajectory that reflect the loss or gain of species that disproportionately contribute to function. These become more pronounced when extinctions, ordered by climate vulnerability, incorporate co‐extinctions (Figure 1g–l). Co‐compensatory mechanisms, however, temper the functional consequences associated with species loss (Figure 1m–r), even when the proportion and number of compensating species increases with species loss (station Xs to B14, station B14 to B13; Figures 2d and 3e). We also find that the taxa contributing most to community‐level ecosystem functioning (% ${\mathrm{BP}}_{c}$) transition from an Annelid dominated pre‐extinction community (solid green line Figure 3a–e) to a more diverse post‐extinction community (dashed red line Figure 3a–e), and that there is higher functional redundancy across the polar front (station B15 to Xs and Xs to B14; Figure 2c).

Our simulations reveal differences in the form of the biodiversity–function curve for each of our scenarios (Table 1), and we observe non‐linear changes in the rate and magnitude of function as species loss progresses (Table 2) that decrease as species loss extends below the level of biodiversity typically observed at the post‐extinction station (dashed red vertical line, Figure 2). Tube dwelling (Figure 2m–r) and surficial modifying species (Figure 2s–x) are lost first, whilst epifauna, deeper burrowers, and conveyer belt lifestyles are preserved (Figure 2m–x). A transect‐wide transition from B17 to B13 exhibits a shallow biodiversity–function trajectory with minimal differences in uncertainty achieved between the pre‐extinction and most likely post‐extinction levels of species richness (Figure 2f). Note, however, that the distribution of uncertainty across the species richness gradient does vary between each step of the extinction transition (compare panels Figure 2a–f). As compensation establishes, biodiversity levels may rise above pre‐extinction species richness (solid green vertical line, Figure 2), although the extent of such an increase and any associated effect on functioning is scenario‐dependent. Within the most likely post‐extinction levels of species richness window (area between the red and green vertical lines, all figures), both the range of the proportion of compensating species (%), and the level of species richness where compensating species is minimised or maximised, varied between scenarios (Table 3, Figure 2g–l), as did the mean (± SD) number of compensating species (Table 3, Figure 2g–l). Hence, the number, composition, and proportion of compensating species are dependent on local circumstance, with the greatest capacity for compensation occurring in communities south of the polar front (Xs‐B14 and B14‐B13, Table 3, Figure 2g–2l).

**TABLE 1:** Analysis of variance (ANOVA, two‐tailed) parametric coefficients generated from the generalised additive model (GAM) of bioturbation potential loss. Except for one scenario (station B16 to station B15), each local extinction event results in a biodiversity–ecosystem functioning curve that is significantly different from the regional extinction scenario (baseline, station B17 to station B13).

Pairwise comparison	Mean difference	Std. error	t value	Significance
B17‐B13 to B17‐B16	68.172	1.244	54.781	< 0.0001
B17‐B13 to B16‐B15	16.439	17.998	0.913	0.361
B17‐B13 to B15‐Xs	−329.480	5.864	−56.189	< 0.0001
B17‐B13 to Xs‐B14	185.129	0.888	208.460	< 0.0001
B17‐B13 to B14‐B13	−35.871	2.486	−14.427	< 0.0001

**TABLE 2:** Approximate significance of smooth term ‘species richness’ in each extinction scenario used in the generalised additive model (GAM) of bioturbation potential loss. The edf (effective degrees of freedom of smooth terms) represents the complexity of the smoother, with an edf of 1 equivalent to a straight line between x (species richness) and y (*BP_c_*). The Ref.df and F columns are test statistics used in an ANOVA (two‐tailed) to determine overall significance (Sig.) of the smoother (unable to draw a horizontal line through the 95% confidence interval of the GAM).

Extinction scenario	edf	Ref.df	F	Sig.
B17‐B13	8.813	8.987	45,603	< 0.0001
B17‐B16	8.022	8.253	29,309	< 0.0001
B16‐B15	8.486	8.738	27,652	< 0.0001
B15‐Xs	5.981	6.309	1567	< 0.0001
Xs‐B14	8.699	8.932	24,758	< 0.0001
B14‐B13	7.092	7.364	9942	< 0.0001

**TABLE 3:** The range in the proportion of compensating species (%), the absolute number (mean ± SD) of compensating species and the level of species richness where compensating species is minimised or maximised (indicated by SR in subscript) between the most likely post‐extinction levels of species richness window (area between the red and green vertical lines, all figures).

Extinction scenario	Compensating species			
	Proportion of total species (%)		Absolute number (mean ± SD)	
	min_n,SR_	max_n,SR_	min_n,SR_	max_n,SR_
B17‐B13	5_n=395, SR=39_	15_n=201, SR=12_	1.46 ± 0.91_n=169, SR=11_	3.76 ± 3.64_n=3086, SR=51_
B17‐B16	7_n=474, SR=51_	12_n=554, SR=18_	1.52 ± 0.54_n=443, SR=17_	4.67 ± 2.51_n=1816, SR=52_
B16‐B15	5_n=2391, SR=42_	14_n=417, SR=15_	1.78 ± 0.61_n=427, SR=17_	2.54 ± 1.08_n=2159, SR=41_
B15‐Xs	7_n=2060, SR=27_	9_n=1313, SR=16_	1.45 ± 0.44_n=1313, SR=16_	3.50 ± 1.70_n=1580, SR=40_
Xs‐B14	9_n=1608, SR=40_	78_n=293, SR=11_	3.69 ± 2.40_n=1608, SR=40_	8.57 ± 3.81_n=293, SR=11_
B14‐B13	15_n=4348, SR=26_	37_n=444, SR=16_	2.18 ± 0.96_n=79, SR=13_	5.95 ± 3.38_n=444, SR=16_

### 3.2 Co‐Extinction and Climate Vulnerability

**FIGURE 4:**
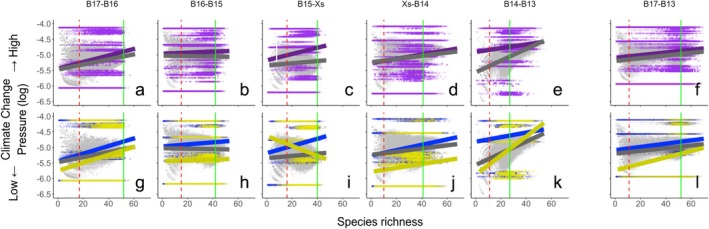
Changes in mean extinction probability (log) of species following step‐wise environmental transitions from station (a‐g) B17‐B16, (b‐h) B16‐B15, (c‐i) B15‐Xs, (d‐j) Xs‐B14, (e‐k) B14‐B13 and the transect‐wide transition from stations (f‐l) B17‐B13 in the Barents Sea. Colours represent the extinction risk for all species (purple), species still present within the community (grey), species going extinct because of climate vulnerabilities (blue) and species going extinct as a result of species co‐dependencies (yellow). The pre‐extinction species richness (vertical green solid line) and post‐extinction species richness (vertical red dashed line) are also shown.

Our simulations show that the incorporation of extinction probabilities related to climate vulnerabilities that lead to primary extinctions, and species co‐dependencies that lead to co‐extinction, moderate ecosystem outcomes based on differences in emergent adjustments to extinction probabilities. We find that, regardless of whether species loss reflects bouts of primary or secondary extinctions, the taxa most vulnerable to climate‐driven change are preferentially removed (purple lines, Figure 4a–f), although the rate of functional loss does vary with scenario. As a result, the extinction probability of the surviving taxa adjusts and tends to be lower than prior to extinction (compare purple to grey lines, Figure 4a–f). Importantly, our simulations reveal an interplay between primary and secondary extinctions (compare blue to yellow lines, Figure 4g–l) that can alter the sequence of species loss, with synergistic, antagonistic, or neutral ramifications for ecosystem functioning (yellow lines, Figure 4g–l). Indeed, the inclusion of co‐extinction can either increase (B17‐B16, Xs‐B14, B14‐B13, and B17‐B13), decrease (B15‐Xs), or have little effect on the preferential removal of the most vulnerable species (compare blue to yellow line slopes, Figure 4g–l). Thus, realised extinction risk is a product of both co‐dependency and climate‐driven forcing.

## 4 Discussion

Our simulations show that the ecological consequences of species loss associated with climate change reflect the extent to which species traits covary with extinction risk (Solan et al. 2004), the compensatory response of the surviving species (Thomsen et al. 2017; Thomsen et al. 2019), and the modifying role of environmental context (Garcia et al. 2021; Albouy et al. 2019). However, they also emphasise the previously unaccounted role of co‐extinction in adjusting the number of species simultaneously being established and/or extirpated, the realised level of extinction risk and the order of sequential species loss, each having substantive repercussions for ecosystem functioning (Luza et al. 2024). This is important because, when species co‐dependencies are acknowledged, they lead to different biodiversity–function trajectories to those that are currently anticipated, lending support to the view that improved levels of ecological realism are necessary to support the generation of robust environmental futures (Naeem 2008; Garcia et al. 2021; Dolbeth et al. 2019; Gammal et al. 2020). Here, we embraced the modifying effects of biotic interactions on ecological performance (Montoya and Raffaelli 2010; Blois et al. 2013), where the rearrangement of species traits and changes in dominance patterns (Wohlgemuth et al. 2017) within the post‐extinction community are not solely a function of specific extinctions and associated compensatory responses by the surviving community.

Co‐extinctions are expected to hasten species loss (Dunn et al. 2009; Memmott et al. 2004) and minimise functional diversity (Sellman et al. 2016). Our model simulations reveal amplified, sharper losses of biodiversity and, subsequently, ecosystem functioning, indicating an erosion of functional capacity. Though this is in broad agreement with global simulations (Strona and Bradshaw 2018; Strona and Bradshaw 2022), we recognise that the effects of secondary extinctions on ecosystem functioning are likely to vary between populations and environmental settings (Wohlgemuth et al. 2017). Nevertheless, species that are assumed to be resilient based on single risk factors (Leonardsson et al. 2015; Ducatez and Shine 2017; Di Marco et al. 2018), but are co‐dependent on other species, may be compromised (Sanders et al. 2024) or lost earlier than anticipated. Such resequencing may lead to divergent levels of ecosystem functioning depending on how functionally pivotal the extirpated species are within the same context (Fetzer et al. 2015). In regions experiencing amplified levels of climate change such as the Arctic, whether the functional architecture of communities leads to the decline, maintenance, or enhancement of ecosystem functioning will also be dependent on the extent of species immigration, post‐borealisation species interactions (and resulting compensatory responses; Thomsen et al. 2017; Thomsen et al. 2019) and the level of functional redundancy within replacement taxa (Garcia et al. 2021). Here, we find that incorporating multi‐taxa compensatory mechanisms sufficiently reduces the ecological consequences of species loss in each of our scenarios and lessens the effect of losing dominant, highly productive species from local communities with low functional redundancy. Further, we note that the rate of introduction of species from the wider species pool can match or exceed the rate of extinction experienced within the same habitat (Ellis et al. 2012; Sax et al. 2002), leading to stasis or an increase in local biodiversity, with concomitant effects on functioning. As higher diversity is often assumed to have a positive effect on ecosystems, a naïve evaluation might view the latter as a positive, albeit unintuitive, ecosystem response to external forcing (Salo and Gustafsson 2016; Arese Lucini et al. 2020). Yet, our results indicate that the effect of increased biodiversity on ecosystem functioning, particularly when above the currently observed species richness, can be highly variable and, likely, transitory, as changing circumstances further moderate species co‐dependencies and final carrying capacity (Woodworth‐Jefcoats et al. 2017). For example, species introductions can exacerbate native species extinctions (Catford et al. 2018), especially when introduced species are predators or pathogens (Pyšek et al. 2017) and/or cause competitive release (Castorani and Hovel 2015). The latter, however, is not expected to be widespread (Davis 2003) and will be influenced by the effects of environmental context (Melbourne et al. 2007).

Whilst our simulations predict a decline in ecosystem functioning with increased ‘borealisation’ across all our scenarios, the weakest effect occurs at the polar front transition. One explanation might be that the mixing of species and functional groups from the northern and southern species pools (Solan et al. 2020) delays a reduction in functional diversity (Frainer et al. 2017; Frainer et al. 2021). Yet, it is also possible that environmental variation associated with the juxtaposition of water masses will condition resilience (Keith et al. 2008; Renes et al. 2020; Hillebrand et al. 2010). This finding is important because it argues that complex relationships exist between temporal patterns of species turnover and extinction risk, reinforcing the view that species can endure climate change‐associated extinction by persisting in spatio‐temporal refugia (Maclean et al. 2015). As our study design allowed us to compare the response of northern and southern species pools (Solan et al. 2020; Jørgensen et al. 2015), we were able to establish that a subset of taxa dominates species contributions to functioning and that, despite high numbers of compensating species, the greatest functional losses tend to occur at low levels of perturbation. Hence, the potential for compensatory and co‐dependent mechanisms to buffer the consequences of biodiversity loss will depend on the level and extent of functional redundancy (Naeem and Wright 2003)—here, maximised at the polar front—and the net functional role of ingoing and outgoing taxa (Garcia et al. 2021). Interventions aimed at maintaining or improving ecosystem functioning may, therefore, be best placed at the outermost edges of the species pool and/or where environmental conditions are less stochastic (Gerber et al. 2003).

A contemporary focus in ecology is deciphering variations in the relationship between biodiversity and ecosystem function across local and regional spatio‐temporal scales (Gonzalez et al. 2020). Our findings reveal that the shape, magnitude and variability of post‐extinction community functioning are moderated by local environmental conditions (Ratcliffe et al. 2017) and acknowledge the significance of environmental heterogeneity (Wohlgemuth et al. 2017; Bulling et al. 2008; Boyd et al. 2016; Gammal et al. 2020), species arrangement (Wohlgemuth et al. 2016), vulnerability (Kortsch et al. 2015) and the differential expression of response traits (Cassidy et al. 2020; Sanders et al. 2024; Williams et al. 2024). We contend that management and conservation efforts will benefit from considering how and when species responses to external pressures result in changes to extinction risk and alter functional outcomes. Progression in this area will require a transition from conducting before‐after extinction assessments to undertaking stepwise assessments that consider the full and graduated extent of progressive forcing (Fukami and Wardle 2005). It will also require assembly of detailed information about multitrophic network interactions for communities of interest and empirical tests of model findings to refine model construction. As we demonstrate here, the functional consequences of biodiversity loss appear to be gradual and cumulative, but the rate, direction, and magnitude of ecological change can be positively or negatively modified by species co‐dependencies even as the expression of pressures intensifies (Hillebrand et al. 2010).

## Author Contributions

**Thomas J. Williams:** conceptualization, formal analysis, investigation, methodology, project administration, software, visualization, writing – original draft, writing – review and editing. **Clement R. Garcia:** conceptualization, formal analysis, investigation, methodology, software, supervision, validation, writing – review and editing. **Jasmin A. Godbold:** investigation, supervision, validation, writing – review and editing. **Philippe Archambault:** supervision, validation, writing – review and editing. **Martin Solan:** conceptualization, data curation, funding acquisition, methodology, resources, supervision, validation, writing – original draft, writing – review and editing.

## Ethics Statement

This study was approved by the University of Southampton Animal Welfare and Ethical Review Body (ERGO II, #64402). For the purpose of open access, the author has applied a CC BY public copyright licence to any Author Accepted Manuscript version arising from this submission.

## Conflicts of Interest

The authors declare no conflicts of interest.

## Supporting information


**Data S1, Figures S1–S6, Tables S1–S5, Code S1–S8:** Supporting Information.

## Data Availability

The data and code that support the findings of this study are openly available in figshare at https://doi.org/10.6084/m9.figshare.28062653. Benthic invertebrate macrofauna data were obtained from the UK Polar Data Centre at https://doi.org/10.5285/7FBCA0A1‐E2C1‐4265‐A7A5‐713451CB52C0.
