# Supplementary material for: Co‐Extinctions and Co‐Compensatory Species Responses to Climate Change Moderate Ecosystem Futures
Source: Glob Chang Biol. 2025 Oct 8;31(10):e70539. doi: 10.1111/gcb.70539 (PMC12506852; doi:10.1111/gcb.70539)
Supplement: Supplementary file 1 — Data S1, Figures S1–S6, Tables S1–S5, Code S1–S8: Supporting Information. [file GCB-31-e70539-s001.pdf]

Williams, Garcia, Archambault, Godbold, Solan. **Co-extinctions and co-compensatory species responses to climate change moderate ecosystem futures** *Glob. Change Biol.*

Data records S1.

Data records are available *via* an unrestricted repository hosted by the Discovery Metadata System (https://www.bas.ac.uk/project/dms/), a data catalogue hosted by The UK Polar Data Centre (UK PDC, https://www.bas.ac.uk/data/uk-pdc/).

The following data records were used in this contribution:

Invertebrate macrofauna (taxa identity, abundance and biomass)

Solan, M., Godbold, J., Grange, L., Ward, E. R., Wood, C., & Reed, A. (2020). Macrofaunal abundance and biomass for replicate macrofaunal communities from the Western Barents Sea for summer 2017 and 2018 (Version 1.0) [Data set]. UK Polar Data Centre, Natural Environment Research Council, UK Research & Innovation. https://doi.org/10.5285/7FBCA0A1-E2C1-4265-A7A5-713451CB52C0

The cruise reports (RRS James Clarke Ross, JR16006 and JR17007) are available here:

https://www.bodc.ac.uk/resources/inventories/cruise_inventory/reports/jr16006.pdf

https://www.bodc.ac.uk/resources/inventories/cruise_inventory/reports/jr17007.pdf

Figure S1 | Geographical distribution of benthic stations (B13-B14, Xs, B15-B17) and prevailing Arctic (blue arrows) and Atlantic (red arrows) oceanographic currents (Vihtakari et al. 2019; Eriksen et al. 2018) overlain with (*a*) bottom temperature range (˚C, colour rendering) between 2004-2014 and (*b*) mean sea ice cover (fraction of coverage) between 2004-2014 obtained from BioOracle (Tyberghein et al. 2012; Assis et al. 2018) and mapped using the "*sdmpredictors*" R package (Bosch & Fernandez, 2021).

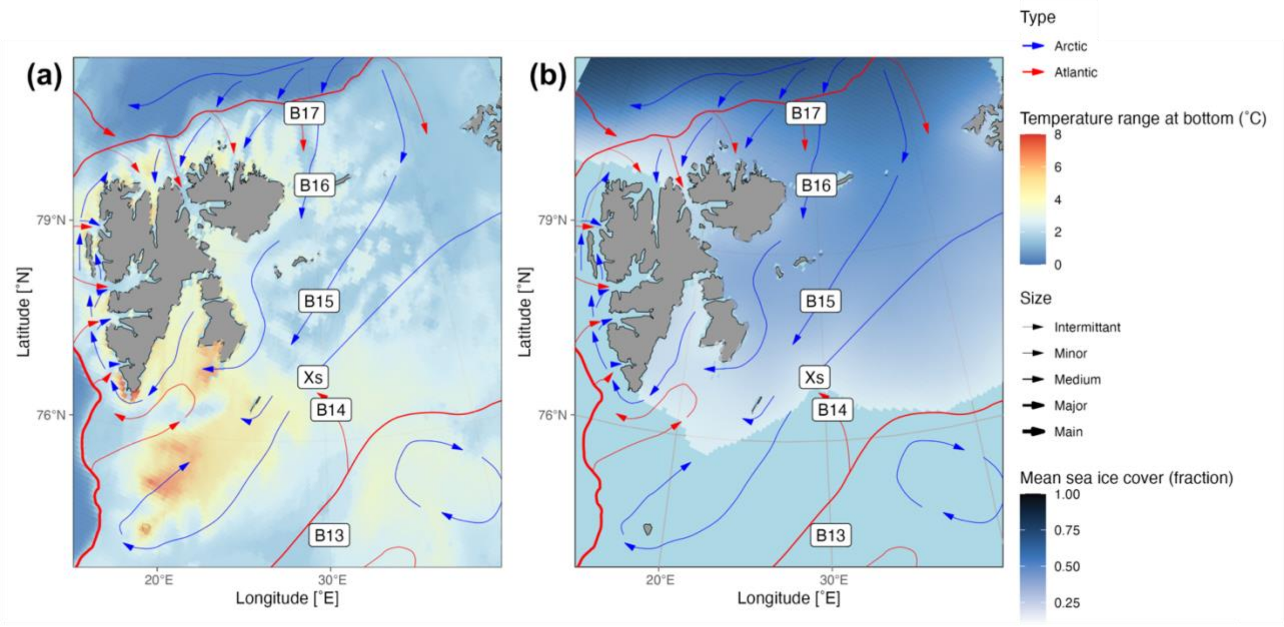

Table S1 | Summary statistics for all taxa found in the regional species pool (n = 113), including the mobility mode (M_i), sediment reworking mode (R_i), trait classification sources and closest taxonomy match for traits, number of stations each species was found at ($n_{station}$), their root-transformed mean body-size (across the entire transect; $B_i^{0.5}$), and abundance (A_i, mean ± s.d. and range).

**Species**	Phylum	Class	M_i	R_i	Traits source	Match	$n_{station}$	$B_i^{0.5}$	A_i **mean ± s.d.**	A_i **min - max**
Abyssoninoe hibernica	Annelida	Polychaeta	3	4	Queiros et al. 2013	Species	2	0.047	12.5 ± 8.839	6.25 – 18.75
Adontorhi juv	Mollusca	Bivalvia	3	2	Degen et al. 2019	Genus	4	0.022	45.313 ± 37.974	12.5 – 100
Aglaophamus malmgreni	Annelida	Polychaeta	3	4	Queiros et al. 2013	Genus	3	0.280	14.583 ± 9.547	6.25 – 25
Ampelisca sp	Arthropoda	Malacostraca	1	2	Queiros et al. 2013	Genus	1	0.098	6.25 ± 0	6.25 – 6.25
Ampeliscidae	Arthropoda	Malacostraca	1	2	Queiros et al. 2013	Genus	1	0.049	31.25 ± 0	31.25 – 31.25
Ampharete finmarchica	Annelida	Polychaeta	2	3	Queiros et al. 2013	Species	1	0.174	6.25 ± 0	6.25 – 6.25
Ampharete lindstroemi	Annelida	Polychaeta	2	3	Queiros et al. 2013	Species	1	0.315	6.25 ± 0	6.25 – 6.25
Ampharete sp	Annelida	Polychaeta	2	3	Queiros et al. 2013	Species	1	0.022	6.25 ± 0	6.25 – 6.25
Amphitrite groenlandica	Annelida	Polychaeta	1	3	Degen et al. 2019	Species	1	1.140	6.25 ± 0	6.25 – 6.25
Amphiuridae indet	Echinodermata	Ophiuroidea	3	4	Queiros et al. 2013	Genus	1	0.135	6.25 ± 0	6.25 – 6.25
Antalis entalis	Mollusca	Scaphopoda	2	3	Queiros et al. 2013	Species	3	0.161	12.5 ± 6.25	6.25 – 18.75
Aphelochaeta marioni	Annelida	Polychaeta	2	2	Queiros et al. 2013	Species	1	0.069	6.25 ± 0	6.25 – 6.25
Aphroditoidea indet	Annelida	Polychaeta	3	4	Queiros et al. 2013	Genus	1	0.077	12.5 ± 0	12.5 – 12.5
Apseudes sp	Arthropoda	Malacostraca	2	2	Queiros et al. 2013	Genus	1	0.010	6.25 ± 0	6.25 – 6.25
Aricidea catherie	Annelida	Polychaeta	3	2	Queiros et al. 2013	Species	5	0.019	16.25 ± 7.126	6.25 – 25
Aricidea quadrilobata	Annelida	Polychaeta	3	2	Queiros et al. 2013	Genus	2	0.055	9.375 ± 4.419	6.25 – 12.5
Aricidea suecica	Annelida	Polychaeta	3	2	Queiros et al. 2013	Species	2	0.013	15.625 ± 4.419	12.5 – 18.75
Ascelerichilus intermedius	Annelida	Polychaeta	4	4	Queiros et al. 2013	Species	3	0.099	8.333 ± 3.608	6.25 – 12.5
Astarte creta agg	Mollusca	Bivalvia	2	2	Williams et al. 2024	Species	4	0.366	6.25 ± 0	6.25 – 6.25
Autolytie indet	Annelida	Polychaeta	3	1	Solan et al. 2020	Subfamily	1	0.010	6.25 ± 0	6.25 – 6.25
Bathyarca frielei	Mollusca	Bivalvia	2	2	Degen et al. 2019	Species	2	0.234	9.375 ± 4.419	6.25 – 12.5
Bathyarca glacialis	Mollusca	Bivalvia	2	2	Degen et al. 2019	Genus	1	1.225	6.25 ± 0	6.25 – 6.25
Brachydiastylis resima	Arthropoda	Malacostraca	3	2	Queiros et al. 2013	Species	4	0.042	6.25 ± 0	6.25 – 6.25
Calathura norvegica	Arthropoda	Malacostraca	3	1	Solan et al. 2020	Species	1	0.063	6.25 ± 0	6.25 – 6.25
Calathura sp	Arthropoda	Malacostraca	3	1	Solan et al. 2020	Genus	1	0.050	6.25 ± 0	6.25 – 6.25

Capitella sp	Annelida	Polychaeta	3	3	Morys et al. 2017	Genus	1	0.022	18.75 ± 0	18.75 – 18.75
Caulleriella sp A	Annelida	Polychaeta	2	2	Queiros et al. 2013	Genus	1	0.050	6.25 ± 0	6.25 – 6.25
Chaetozone setosa	Annelida	Polychaeta	2	2	Queiros et al. 2013	Species	5	0.039	12.5 ± 10.825	6.25 – 31.25
Chirimia biceps	Annelida	Polychaeta	2	3	Queiros et al. 2013	Species	2	0.200	118.75 ± 44.194	87.5 – 150
Cirrophorus eliasoni	Annelida	Polychaeta	3	2	Queiros et al. 2013	Genus	1	0.020	12.5 ± 0	12.5 – 12.5
Cistenides hyperborea	Annelida	Polychaeta	4	3	Williams et al. 2024	Species	3	0.278	8.333 ± 3.608	6.25 – 12.5
Clymenura polaris	Annelida	Polychaeta	1	3	Queiros et al. 2013	Species	1	0.062	6.25 ± 0	6.25 – 6.25
Clymenura sp	Annelida	Polychaeta	1	3	Queiros et al. 2013	Genus	1	0.049	12.5 ± 0	12.5 – 12.5
Cossura sp	Annelida	Polychaeta	3	2	Queiros et al. 2013	Genus	2	0.022	6.25 ± 0	6.25 – 6.25
Ctenodiscus crispatus	Echinodermata	Asteroidea	3	2	Williams et al. 2024	Species	5	0.408	11.25 ± 6.847	6.25 – 18.75
Cuspidaria obesa	Mollusca	Bivalvia	2	3	Queiros et al. 2013	Species	1	0.197	6.25 ± 0	6.25 – 6.25
Dialychone spp	Annelida	Polychaeta	1	2	Queiros et al. 2013	Genus	2	0.064	15.625 ± 13.258	6.25 – 25
Diastylis lucifera	Arthropoda	Malacostraca	3	2	Queiros et al. 2013	Family	1	0.021	12.5 ± 0	12.5 – 12.5
Diastyloides biplicata	Arthropoda	Malacostraca	3	2	Queiros et al. 2013	Family	1	0.082	6.25 ± 0	6.25 – 6.25
Diplocirrus hirsutus	Annelida	Polychaeta	2	3	Queiros et al. 2013	Genus	3	0.079	10.417 ± 7.217	6.25 – 18.75
Dipolydora sp	Annelida	Polychaeta	3	4	Queiros et al. 2013	Genus	1	0.032	6.25 ± 0	6.25 – 6.25
Edwardsia sp	Arthropoda	Copepoda	3	2	Degen et al. 2019	Genus	1	0.356	6.25 ± 0	6.25 – 6.25
Ennucula tenuis	Mollusca	Bivalvia	3	2	Queiros et al. 2013	Species	2	0.084	6.25 ± 0	6.25 – 6.25
Ephesiella abyssorum	Annelida	Polychaeta	3	4	Queiros et al. 2013	Species	1	0.194	6.25 ± 0	6.25 – 6.25
Eteone longa	Annelida	Polychaeta	3	4	Morys et al. 2017	Species	1	0.035	6.25 ± 0	6.25 – 6.25
Eteone sp	Annelida	Polychaeta	3	4	Morys et al. 2017	Genus	2	0.024	6.25 ± 0	6.25 – 6.25
Euclymene droebachiensis	Annelida	Polychaeta	1	3	Queiros et al. 2013	Species	1	0.274	12.5 ± 0	12.5 – 12.5
Eudorella emargita	Arthropoda	Malacostraca	3	2	Queiros et al. 2013	Species	1	0.082	6.25 ± 0	6.25 – 6.25
Exogone sp	Annelida	Polychaeta	3	4	Queiros et al. 2013	Genus	1	0.010	6.25 ± 0	6.25 – 6.25
Galathowenia oculata	Annelida	Polychaeta	1	2	Queiros et al. 2013	Genus	2	0.036	21.875 ± 13.258	12.5 – 31.25
Glyphanostomum pallescens	Annelida	Polychaeta	1	3	Queiros et al. 2013	Species	1	0.026	12.5 ± 0	12.5 – 12.5
Gthia elongata	Arthropoda	Malacostraca	3	2	Queiros et al. 2013	Genus	3	0.025	8.333 ± 3.608	6.25 – 12.5
Gthia maxillaris	Arthropoda	Malacostraca	3	2	Queiros et al. 2013	Species	3	0.024	8.333 ± 3.608	6.25 – 12.5
Gthiidae	Arthropoda	Malacostraca	3	2	Queiros et al. 2013	Genus	1	0.024	18.75 ± 0	18.75 – 18.75
Haploops setosa	Arthropoda	Malacostraca	1	2	Queiros et al. 2013	Genus	1	0.062	12.5 ± 0	12.5 – 12.5

Haploops tubicola	Arthropoda	Malacostraca	1	2	Queiros et al. 2013	Species	5	0.049	36.25 ± 57.18	6.25 – 137.5
Harpinia antenria	Arthropoda	Malacostraca	3	2	Queiros et al. 2013	Species	1	0.037	6.25 ± 0	6.25 – 6.25
Harpinia sp	Arthropoda	Malacostraca	3	2	Queiros et al. 2013	Genus	1	0.017	6.25 ± 0	6.25 – 6.25
Heteromastus filiformis	Annelida	Polychaeta	2	3	Gogina et al. 2016	Species	2	0.018	18.75 ± 17.678	6.25 – 31.25
Hippomedon sp	Arthropoda	Malacostraca	3	2	Queiros et al. 2013	Genus	1	0.057	6.25 ± 0	6.25 – 6.25
Leitoscoloplos mammosus	Annelida	Polychaeta	3	4	Queiros et al. 2013	Species	4	0.037	48.438 ± 47.701	12.5 – 118.75
Leptogthia gracilis	Arthropoda	Malacostraca	3	2	Degen et al. 2019	Species	1	0.030	6.25 ± 0	6.25 – 6.25
Leucon sica	Arthropoda	Malacostraca	3	2	Queiros et al. 2013	Species	3	0.043	6.25 ± 0	6.25 – 6.25
Levinsenia gracillis	Annelida	Polychaeta	3	2	Queiros et al. 2013	Species	4	0.024	37.5 ± 34.233	12.5 – 87.5
Lumbrineris mixochaeta	Annelida	Polychaeta	3	4	Queiros et al. 2013	Genus	6	0.064	75 ± 43.839	31.25 – 143.75
Lumbrinidae indet	Annelida	Polychaeta	3	4	Queiros et al. 2013	Genus	1	0.037	18.75 ± 0	18.75 – 18.75
Lysippe sexcirrata	Annelida	Polychaeta	1	3	Degen et al. 2019	Species	1	0.028	6.25 ± 0	6.25 – 6.25
Maldane sarsi	Annelida	Polychaeta	1	3	Degen et al. 2019	Species	5	0.072	295 ± 339.502	37.5 – 781.25
Maldanidae indet	Annelida	Polychaeta	1	3	Queiros et al. 2013	Genus	1	0.213	6.25 ± 0	6.25 – 6.25
Mediomastus fragilis	Annelida	Polychaeta	2	3	Queiros et al. 2013	Species	4	0.026	46.875 ± 24.206	18.75 – 75
Melin sp	Mollusca	Bivalvia	1	3	Queiros et al. 2013	Genus	2	0.104	9.375 ± 4.419	6.25 – 12.5
Myriochele heeri	Annelida	Polychaeta	1	2	Queiros et al. 2013	Species	5	0.035	10 ± 3.423	6.25 – 12.5
Myriochele sp	Annelida	Polychaeta	1	2	Queiros et al. 2013	Genus	1	0.028	37.5 ± 0	37.5 – 37.5
Nematoda	Nematoda		2	2	Queiros et al. 2013	Genus	5	0.008	51.25 ± 39.873	6.25 – 93.75
Nemertea	Nemertea		4	4	Queiros et al. 2013	Genus	5	0.335	10 ± 5.59	6.25 – 18.75
Nephasoma procera	Annelida	Sipuncula	3	4	Queiros et al. 2013	Genus	5	0.037	135 ± 188.466	12.5 – 462.5
Nephtys ciliata	Annelida	Polychaeta	3	4	Gogina et al. 2016	Family	1	0.073	12.5 ± 0	12.5 – 12.5
Nephtys incisa	Annelida	Polychaeta	3	4	Queiros et al. 2013	Family	3	0.292	18.75 ± 6.25	12.5 – 25
Nephtys juv	Annelida	Polychaeta	3	4	Queiros et al. 2013	Family	3	0.029	6.25 ± 0	6.25 – 6.25
Opheli abranchiata	Annelida	Polychaeta	3	4	Queiros et al. 2013	Genus	2	0.031	15.625 ± 13.258	6.25 – 25
Ophiocten sericeum	Echinodermata	Ophiuroidea	2	2	Queiros et al. 2013	Genus	1	0.211	6.25 ± 0	6.25 – 6.25
Ostracoda	Arthropoda	Ostracoda	3	2	Degen et al. 2019	Class	3	0.023	29.167 ± 29.536	6.25 – 62.5
Owenia polaris	Annelida	Polychaeta	1	2	Queiros et al. 2013	Genus	1	0.042	181.25 ± 0	181.25 – 181.25
Paradoneis sp	Annelida	Polychaeta	3	2	Queiros et al. 2013	Genus	1	0.010	6.25 ± 0	6.25 – 6.25
Paramphinome jeffreysii	Annelida	Polychaeta	3	4	Queiros et al. 2013	Species	1	0.047	6.25 ± 0	6.25 – 6.25

Paraonidae indet	Annelida	Polychaeta	3	2	Queiros et al. 2013	Genus	2	0.049	6.25 ± 0	6.25 – 6.25
Paraonides sp	Annelida	Polychaeta	3	2	Queiros et al. 2013	Genus	1	0.028	6.25 ± 0	6.25 – 6.25
Parougia caeca	Annelida	Polychaeta	3	4	Queiros et al. 2013	Species	1	0.053	6.25 ± 0	6.25 – 6.25
Pellecepoda	Mollusca	Pelecypoda	2	2	Solan et al. 2020	Class	2	0.044	56.25 ± 61.872	12.5 – 100
Phascolion strombi	Annelida	Sipuncula	2	2	Queiros et al. 2013	Species	1	0.145	6.25 ± 0	6.25 – 6.25
Pherusa plumosa	Annelida	Polychaeta	2	3	Queiros et al. 2013	Species	1	0.346	6.25 ± 0	6.25 – 6.25
Pholoidae indet	Annelida	Polychaeta	2	2	Queiros et al. 2013	Family	1	0.024	6.25 ± 0	6.25 – 6.25
Phyllodoce groenlandica	Annelida	Polychaeta	3	4	Solan et al. 2004	Species	2	0.081	6.25 ± 0	6.25 – 6.25
Polycirrus arcticus	Annelida	Polychaeta	1	3	Queiros et al. 2013	Genus	1	0.056	6.25 ± 0	6.25 – 6.25
Praxiella gracilis	Annelida	Polychaeta	1	3	Queiros et al. 2013	Species	2	0.244	9.375 ± 4.419	6.25 – 12.5
Praxillura longissima	Annelida	Polychaeta	1	3	Solan et al. 2020	Species	1	0.182	6.25 ± 0	6.25 – 6.25
Prionospio cirrifera	Annelida	Polychaeta	2	3	Queiros et al. 2013	Species	3	0.030	8.333 ± 3.608	6.25 – 12.5
Quasimelita quadrispinosa	Arthropoda	Malacostraca	4	3	Solan et al. 2020	Species	1	0.191	6.25 ± 0	6.25 – 6.25
Retusa obtusa	Mollusca	Gastropoda	3	2	Queiros et al. 2013	Species	2	0.086	9.375 ± 4.419	6.25 – 12.5
Rhodine gracilor	Annelida	Polychaeta	1	3	Queiros et al. 2013	Species	2	0.070	6.25 ± 0	6.25 – 6.25
Rhodine sp	Annelida	Polychaeta	1	3	Queiros et al. 2013	Genus	1	0.052	12.5 ± 0	12.5 – 12.5
Scolelepis sp	Annelida	Polychaeta	2	3	Queiros et al. 2013	Genus	1	0.071	6.25 ± 0	6.25 – 6.25
Spiochaetopterus typicus	Annelida	Polychaeta	1	3	Queiros et al. 2013	Species	6	0.103	466.667 ± 550.08	12.5 – 1350
Spiophanes bombyx	Annelida	Polychaeta	1	3	Queiros et al. 2013	Species	1	0.059	6.25 ± 0	6.25 – 6.25
Spiophanes kroyeri	Annelida	Polychaeta	1	3	Queiros et al. 2013	Species	6	0.040	63.542 ± 54.974	6.25 – 156.25
Spirorbie indet	Annelida	Polychaeta	1	3	Queiros et al. 2013	Genus	1	0.010	6.25 ± 0	6.25 – 6.25
Syllis cornuta agg	Annelida	Polychaeta	3	4	Queiros et al. 2013	Species	2	0.050	15.625 ± 4.419	12.5 – 18.75
Syllis sp	Annelida	Polychaeta	3	2	Queiros et al. 2013	Species	2	0.077	6.25 ± 0	6.25 – 6.25
Syllis sp E	Annelida	Polychaeta	3	2	Queiros et al. 2013	Species	1	0.054	6.25 ± 0	6.25 – 6.25
Taidacea	Arthropoda	Malacostraca	3	2	Degen et al. 2019	Order	3	0.033	6.25 ± 0	6.25 – 6.25
Terebellides stroemii	Annelida	Polychaeta	1	3	Queiros et al. 2013	Species	4	0.095	7.813 ± 3.125	6.25 – 12.5
Terebratuli retusa	Brachiopoda	Terebratulida	1	1	Degen et al. 2019	Species	1	0.229	6.25 ± 0	6.25 – 6.25
Yoldiidae	Mollusca	Bivalvia	3	2	Queiros et al. 2013	Family	6	0.076	118.75 ± 181.358	6.25 – 456.25

Table S2 | Ranked (rk) vulnerabilities (alphabetically ordered) to climate-driven environmental transitions (right hand columns) based on percentage differences (left hand columns) in sediment-dwelling invertebrate biomass between the pre-extinction community (northernmost station) and the reference post-extinction community (southernmost station) for all taxa in the regional species pool (n = 113). Inf. denotes where there is no biomass in the pre-extinction community and biomass in the post-extinction community, so percentage difference = infinite.

**Species**	**B17-B16**	**B16-B15**	**B15-Xs**	**Xs-B14**	**B14-B13**	**B17-B13**	**rk(B17-B16)**	**rk(B16-B15)**	**rk(B15-Xs)**	**rk(Xs-B14)**	**rk(B14-B13)**	**rk(B17-B13)**
Abyssoninoe hibernica	Inf	1.8261	-1	0	0	0	104	88	13	69	52	67
Adontorhina juv	-1	0	Inf	-0.942	0.3	0.3	15	60	100.5	25	84	90
Aglaophamus malmgreni	-1	0	Inf	-0.929	-1	-1	15	60	100.5	26	8	17
Ampelisca sp	0	0	0	Inf	-1	0	60.5	60	55	108.5	8	67
Ampeliscidae	Inf	-1	0	0	0	0	104	13.5	55	69	52	67
Ampharete finmarchica	0	0	0	Inf	-1	0	60.5	60	55	108.5	8	67
Ampharete lindstroemi	0	0	Inf	-1	0	0	60.5	60	100.5	12.5	52	67
Ampharete sp	Inf	-1	0	0	0	0	104	13.5	55	69	52	67
Amphitrite groenlandica	-1	0	0	0	0	-1	15	60	55	69	52	17
Amphiuridae indet	-1	0	0	0	0	-1	15	60	55	69	52	17
Antalis entalis	-1	0	0	Inf	17.538	-0.667	15	60	55	108.5	90	38
Aphelochaeta marioni	-1	0	0	0	0	-1	15	60	55	69	52	17

Aphroditoidea indet	-1	0	0	0	0	-1	15	60	55	69	52	17
Apseudes sp	0	0	0	Inf	-1	0	60.5	60	55	108.5	8	67
Aricidea catherinae	3	-0.95	26	-1	Inf	2.8	90	27	87	12.5	102	93
Aricidea quadrilobata	-1	Inf	-1	0	0	-1	15	101.5	13	69	52	17
Aricidea suecica	-1	0	Inf	-1	0	-1	15	60	100.5	12.5	52	17
Ascelerichilus intermedius	0	Inf	-0.966	-1	Inf	Inf	60.5	101.5	26	12.5	102	105.5
Astarte crenata agg	-1	0	Inf	47.815	2.11	3.2084	15	60	100.5	102	86	95
Autolytinae indet	0	0	0	0	Inf	Inf	60.5	60	55	69	102	105.5
Bathyarca frielei	-1	0	0	0	Inf	-0.967	15	60	55	69	102	34
Bathyarca glacialis	0	0	0	0	Inf	Inf	60.5	60	55	69	102	105.5
Brachydiastylis resima	-0.75	-0.5	6	-1	0	-1	32	36	80	12.5	52	17
Calathura norvegica	0	Inf	-1	0	0	0	60.5	101.5	13	69	52	67
Calathura sp	0	Inf	-1	0	0	0	60.5	101.5	13	69	52	67
Capitella sp	Inf	-1	0	0	0	0	104	13.5	55	69	52	67
Caulleriella sp A	-1	0	0	0	0	-1	15	60	55	69	52	17

Chaetozone setosa	-0.44	-0.857	21.5	-1	Inf	0.52	38	31	86	12.5	102	92
Chirimia biceps	-0.49	-1	0	0	0	-1	37	13.5	55	69	52	17
Cirrophorus eliasoni	-1	0	0	0	0	-1	15	60	55	69	52	17
Cistenides hyperborea	0	0	Inf	-0.121	0.6252	Inf	60.5	60	100.5	37	85	105.5
Clymenura polaris	0	Inf	-1	0	0	0	60.5	101.5	13	69	52	67
Clymenura sp	-1	0	0	0	0	-1	15	60	55	69	52	17
Cossura sp	Inf	-1	Inf	-1	0	0	104	13.5	100.5	12.5	52	67
Ctenodiscus crispatus	-0.809	-1	Inf	0.2042	10.217	2.8059	31	13.5	100.5	101	89	94
Cuspidaria obesa	0	Inf	-1	0	0	0	60.5	101.5	13	69	52	67
Dialychone spp	3.5526	-1	0	0	0	-1	91	13.5	55	69	52	17
Diastylis lucifera	-1	0	0	0	0	-1	15	60	55	69	52	17
Diastyloides biplicata	-1	0	0	0	0	-1	15	60	55	69	52	17
Diplocirrus hirsutus	-1	0	Inf	-1	Inf	-0.518	15	60	100.5	12.5	102	41
Dipolydora sp	Inf	-1	0	0	0	0	104	13.5	55	69	52	67
Edwardsia sp	0	0	0	0	Inf	Inf	60.5	60	55	69	102	105.5

Ennucula tenuis	2.8611	-1	0	0	0	-1	89	13.5	55	69	52	17
Ephesiella abyssorum	0	0	Inf	-1	0	0	60.5	60	100.5	12.5	52	67
Eteone longa	0	0	Inf	-1	0	0	60.5	60	100.5	12.5	52	67
Eteone sp	0	0	Inf	-0.5	-1	0	60.5	60	100.5	32	8	67
Euclymene droebachiensis	0	0	0	Inf	-1	0	60.5	60	55	108.5	8	67
Eudorella emarginata	-1	0	0	0	0	-1	15	60	55	69	52	17
Exogone sp	0	Inf	-1	0	0	0	60.5	101.5	13	69	52	67
Galathowenia oculata	Inf	-1	Inf	-1	0	0	104	13.5	100.5	12.5	52	67
Glyphanostomum pallescens	Inf	-1	0	0	0	0	104	13.5	55	69	52	67
Gnathia elongata	6.25	-0.793	-1	0	0	-1	92	34	13	69	52	17
Gnathia maxillaris	-0.286	-1	0	0	Inf	0.4286	39	13.5	55	69	102	91
Gnathiidae	Inf	-1	0	0	0	0	104	13.5	55	69	52	67
Haploops setosa	0	Inf	-1	0	0	0	60.5	101.5	13	69	52	67
Haploops tubicola	55.1	-1	Inf	-0.364	8.8571	12.8	93	13.5	100.5	35	88	97
Harpinia antennaria	0	Inf	-1	0	0	0	60.5	101.5	13	69	52	67

Harpinia sp	0	Inf	-1	0	0	0	60.5	101.5	13	69	52	67
Heteromastus filiformis	-1	Inf	-1	0	0	-1	15	101.5	13	69	52	17
Hippomedon sp	0	Inf	-1	0	0	0	60.5	101.5	13	69	52	67
Leitoscoloplos mammosus	-0.695	-1	Inf	-1	Inf	-0.118	34	13.5	100.5	12.5	102	43
Leptognathia gracilis	0	Inf	-1	0	0	0	60.5	101.5	13	69	52	67
Leucon nasica	Inf	-1	Inf	-1	Inf	Inf	104	13.5	100.5	12.5	102	105.5
Levinsenia gracillis	-0.646	-1	Inf	-1	Inf	-0.866	35	13.5	100.5	12.5	102	36
Lumbrineris mixochaeta	-0.698	-0.728	6	-0.799	-0.023	-0.887	33	35	81	30	20	35
Lumbrinidae indet	0	0	0	Inf	-1	0	60.5	60	55	108.5	8	67
Lysippe sexcirrata	0	0	0	0	Inf	Inf	60.5	60	55	69	102	105.5
Maldane sarsi	0.5714	-0.854	2.0303	-0.297	-1	-1	85	32	79	36	8	17
Maldanidae indet	0	0	0	Inf	-1	0	60.5	60	55	108.5	8	67
Mediomastus fragilis	0.411	-1	Inf	-1	Inf	-0.521	83	13.5	100.5	12.5	102	40
Melinna sp	-1	0	Inf	-1	0	-1	15	60	100.5	12.5	52	17
Myriochele heeri	1.0833	-0.88	19.333	-0.639	-1	-1	87	30	84	31	8	17

Myriochele sp	0	0	Inf	-1	0	0	60.5	60	100.5	12.5	52	67
Nematoda	0.6	0.875	-0.933	0	-1	-1	86	87	27	69	8	17
Nemertea	-0.998	-1	Inf	234.5	-0.549	-0.736	30	13.5	100.5	103	18	37
Nephasoma procera	0.4002	-0.82	-0.291	-0.808	-1	-1	82	33	30	29	8	17
Nephtys ciliata	0	0	0	0	Inf	Inf	60.5	60	55	69	102	105.5
Nephtys incisa	0	0	Inf	-0.495	-0.949	Inf	60.5	60	100.5	33	16	105.5
Nephtys juv	-1	Inf	-0.667	-1	0	-1	15	101.5	29	12.5	52	17
Ophelina abranchiata	-1	Inf	-1	0	0	-1	15	101.5	13	69	52	17
Ophiocten sericeum	-1	0	0	0	0	-1	15	60	55	69	52	17
Ostracoda	Inf	-0.917	20	-1	0	0	104	29	85	12.5	52	67
Owenia polaris	0	0	Inf	-1	0	0	60.5	60	100.5	12.5	52	67
Paradoneis sp	0	0	0	Inf	-1	0	60.5	60	55	108.5	8	67
Paramphinome jeffreysii	Inf	-1	0	0	0	0	104	13.5	55	69	52	67
Paraonidae indet	0	Inf	-1	0	Inf	Inf	60.5	101.5	13	69	102	105.5
Paraonides sp	0	Inf	-1	0	0	0	60.5	101.5	13	69	52	67

Parougia caeca	0	Inf	-1	0	0	0	60.5	101.5	13	69	52	67
Pellecepoda	-1	Inf	-1	0	0	-1	15	101.5	13	69	52	17
Phascolion strombi	0	Inf	-1	0	0	0	60.5	101.5	13	69	52	67
Pherusa plumosa	0	0	Inf	-1	0	0	60.5	60	100.5	12.5	52	67
Pholoidae indet	0	0	0	0	Inf	Inf	60.5	60	55	69	102	105.5
Phyllodoce groenlandica	Inf	-1	0	0	Inf	Inf	104	13.5	55	69	102	105.5
Polycirrus arcticus	Inf	-1	0	0	0	0	104	13.5	55	69	52	67
Praxiella gracilis	0	0	Inf	-1	Inf	Inf	60.5	60	100.5	12.5	102	105.5
Praxillura longissima	0	0	0	0	Inf	Inf	60.5	60	55	69	102	105.5
Prionospio cirrifera	-1	Inf	-1	0	Inf	-0.6	15	101.5	13	69	102	39
Quasimelita quadrispinosa	0	0	Inf	-1	0	0	60.5	60	100.5	12.5	52	67
Retusa obtusa	Inf	-1	0	0	Inf	Inf	104	13.5	55	69	102	105.5
Rhodine gracilor	0	Inf	-1	Inf	-1	0	60.5	101.5	13	108.5	8	67
Rhodine sp	Inf	-1	0	0	0	0	104	13.5	55	69	52	67
Scolelepis sp	Inf	-1	0	0	0	0	104	13.5	55	69	52	67

Spiochaetopterus typicus	0.4446	0.7086	8.2296	-0.44	-0.273	8.2765	84	85	82	34	19	96
Spiophanes bombyx	-1	0	0	0	0	-1	15	60	55	69	52	17
Spiophanes kroyeri	2.0638	0.8438	-0.817	-0.866	2.9231	-0.457	88	86	28	28	87	42
Spirorbinae indet	0	Inf	-1	0	0	0	60.5	101.5	13	69	52	67
Syllis cornuta agg	-0.543	-1	0	0	0	-1	36	13.5	55	69	52	17
Syllis sp	Inf	0.5	-1	0	0	0	104	84	13	69	52	67
Syllis sp E	-1	0	0	0	0	-1	15	60	55	69	52	17
Tanaidacea	-1	Inf	-1	Inf	-1	-1	15	101.5	13	108.5	8	17
Terebellides stroemii	Inf	2.6857	-0.039	-1	Inf	Inf	104	89	31	12.5	102	105.5
Terebratulina retusa	-1	0	0	0	0	-1	15	60	55	69	52	17
Yoldiidae	164.12	-0.947	14.327	-0.881	-0.945	-0.118	94	28	83	27	17	44

Figure S2 | Log-transformed taxa-specific BP_i, from the most (top) to least (bottom) contributing taxa for all local (B17-B16 | B16-B15 | B15-Xs | Xs-B14 | B14-B13) and regional (B17-B13) environmental transitions.

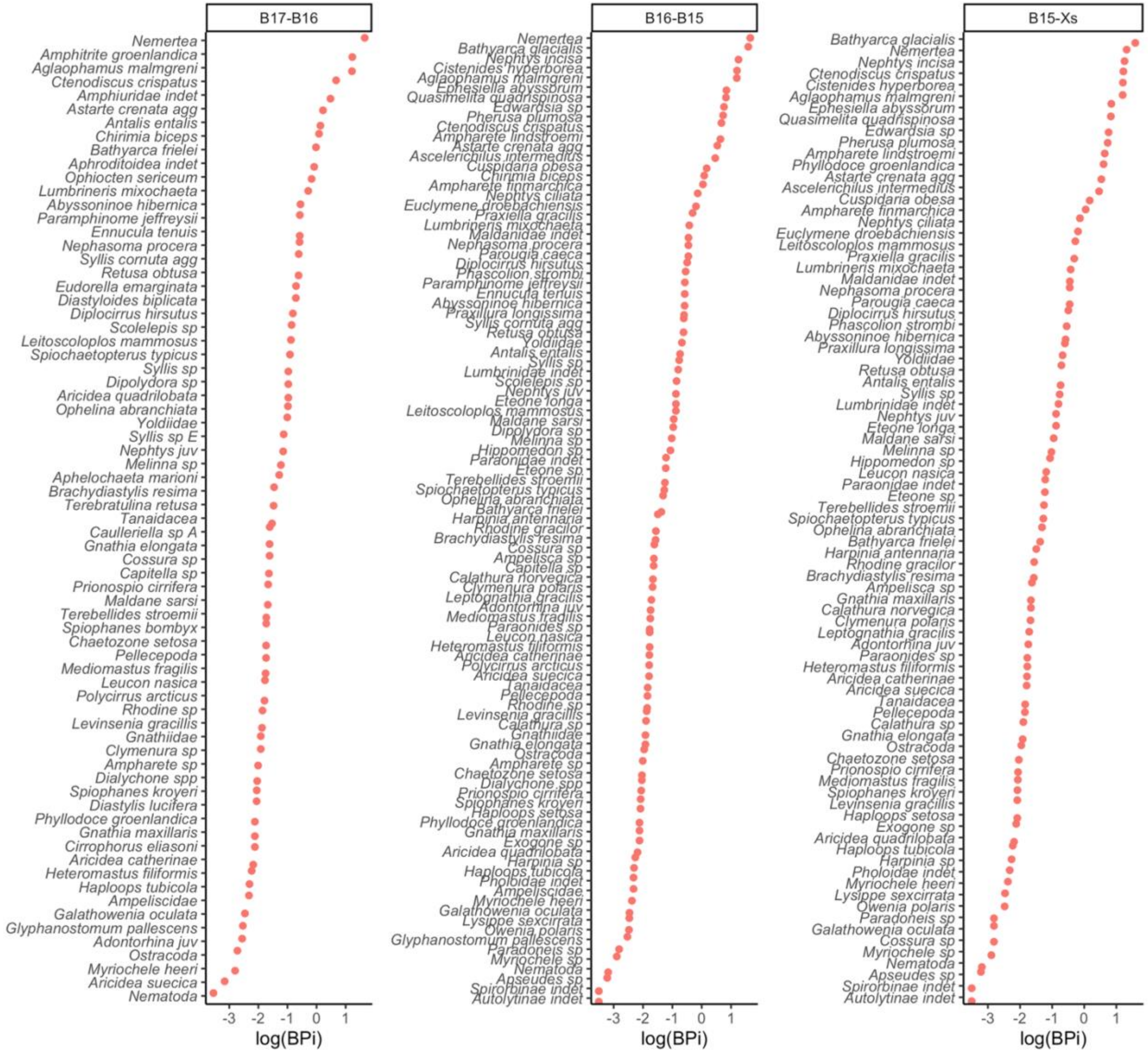

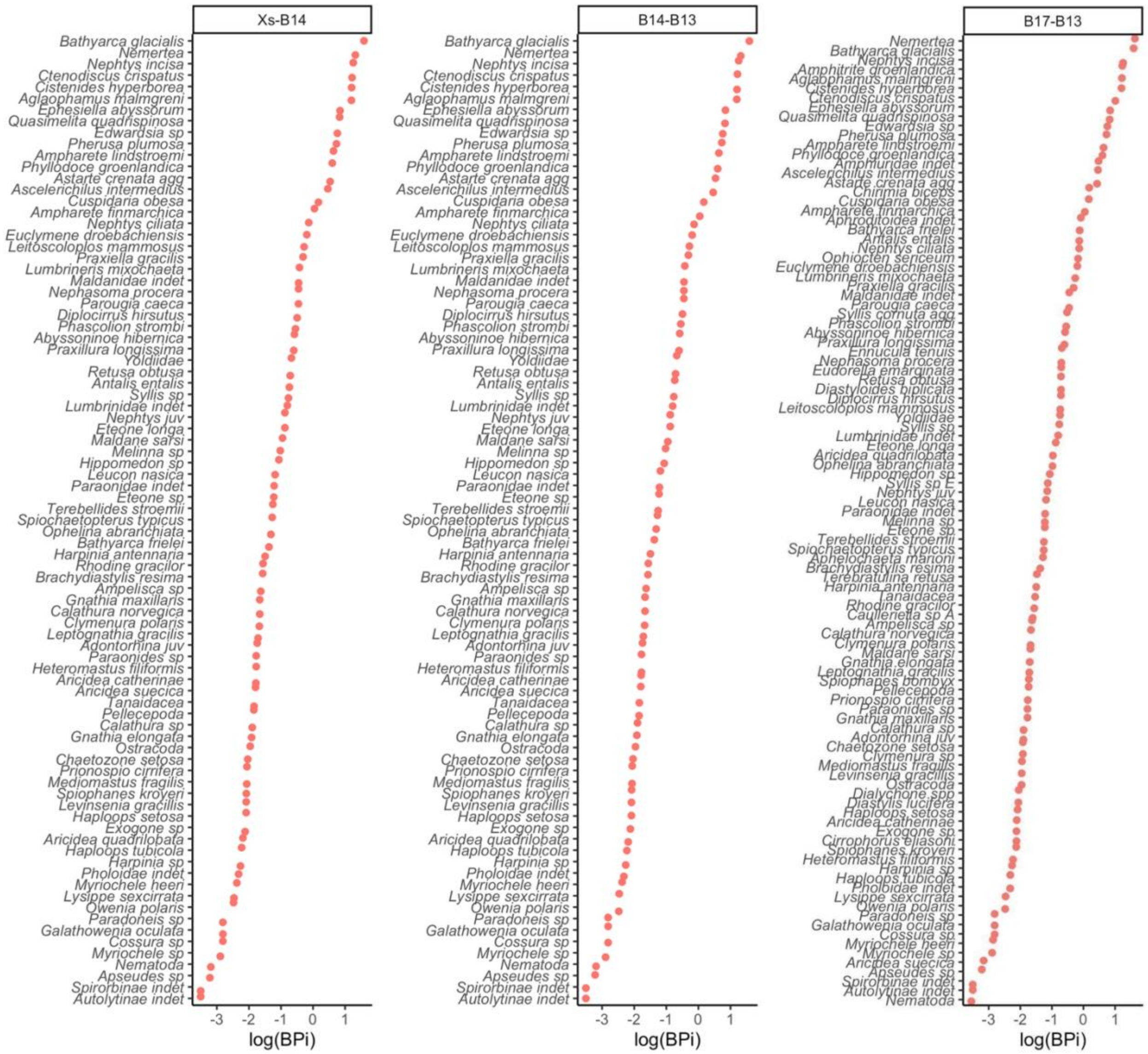
Xs-B14
Bathyarca glacialis
Nemertea
Nephtys incisa
Ctenodiscus crispatus
Cistenides hyperborea
Aglaophamus malmgreni
Ephesiella abyssorum
Quasimelita quadrispinosa
Edwardsia sp
Pherusa plumosa
Ampharete lindstroemi
Phyllodoce groenlandica
Astarte crenata agg
Ascelerichilus intermedius
Cuspidaria obesa
Ampharete finmarchica
Nephtys ciliata
Euclymene droebachiensis
Leitoscoloplos mammosus
Praxiella gracilis
Lumbrineris mixochaeta
Maldanidae indet
Nephasoma procera
Parougia caeca
Diplocirrus hirsutus
Phascolion strombi
Abyssoninoe hibernica
Praxillura longissima
Yoldiidae
Retusa obtusa
Antalis entalis
Syllis sp
Lumbrinidae indet
Nephtys juv
Eteone longa
Maldane sarsi
Melinna sp
Hippomedon sp
Leucon nasica
Paraonidae indet
Eteone sp
Terebellides stroemii
Spiochaetopterus typicus
Ophelina abranchiata
Bathyarca frielei
Harpinia antennaria
Rhodine gracilor
Brachydiastylis resima
Ampelisca sp
Gnathia maxillaris
Calathura norvegica
Clymenura polaris
Leptognathia gracilis
Adontorhina juv
Paraonides sp
Heteromastus filiformis
Aricidea catherinae
Aricidea suecica
Tanaidacea
Pellecepoda
Calathura sp
Gnathia elongata
Ostracoda
Chaetozone setosa
Prionospio cirrifera
Mediomastus fragilis
Spiophanes kroyeri
Levinsenia gracilis
Haploops setosa
Exogone sp
Aricidea quadrilobata
Haploops tubicola
Harpinia sp
Pholoidae indet
Myriochele heeri
Lysippe sexcirrata
Owenia polaris
Paradoneis sp
Galathowenia oculata
Cossura sp
Myriochele sp
Nematoda
Apseudes sp
Spirorbinae indet
Autolytinae indet
-3
-2
-1
0
1
log(BPi)
B14-B13
Bathyarca glacialis
Nemertea
Nephtys incisa
Ctenodiscus crispatus
Cistenides hyperborea
Aglaophamus malmgreni
Ephesiella abyssorum
Quasimelita quadrispinosa
Edwardsia sp
Pherusa plumosa
Ampharete lindstroemi
Phyllodoce groenlandica
Astarte crenata agg
Ascelerichilus intermedius
Cuspidaria obesa
Ampharete finmarchica
Nephtys ciliata
Euclymene droebachiensis
Leitoscoloplos mammosus
Praxiella gracilis
Lumbrineris mixochaeta
Maldanidae indet
Nephasoma procera
Parougia caeca
Diplocirrus hirsutus
Phascolion strombi
Abyssoninoe hibernica
Praxillura longissima
Yoldiidae
Retusa obtusa
Antalis entalis
Syllis sp
Lumbrinidae indet
Nephtys juv
Eteone longa
Maldane sarsi
Melinna sp
Hippomedon sp
Leucon nasica
Paraonidae indet
Eteone sp
Terebellides stroemii
Spiochaetopterus typicus
Ophelina abranchiata
Bathyarca frielei
Harpinia antennaria
Rhodine gracilor
Brachydiastylis resima
Ampelisca sp
Gnathia maxillaris
Calathura norvegica
Clymenura polaris
Leptognathia gracilis
Adontorhina juv
Paraonides sp
Heteromastus filiformis
Aricidea catherinae
Aricidea suecica
Tanaidacea
Pellecepoda
Calathura sp
Gnathia elongata
Ostracoda
Chaetozone setosa
Prionospio cirrifera
Mediomastus fragilis
Spiophanes kroyeri
Levinsenia gracilis
Haploops setosa
Exogone sp
Aricidea quadrilobata
Haploops tubicola
Harpinia sp
Pholoidae indet
Myriochele heeri
Lysippe sexcirrata
Owenia polaris
Paradoneis sp
Galathowenia oculata
Cossura sp
Myriochele sp
Nematoda
Apseudes sp
Spirorbinae indet
Autolytinae indet
-3
-2
-1
0
1
log(BPi)
B17-B13
Nemertea
Bathyarca glacialis
Nephtys incisa
Amphitrite groenlandica
Aglaophamus malmgreni
Cistenides hyperborea
Ctenodiscus crispatus
Ephesiella abyssorum
Quasimelita quadrispinosa
Edwardsia sp
Pherusa plumosa
Ampharete lindstroemi
Phyllodoce groenlandica
Amphiuridae indet
Ascelerichilus intermedius
Astarte crenata agg
Chirimia biceps
Cuspidaria obesa
Ampharete finmarchica
Aphroditoidea indet
Bathyarca frielei
Antalis entalis
Nephtys ciliata
Ophiocten sericeum
Euclymene droebachiensis
Lumbrineris mixochaeta
Praxiella gracilis
Maldanidae indet
Parougia caeca
Syllis cornuta agg
Phascolion strombi
Abyssoninoe hibernica
Praxillura longissima
Ennucula tenuis
Nephasoma procera
Eudorella emarginata
Retusa obtusa
Diastyloides biplicata
Diplocirrus hirsutus
Leitoscoloplos mammosus
Yoldiidae
Syllis sp
Lumbrinidae indet
Eteone longa
Aricidea quadrilobata
Ophelina abranchiata
Hippomedon sp
Syllis sp E
Nephtys juv
Leucon nasica
Paraonidae indet
Melinna sp
Eteone sp
Terebellides stroemii
Spiochaetopterus typicus
Aphelochaeta marioni
Brachydiastylis resima
Terebratulina retusa
Harpinia antennaria
Tanaidacea
Rhodine gracilor
Caulleriella sp A
Ampelisca sp
Calathura norvegica
Clymenura polaris
Maldane sarsi
Gnathia elongata
Leptognathia gracilis
Spiophanes bombyx
Pellecepoda
Prionospio cirrifera
Paraonides sp
Gnathia maxillaris
Calathura sp
Adontorhina juv
Chaetozone setosa
Clymenura sp
Mediomastus fragilis
Levinsenia gracilis
Ostracoda
Dialychone spp
Diastylis lucifera
Haploops setosa
Aricidea catherinae
Exogone sp
Cirrophorus eliasoni
Spiophanes kroyeri
Heteromastus filiformis
Harpinia sp
Haploops tubicola
Pholoidae indet
Lysippe sexcirrata
Owenia polaris
Paradoneis sp
Galathowenia oculata
Cossura sp
Myriochele heeri
Myriochele sp
Aricidea suecica
Apseudes sp
Spirorbinae indet
Autolytinae indet
Nematoda
-3
-2
-1
0
1
log(BPi)

Figure S3 | Non-metric two-dimensional (nMDS) representations of Bray–Curtis similarity matrices from Solan et al. (2020) based on (*a*) square root transformed abundance and (*b*) untransformed biomass for stations B13–B17 (indicated by colour) in 2017 (circles) and stations B13–B17 and Xs in 2018 (triangles). The classification of faunal assemblages in the Barents Sea demonstrates a distinct separation between the northern and southern stations.

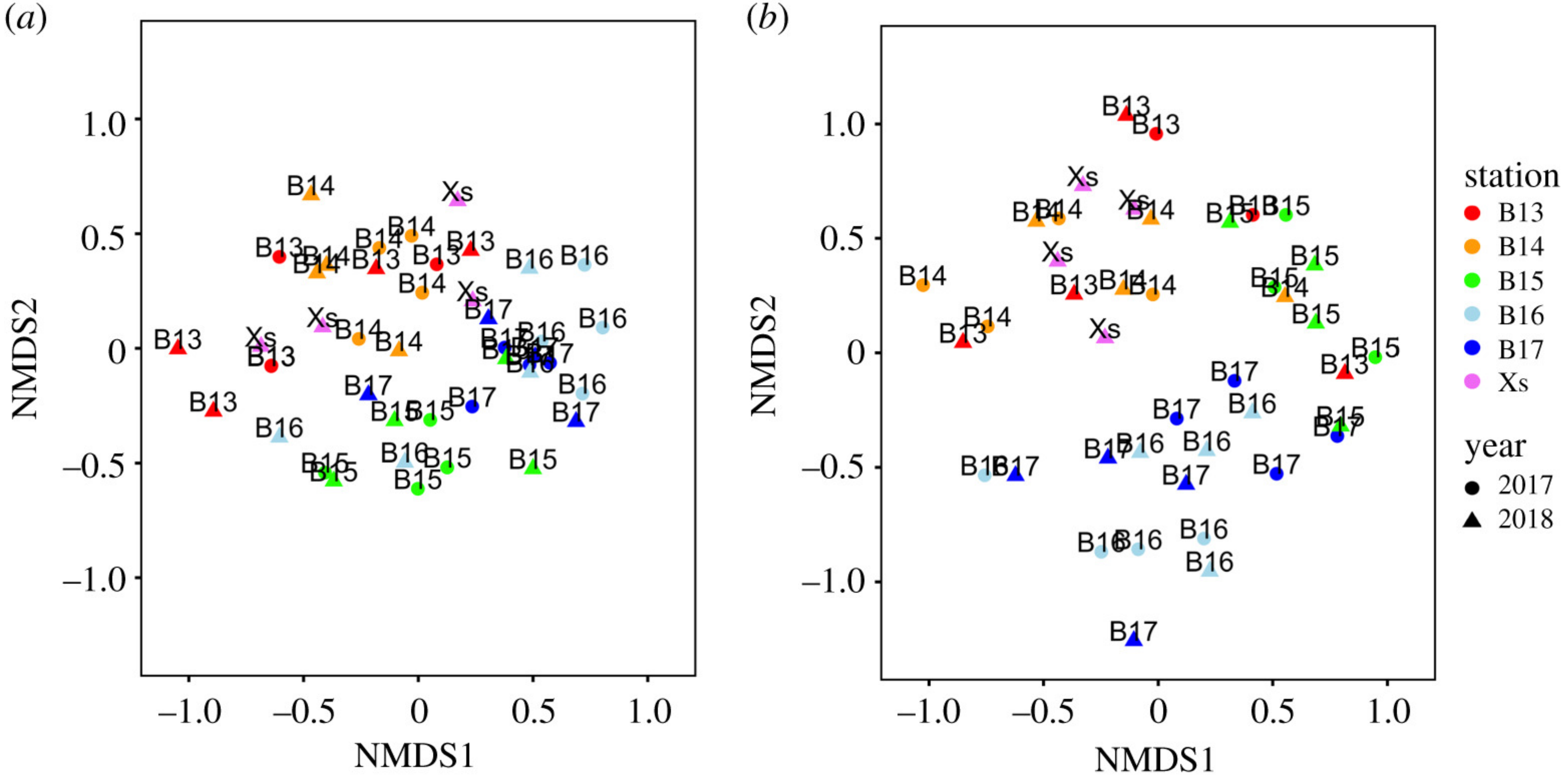

Figure S4 | The (a) weighted network and (b) frequency of interactions between taxa (abundance > 1 across all stations; n = 69) estimated from 466 correlations in biomass across all station deployments (n = 24). Only positive (green) and negative (red) correlations ≳ 1.5 s.d. from the mean score (0.0397) are displayed in S3(a), where colour saturation and width of connections correspond to the absolute correlation score and scale relative to the strongest correlation score (1.0000). Cutoff (0.55) relates to the correlation value for maximum connection thickness, with all connections higher than the cutoff sharing the same thickness. Maximum relates to the maximum correlation score shown. Clustering is computed using the Fruchterman-Reingold algorithm, where nodes repel each other equally before connections create an attraction force based on the correlation score, irrespective of its sign (Fruchterman & Reingold, 1991). In S3(b), correlations mainly cluster within 1.5 standard deviations of the mean score (grey dashed horizontal bar), with more positive (green dashed horizontal bar) than negative correlations (red dashed horizontal bar) occurring outside this range.

Figure S4(a)

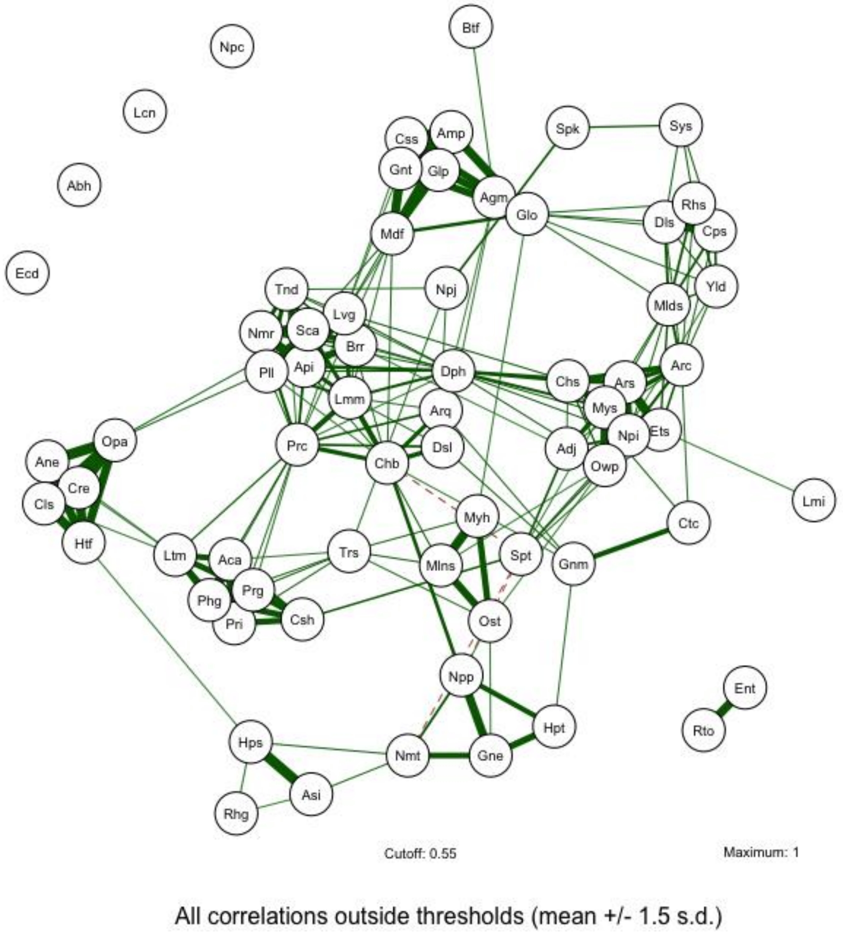

Figure S4(b)

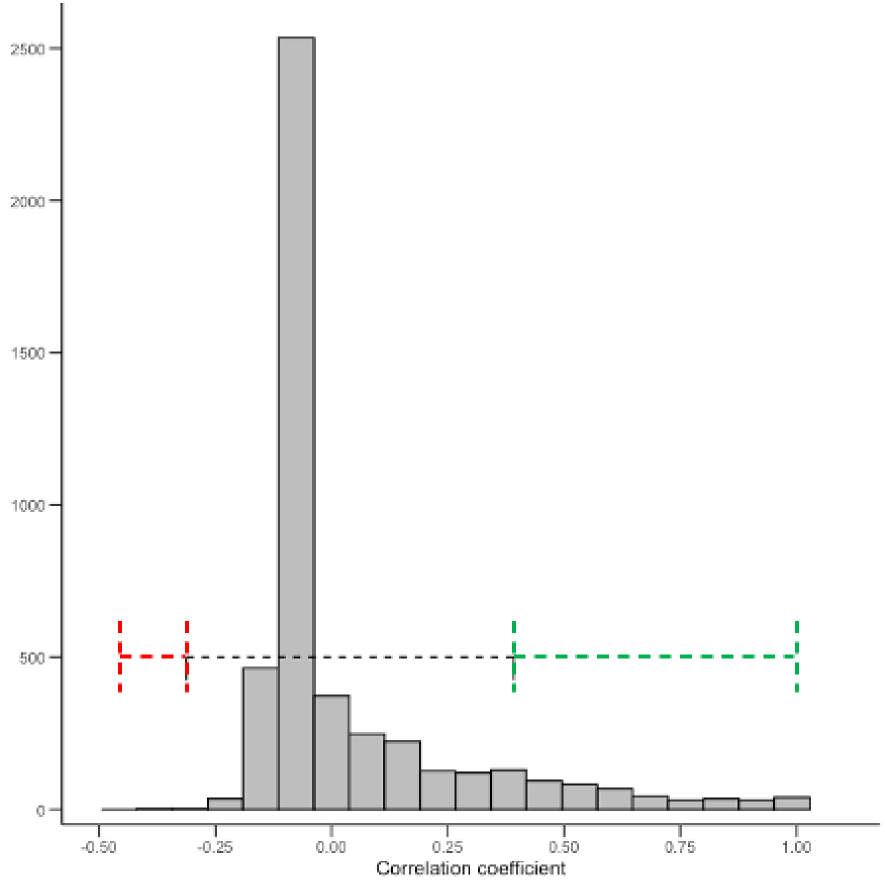

Table S3 | List of sediment-dwelling invertebrate species with (a) positive (n = 460) and (b) negative correlations (n = 6) ≳1.5 standard deviations from the mean coefficient value (Figure S4).

Table S3 (a)

Sp1	Sp2	correlation coefficient
Adontorhina juv	*Aricidea catherinae*	0.44490047
Adontorhina juv	*Aricidea suecica*	0.65324341
Adontorhina juv	*Brachydiastylis resima*	0.39901141
Adontorhina juv	*Chaetozone setosa*	0.44925732
Adontorhina juv	*Eteone sp*	0.55942251
Adontorhina juv	*Myriochele sp*	0.65208596
Adontorhina juv	*Nephtys incisa*	0.58154394
Adontorhina juv	*Owenia polaris*	0.55005358
Adontorhina juv	*Spiochaetopterus typicus*	0.58280577
Aglaophamus malmgreni	*Aricidea quadrilobata*	0.39549028
Aglaophamus malmgreni	*Bathyarca frielei*	0.469328
Aglaophamus malmgreni	*Diastylis lucifera*	0.41354182
Ampeliscidae	*Cossura sp*	0.98666973
Ampeliscidae	*Galathowenia oculata*	0.86343044
Ampeliscidae	*Glyphanostomum pallescens*	0.99130832
Ampeliscidae	*Gnathiidae*	0.99130832
Ampeliscidae	*Mediomastus fragilis*	0.8340697
Antalis entalis	*Cirrophorus eliasoni*	0.97355357
Antalis entalis	*Clymenura sp*	0.97355357
Antalis entalis	*Heteromastus filiformis*	0.85989273
Antalis entalis	*Leitoscoloplos mammosus*	0.40048705
Antalis entalis	*Ophelina abranchiata*	0.94265891
Aphroditoidea indet	*Brachydiastylis resima*	0.72447193
Aphroditoidea indet	*Chirimia biceps*	0.46499525
Aphroditoidea indet	*Diplocirrus hirsutus*	0.63553438
Aphroditoidea indet	*Levinsenia gracillis*	0.73574765
Aphroditoidea indet	*Lumbrineris mixochaeta*	0.66114928
Aphroditoidea indet	*Nemertea*	0.89322531
Aphroditoidea indet	*Pellecepoda*	0.91713348
Aphroditoidea indet	*Prionospio cirrifera*	0.59222719
Aphroditoidea indet	*Syllis cornuta agg*	0.90642424
Aphroditoidea indet	*Tanaidacea*	0.76019463
Aricidea catherinae	*Adontorhina juv*	0.44490047
Aricidea catherinae	*Aricidea suecica*	0.6769014
Aricidea catherinae	*Capitella sp*	0.48163601
Aricidea catherinae	*Chaetozone setosa*	0.71877728
Aricidea catherinae	*Ctenodiscus crispatus*	0.46770247
Aricidea catherinae	*Dialychone spp*	0.45636358
Aricidea catherinae	*Eteone sp*	0.58034329
Aricidea catherinae	*Maldane sarsi*	0.60755555
Aricidea catherinae	*Myriochele sp*	0.67995672
Aricidea catherinae	*Nephtys incisa*	0.5988667
Aricidea catherinae	*Owenia polaris*	0.57041645
Aricidea catherinae	*Rhodine sp*	0.48163601
Aricidea catherinae	*Yoldiidae*	0.49961056
Aricidea quadrilobata	*Aglaophamus malmgreni*	0.39549028

Aricidea quadrilobata	*Chirimia biceps*	0.66620934
Aricidea quadrilobata	*Diastylis lucifera*	0.98483334
Aricidea quadrilobata	*Gnathia maxillaris*	0.48409644
Aricidea quadrilobata	*Lumbrineris mixochaeta*	0.44188879
Aricidea quadrilobata	*Nephtys juv*	0.45557401
Aricidea quadrilobata	*Prionospio cirrifera*	0.58527962
Aricidea suecica	*Adontorhina juv*	0.65324341
Aricidea suecica	*Aricidea catherinae*	0.6769014
Aricidea suecica	*Chaetozone setosa*	0.67021999
Aricidea suecica	*Diplocirrus hirsutus*	0.61278768
Aricidea suecica	*Eteone sp*	0.88886812
Aricidea suecica	*Maldane sarsi*	0.50733476
Aricidea suecica	*Myriochele sp*	0.99905437
Aricidea suecica	*Nephtys incisa*	0.91474314
Aricidea suecica	*Owenia polaris*	0.87618827
Ascelerichilus intermedius	*Haploops setosa*	0.93926974
Ascelerichilus intermedius	*Nematoda*	0.4907254
Ascelerichilus intermedius	*Rhodine gracilor*	0.40810919
Astarte crenata agg	*Cistenides hyperborea*	0.67955818
Astarte crenata agg	*Leitoscoloplos mammosus*	0.74198895
Astarte crenata agg	*Paraonidae indet*	0.9265528
Astarte crenata agg	*Phyllodoce groenlandica*	0.92831992
Astarte crenata agg	*Praxiella gracilis*	0.9237353
Astarte crenata agg	*Prionospio cirrifera*	0.54399854
Astarte crenata agg	*Terebellides stroemii*	0.42223172
Bathyarca frielei	*Aglaophamus malmgreni*	0.469328
Brachydiastylis resima	*Adontorhina juv*	0.39901141
Brachydiastylis resima	*Aphroditoidea indet*	0.72447193
Brachydiastylis resima	*Diplocirrus hirsutus*	0.40640716
Brachydiastylis resima	*Levinsenia gracillis*	0.63265662
Brachydiastylis resima	*Lumbrineris mixochaeta*	0.48315825
Brachydiastylis resima	*Nemertea*	0.61063316
Brachydiastylis resima	*Pellecepoda*	0.64208916
Brachydiastylis resima	*Syllis cornuta agg*	0.72280314
Brachydiastylis resima	*Tanaidacea*	0.57472834
Capitella sp	*Aricidea catherinae*	0.48163601
Capitella sp	*Dialychone spp*	0.97632349
Capitella sp	*Galathowenia oculata*	0.49883342
Capitella sp	*Maldane sarsi*	0.5929407
Capitella sp	*Rhodine sp*	1
Capitella sp	*Syllis sp*	0.52925332
Capitella sp	*Yoldiidae*	0.58450611
Chaetozone setosa	*Adontorhina juv*	0.44925732
Chaetozone setosa	*Aricidea catherinae*	0.71877728
Chaetozone setosa	*Aricidea suecica*	0.67021999
Chaetozone setosa	*Ctenodiscus crispatus*	0.51119839
Chaetozone setosa	*Diplocirrus hirsutus*	0.62733184
Chaetozone setosa	*Eteone sp*	0.57329039
Chaetozone setosa	*Levinsenia gracillis*	0.53210197

Chaetozone setosa	*Myriochele sp*	0.67338372
Chaetozone setosa	*Nephtys incisa*	0.59387801
Chaetozone setosa	*Owenia polaris*	0.5633835
Chirimia biceps	*Aphroditoidea indet*	0.46499525
Chirimia biceps	*Aricidea quadrilobata*	0.66620934
Chirimia biceps	*Diastylis lucifera*	0.69050057
Chirimia biceps	*Gnathia maxillaris*	0.41149345
Chirimia biceps	*Levinsenia gracillis*	0.47536286
Chirimia biceps	*Lumbrineris mixochaeta*	0.71715938
Chirimia biceps	*Mediomastus fragilis*	0.49030427
Chirimia biceps	*Nephasoma procera*	0.64166231
Chirimia biceps	*Pellecepoda*	0.4213375
Chirimia biceps	*Prionospio cirrifera*	0.68226427
Chirimia biceps	*Syllis cornuta agg*	0.49724918
Cirrophorus eliasoni	*Antalis entalis*	0.97355357
Cirrophorus eliasoni	*Clymenura sp*	1
Cirrophorus eliasoni	*Heteromastus filiformis*	0.89060905
Cirrophorus eliasoni	*Leitoscoloplos mammosus*	0.41997644
Cirrophorus eliasoni	*Ophelina abranchiata*	0.97358866
Cistenides hyperborea	*Astarte crenata agg*	0.67955818
Cistenides hyperborea	*Leitoscoloplos mammosus*	0.58478494
Cistenides hyperborea	*Paraonidae indet*	0.75382893
Cistenides hyperborea	*Phyllodoce groenlandica*	0.75633975
Cistenides hyperborea	*Praxiella gracilis*	0.7596907
Cistenides hyperborea	*Spiochaetopterus typicus*	0.5747216
Clymenura sp	*Antalis entalis*	0.97355357
Clymenura sp	*Cirrophorus eliasoni*	1
Clymenura sp	*Heteromastus filiformis*	0.89060905
Clymenura sp	*Leitoscoloplos mammosus*	0.41997644
Clymenura sp	*Ophelina abranchiata*	0.97358866
Cossura sp	*Ampeliscidae*	0.98666973
Cossura sp	*Galathowenia oculata*	0.79342606
Cossura sp	*Glyphanostomum pallescens*	0.99586853
Cossura sp	*Gnathiidae*	0.99586853
Cossura sp	*Levinsenia gracillis*	0.41513559
Cossura sp	*Mediomastus fragilis*	0.84383606
Ctenodiscus crispatus	*Aricidea catherinae*	0.46770247
Ctenodiscus crispatus	*Chaetozone setosa*	0.51119839
Ctenodiscus crispatus	*Gnathia maxillaris*	0.70901712
Dialychone spp	*Aricidea catherinae*	0.45636358
Dialychone spp	*Capitella sp*	0.97632349
Dialychone spp	*Galathowenia oculata*	0.47612562
Dialychone spp	*Maldane sarsi*	0.6038821
Dialychone spp	*Rhodine sp*	0.97632349
Dialychone spp	*Syllis sp*	0.5083799
Dialychone spp	*Yoldiidae*	0.54771716
Diastylis lucifera	*Aglaophamus malmgreni*	0.41354182
Diastylis lucifera	*Aricidea quadrilobata*	0.98483334
Diastylis lucifera	*Chirimia biceps*	0.69050057
Diastylis lucifera	*Gnathia maxillaris*	0.50102349
Diastylis lucifera	*Lumbrineris mixochaeta*	0.46425117

Diastylis lucifera	*Prionospio cirrifera*	0.59222719
Diplocirrus hirsutus	*Aphroditoidea indet*	0.63553438
Diplocirrus hirsutus	*Aricidea suecica*	0.61278768
Diplocirrus hirsutus	*Brachydiastylis resima*	0.40640716
Diplocirrus hirsutus	*Chaetozone setosa*	0.62733184
Diplocirrus hirsutus	*Eteone sp*	0.51552062
Diplocirrus hirsutus	*Levinsenia gracillis*	0.5508677
Diplocirrus hirsutus	*Lumbrineris mixochaeta*	0.6124198
Diplocirrus hirsutus	*Myriochele sp*	0.60827772
Diplocirrus hirsutus	*Nemertea*	0.52030658
Diplocirrus hirsutus	*Nephtys incisa*	0.51641954
Diplocirrus hirsutus	*Owenia polaris*	0.50644825
Diplocirrus hirsutus	*Pellecepoda*	0.55385365
Diplocirrus hirsutus	*Prionospio cirrifera*	0.41980384
Diplocirrus hirsutus	*Syllis cornuta agg*	0.55221566
Diplocirrus hirsutus	*Tanaidacea*	0.43468053
Ennucula tenuis	*Retusa obtusa*	0.86549354
Eteone sp	*Adontorhina juv*	0.55942251
Eteone sp	*Aricidea catherinae*	0.58034329
Eteone sp	*Aricidea suecica*	0.88886812
Eteone sp	*Chaetozone setosa*	0.57329039
Eteone sp	*Diplocirrus hirsutus*	0.51552062
Eteone sp	*Lumbrinidae indet*	0.41561756
Eteone sp	*Maldane sarsi*	0.57262329
Eteone sp	*Myriochele sp*	0.89060905
Eteone sp	*Nephtys incisa*	0.97156647
Eteone sp	*Owenia polaris*	0.77202731
Eteone sp	*Spiochaetopterus typicus*	0.45335255
Galathowenia oculata	*Ampeliscidae*	0.86343044
Galathowenia oculata	*Capitella sp*	0.49883342
Galathowenia oculata	*Cossura sp*	0.79342606
Galathowenia oculata	*Dialychone spp*	0.47612562
Galathowenia oculata	*Glyphanostomum pallescens*	0.8001156
Galathowenia oculata	*Gnathiidae*	0.8001156
Galathowenia oculata	*Maldane sarsi*	0.48204579
Galathowenia oculata	*Mediomastus fragilis*	0.64157121
Galathowenia oculata	*Myriochele heeri*	0.42732954
Galathowenia oculata	*Rhodine sp*	0.49883342
Galathowenia oculata	*Yoldiidae*	0.46768293
Glyphanostomum pallescens	*Ampeliscidae*	0.99130832
Glyphanostomum pallescens	*Cossura sp*	0.99586853
Glyphanostomum pallescens	*Galathowenia oculata*	0.8001156
Glyphanostomum pallescens	*Gnathiidae*	1
Glyphanostomum pallescens	*Levinsenia gracillis*	0.40389708
Glyphanostomum pallescens	*Mediomastus fragilis*	0.84726528
Gnathia elongata	*Haploops tubicola*	0.77724314
Gnathia elongata	*Nematoda*	0.7381138

Gnathia elongata	*Nephasoma procera*	0.85534119
Gnathia elongata	*Ostracoda*	0.4197823
Gnathia maxillaris	*Aricidea quadrilobata*	0.48409644
Gnathia maxillaris	*Chirimia biceps*	0.41149345
Gnathia maxillaris	*Ctenodiscus crispatus*	0.70901712
Gnathia maxillaris	*Diastylis lucifera*	0.50102349
Gnathia maxillaris	*Haploops tubicola*	0.46032232
Gnathiidae	*Ampeliscidae*	0.99130832
Gnathiidae	*Cossura sp*	0.99586853
Gnathiidae	*Galathowenia oculata*	0.8001156
Gnathiidae	*Glyphanostomum pallescens*	1
Gnathiidae	*Levinsenia gracillis*	0.40389708
Gnathiidae	*Mediomastus fragilis*	0.84726528
Haploops setosa	*Ascelerichilus intermedius*	0.93926974
Haploops setosa	*Heteromastus filiformis*	0.41561756
Haploops setosa	*Nematoda*	0.51004797
Haploops setosa	*Rhodine gracilor*	0.45613448
Haploops tubicola	*Gnathia elongata*	0.77724314
Haploops tubicola	*Gnathia maxillaris*	0.46032232
Haploops tubicola	*Nephasoma procera*	0.71054791
Heteromastus filiformis	*Antalis entalis*	0.85989273
Heteromastus filiformis	*Cirrophorus eliasoni*	0.89060905
Heteromastus filiformis	*Clymenura sp*	0.89060905
Heteromastus filiformis	*Haploops setosa*	0.41561756
Heteromastus filiformis	*Ophelina abranchiata*	0.86028619
Leitoscoloplos mammosus	*Antalis entalis*	0.40048705
Leitoscoloplos mammosus	*Astarte crenata agg*	0.74198895
Leitoscoloplos mammosus	*Cirrophorus eliasoni*	0.41997644
Leitoscoloplos mammosus	*Cistenides hyperborea*	0.58478494
Leitoscoloplos mammosus	*Clymenura sp*	0.41997644
Leitoscoloplos mammosus	*Paraonidae indet*	0.73384883
Leitoscoloplos mammosus	*Phyllodoce groenlandica*	0.73836851
Leitoscoloplos mammosus	*Praxiella gracilis*	0.74208101
Leitoscoloplos mammosus	*Prionospio cirrifera*	0.55845291
Levinsenia gracillis	*Aphroditoidea indet*	0.73574765
Levinsenia gracillis	*Brachydiastylis resima*	0.63265662
Levinsenia gracillis	*Chaetozone setosa*	0.53210197
Levinsenia gracillis	*Chirimia biceps*	0.47536286
Levinsenia gracillis	*Cossura sp*	0.41513559
Levinsenia gracillis	*Diplocirrus hirsutus*	0.5508677
Levinsenia gracillis	*Glyphanostomum pallescens*	0.40389708
Levinsenia gracillis	*Gnathiidae*	0.40389708
Levinsenia gracillis	*Lumbrineris mixochaeta*	0.65467046
Levinsenia gracillis	*Mediomastus fragilis*	0.50073088

Levinsenia gracillis	*Nemertea*	0.60909048
Levinsenia gracillis	*Ophelina abranchiata*	0.4812985
Levinsenia gracillis	*Pellecepoda*	0.81887741
Levinsenia gracillis	*Prionospio cirrifera*	0.42803324
Levinsenia gracillis	*Syllis cornuta agg*	0.8511783
Levinsenia gracillis	*Tanaidacea*	0.49865456
Lumbrineris mixochaeta	*Aphroditoidea indet*	0.66114928
Lumbrineris mixochaeta	*Aricidea quadrilobata*	0.44188879
Lumbrineris mixochaeta	*Brachydiastylis resima*	0.48315825
Lumbrineris mixochaeta	*Chirimia biceps*	0.71715938
Lumbrineris mixochaeta	*Diastylis lucifera*	0.46425117
Lumbrineris mixochaeta	*Diplocirrus hirsutus*	0.6124198
Lumbrineris mixochaeta	*Levinsenia gracillis*	0.65467046
Lumbrineris mixochaeta	*Mediomastus fragilis*	0.43705009
Lumbrineris mixochaeta	*Melinna sp*	0.52232368
Lumbrineris mixochaeta	*Nemertea*	0.56053065
Lumbrineris mixochaeta	*Pellecepoda*	0.60908411
Lumbrineris mixochaeta	*Prionospio cirrifera*	0.71553221
Lumbrineris mixochaeta	*Syllis cornuta agg*	0.65647107
Lumbrineris mixochaeta	*Tanaidacea*	0.42237943
Lumbrinidae indet	*Eteone sp*	0.41561756
Maldane sarsi	*Aricidea catherinae*	0.60755555
Maldane sarsi	*Aricidea suecica*	0.50733476
Maldane sarsi	*Capitella sp*	0.5929407
Maldane sarsi	*Dialychone spp*	0.6038821
Maldane sarsi	*Eteone sp*	0.57262329
Maldane sarsi	*Galathowenia oculata*	0.48204579
Maldane sarsi	*Myriochele sp*	0.50295976
Maldane sarsi	*Nephtys incisa*	0.5183831
Maldane sarsi	*Rhodine sp*	0.5929407
Maldane sarsi	*Yoldiidae*	0.56493878
Mediomastus fragilis	*Ampeliscidae*	0.8340697
Mediomastus fragilis	*Chirimia biceps*	0.49030427
Mediomastus fragilis	*Cossura sp*	0.84383606
Mediomastus fragilis	*Galathowenia oculata*	0.64157121
Mediomastus fragilis	*Glyphanostomum pallescens*	0.84726528
Mediomastus fragilis	*Gnathiidae*	0.84726528
Mediomastus fragilis	*Levinsenia gracillis*	0.50073088
Mediomastus fragilis	*Lumbrineris mixochaeta*	0.43705009
Mediomastus fragilis	*Prionospio cirrifera*	0.43581994
Mediomastus fragilis	*Syllis cornuta agg*	0.49156799
Melinna sp	*Lumbrineris mixochaeta*	0.52232368
Melinna sp	*Myriochele heeri*	0.88642033
Melinna sp	*Ostracoda*	0.8153058
Melinna sp	*Owenia polaris*	0.40387286
Melinna sp	*Terebellides stroemii*	0.52312745
Myriochele heeri	*Galathowenia oculata*	0.42732954
Myriochele heeri	*Melinna sp*	0.88642033
Myriochele heeri	*Ostracoda*	0.76351236
Myriochele heeri	*Terebellides stroemii*	0.50444763
Myriochele sp	*Adontorhina juv*	0.65208596

Myriochele sp	*Aricidea catherinae*	0.67995672
Myriochele sp	*Aricidea suecica*	0.99905437
Myriochele sp	*Chaetozone setosa*	0.67338372
Myriochele sp	*Diplocirrus hirsutus*	0.60827772
Myriochele sp	*Eteone sp*	0.89060905
Myriochele sp	*Maldane sarsi*	0.50295976
Myriochele sp	*Nephtys incisa*	0.91702917
Myriochele sp	*Owenia polaris*	0.8779646
Myriochele sp	*Spiochaetopterus typicus*	0.39150531
Nematoda	*Ascelerichilus intermedius*	0.4907254
Nematoda	*Gnathia elongata*	0.7381138
Nematoda	*Haploops setosa*	0.51004797
Nematoda	*Nephasoma procera*	0.59547824
Nemertea	*Aphroditoidea indet*	0.89322531
Nemertea	*Brachydiastylis resima*	0.61063316
Nemertea	*Diplocirrus hirsutus*	0.52030658
Nemertea	*Levinsenia gracillis*	0.60909048
Nemertea	*Lumbrineris mixochaeta*	0.56053065
Nemertea	*Pellecepoda*	0.80909715
Nemertea	*Prionospio cirrifera*	0.61816686
Nemertea	*Syllis cornuta agg*	0.79353467
Nemertea	*Tanaidacea*	0.66782913
Nephasoma procera	*Chirimia biceps*	0.64166231
Nephasoma procera	*Gnathia elongata*	0.85534119
Nephasoma procera	*Haploops tubicola*	0.71054791
Nephasoma procera	*Nematoda*	0.59547824
Nephasoma procera	*Ostracoda*	0.4245698
Nephtys incisa	*Adontorhina juv*	0.58154394
Nephtys incisa	*Aricidea catherinae*	0.5988667
Nephtys incisa	*Aricidea suecica*	0.91474314
Nephtys incisa	*Chaetozone setosa*	0.59387801
Nephtys incisa	*Diplocirrus hirsutus*	0.51641954
Nephtys incisa	*Eteone sp*	0.97156647
Nephtys incisa	*Maldane sarsi*	0.5183831
Nephtys incisa	*Myriochele sp*	0.91702917
Nephtys incisa	*Owenia polaris*	0.8598009
Nephtys incisa	*Spiochaetopterus typicus*	0.52230307
Nephtys juv	*Aricidea quadrilobata*	0.45557401
Nephtys juv	*Spiophanes kroyeri*	0.5831241
Nephtys juv	*Tanaidacea*	0.47348154
Nephtys juv	*Terebellides stroemii*	0.45302377
Ophelina abranchiata	*Antalis entalis*	0.94265891
Ophelina abranchiata	*Cirrophorus eliasoni*	0.97358866
Ophelina abranchiata	*Clymenura sp*	0.97358866
Ophelina abranchiata	*Heteromastus filiformis*	0.86028619
Ophelina abranchiata	*Levinsenia gracillis*	0.4812985
Ophelina abranchiata	*Pellecepoda*	0.48723277
Ostracoda	*Gnathia elongata*	0.4197823
Ostracoda	*Melinna sp*	0.8153058
Ostracoda	*Myriochele heeri*	0.76351236
Ostracoda	*Nephasoma procera*	0.4245698

Ostracoda	*Owenia polaris*	0.39946195
Ostracoda	*Terebellides stroemii*	0.45937557
Owenia polaris	*Adontorhina juv*	0.55005358
Owenia polaris	*Aricidea catherinae*	0.57041645
Owenia polaris	*Aricidea suecica*	0.87618827
Owenia polaris	*Chaetozone setosa*	0.5633835
Owenia polaris	*Diplocirrus hirsutus*	0.50644825
Owenia polaris	*Eteone sp*	0.77202731
Owenia polaris	*Melinna sp*	0.40387286
Owenia polaris	*Myriochele sp*	0.8779646
Owenia polaris	*Nephtys incisa*	0.8598009
Owenia polaris	*Ostracoda*	0.39946195
Owenia polaris	*Spiochaetopterus typicus*	0.5572169
Owenia polaris	*Yoldiidae*	0.407717
Paraonidae indet	*Astarte crenata agg*	0.9265528
Paraonidae indet	*Cistenides hyperborea*	0.75382893
Paraonidae indet	*Leitoscoloplos mammosus*	0.73384883
Paraonidae indet	*Phyllodoce groenlandica*	0.99907649
Paraonidae indet	*Praxiella gracilis*	0.99592993
Paraonidae indet	*Prionospio cirrifera*	0.45446842
Paraonidae indet	*Terebellides stroemii*	0.49207222
Pellecepoda	*Aphroditoidea indet*	0.91713348
Pellecepoda	*Brachydiastylis resima*	0.64208916
Pellecepoda	*Chirimia biceps*	0.4213375
Pellecepoda	*Diplocirrus hirsutus*	0.55385365
Pellecepoda	*Levinsenia gracillis*	0.81887741
Pellecepoda	*Lumbrineris mixochaeta*	0.60908411
Pellecepoda	*Nemertea*	0.80909715
Pellecepoda	*Ophelina abranchiata*	0.48723277
Pellecepoda	*Prionospio cirrifera*	0.51452728
Pellecepoda	*Syllis cornuta agg*	0.82144589
Pellecepoda	*Tanaidacea*	0.67715614
Phyllodoce groenlandica	*Astarte crenata agg*	0.92831992
Phyllodoce groenlandica	*Cistenides hyperborea*	0.75633975
Phyllodoce groenlandica	*Leitoscoloplos mammosus*	0.73836851
Phyllodoce groenlandica	*Paraonidae indet*	0.99907649
Phyllodoce groenlandica	*Praxiella gracilis*	0.99695853
Phyllodoce groenlandica	*Prionospio cirrifera*	0.45723194
Phyllodoce groenlandica	*Terebellides stroemii*	0.49325334
Praxiella gracilis	*Astarte crenata agg*	0.9237353
Praxiella gracilis	*Cistenides hyperborea*	0.7596907
Praxiella gracilis	*Leitoscoloplos mammosus*	0.74208101
Praxiella gracilis	*Paraonidae indet*	0.99592993
Praxiella gracilis	*Phyllodoce groenlandica*	0.99695853
Praxiella gracilis	*Prionospio cirrifera*	0.45134386
Praxiella gracilis	*Terebellides stroemii*	0.53829116
Prionospio cirrifera	*Aphroditoidea indet*	0.59222719
Prionospio cirrifera	*Aricidea quadrilobata*	0.58527962
Prionospio cirrifera	*Astarte crenata agg*	0.54399854
Prionospio cirrifera	*Chirimia biceps*	0.68226427
Prionospio cirrifera	*Diastylis lucifera*	0.59222719

Prionospio cirrifera	*Diplocirrus hirsutus*	0.41980384
Prionospio cirrifera	*Leitoscoloplos mammosus*	0.55845291
Prionospio cirrifera	*Levinsenia gracillis*	0.42803324
Prionospio cirrifera	*Lumbrineris mixochaeta*	0.71553221
Prionospio cirrifera	*Mediomastus fragilis*	0.43581994
Prionospio cirrifera	*Nemertea*	0.61816686
Prionospio cirrifera	*Paraonidae indet*	0.45446842
Prionospio cirrifera	*Pellecepoda*	0.51452728
Prionospio cirrifera	*Phyllodoce groenlandica*	0.45723194
Prionospio cirrifera	*Praxiella gracilis*	0.45134386
Prionospio cirrifera	*Syllis cornuta agg*	0.51328323
Prionospio cirrifera	*Tanaidacea*	0.44373949
Retusa obtusa	*Ennucula tenuis*	0.86549354
Rhodine gracilor	*Ascelerichilus intermedius*	0.40810919
Rhodine gracilor	*Haploops setosa*	0.45613448
Rhodine sp	*Aricidea catherinae*	0.48163601
Rhodine sp	*Capitella sp*	1
Rhodine sp	*Dialychone spp*	0.97632349
Rhodine sp	*Galathowenia oculata*	0.49883342
Rhodine sp	*Maldane sarsi*	0.5929407
Rhodine sp	*Syllis sp*	0.52925332
Rhodine sp	*Yoldiidae*	0.58450611
Spiochaetopterus typicus	*Adontorhina juv*	0.58280577
Spiochaetopterus typicus	*Cistenides hyperborea*	0.5747216
Spiochaetopterus typicus	*Eteone sp*	0.45335255
Spiochaetopterus typicus	*Myriochele sp*	0.39150531
Spiochaetopterus typicus	*Nephtys incisa*	0.52230307
Spiochaetopterus typicus	*Owenia polaris*	0.5572169
Spiophanes kroyeri	*Nephtys juv*	0.5831241
Spiophanes kroyeri	*Syllis sp*	0.56566236
Syllis cornuta agg	*Aphroditoidea indet*	0.90642424
Syllis cornuta agg	*Brachydiastylis resima*	0.72280314
Syllis cornuta agg	*Chirimia biceps*	0.49724918
Syllis cornuta agg	*Diplocirrus hirsutus*	0.55221566
Syllis cornuta agg	*Levinsenia gracillis*	0.8511783
Syllis cornuta agg	*Lumbrineris mixochaeta*	0.65647107
Syllis cornuta agg	*Mediomastus fragilis*	0.49156799
Syllis cornuta agg	*Nemertea*	0.79353467
Syllis cornuta agg	*Pellecepoda*	0.82144589
Syllis cornuta agg	*Prionospio cirrifera*	0.51328323
Syllis cornuta agg	*Tanaidacea*	0.67258505
Syllis sp	*Capitella sp*	0.52925332
Syllis sp	*Dialychone spp*	0.5083799
Syllis sp	*Rhodine sp*	0.52925332
Syllis sp	*Spiophanes kroyeri*	0.56566236
Tanaidacea	*Aphroditoidea indet*	0.76019463
Tanaidacea	*Brachydiastylis resima*	0.57472834
Tanaidacea	*Diplocirrus hirsutus*	0.43468053
Tanaidacea	*Levinsenia gracillis*	0.49865456
Tanaidacea	*Lumbrineris mixochaeta*	0.42237943
Tanaidacea	*Nemertea*	0.66782913

Tanaidacea	*Nephtys juv*	0.47348154
Tanaidacea	*Pellecepoda*	0.67715614
Tanaidacea	*Prionospio cirrifera*	0.44373949
Tanaidacea	*Syllis cornuta agg*	0.67258505
Terebellides stroemii	*Astarte crenata agg*	0.42223172
Terebellides stroemii	*Melinna sp*	0.52312745
Terebellides stroemii	*Myriochele heeri*	0.50444763
Terebellides stroemii	*Nephtys juv*	0.45302377
Terebellides stroemii	*Ostracoda*	0.45937557
Terebellides stroemii	*Paraonidae indet*	0.49207222
Terebellides stroemii	*Phyllodoce groenlandica*	0.49325334
Terebellides stroemii	*Praxiella gracilis*	0.53829116
Yoldiidae	*Aricidea catherinae*	0.49961056
Yoldiidae	*Capitella sp*	0.58450611
Yoldiidae	*Dialychone spp*	0.54771716
Yoldiidae	*Galathowenia oculata*	0.46768293
Yoldiidae	*Maldane sarsi*	0.56493878
Yoldiidae	*Owenia polaris*	0.407717
Yoldiidae	*Rhodine sp*	0.58450611

Table S3 (b)

**Sp1**	**Sp2**	**correlation coefficient**
Chirimia biceps	*Spiochaetopterus typicus*	-0.3968943
Nematoda	*Spiochaetopterus typicus*	-0.3529203
Nephasoma procera	*Spiochaetopterus typicus*	-0.3138353
Spiochaetopterus typicus	*Chirimia biceps*	-0.3968943
Spiochaetopterus typicus	*Nematoda*	-0.3529203
Spiochaetopterus typicus	*Nephasoma procera*	-0.3138353

Figure S5 | Summary of the steps performed to determine the number of compensating species for each iterative extinction. As a species is extirpated, biomass is lost (B_{lost}) and a compensating species is randomly selected based on their compensation probability (CPSim). This species will increase in abundance (A_{start}), either up to pre-extinction median abundance (A_{med}) or to the equivalent biomass that the extirpated species filled. Multiple species can compensate should the original compensator's starting abundance not reach the pre-extinction median abundance. Compensation stops once lost biomass is fully replaced or all surviving species reach their assemblage median abundance. This process ensures that compensatory responses are finite and reflects the likely carrying capacity.

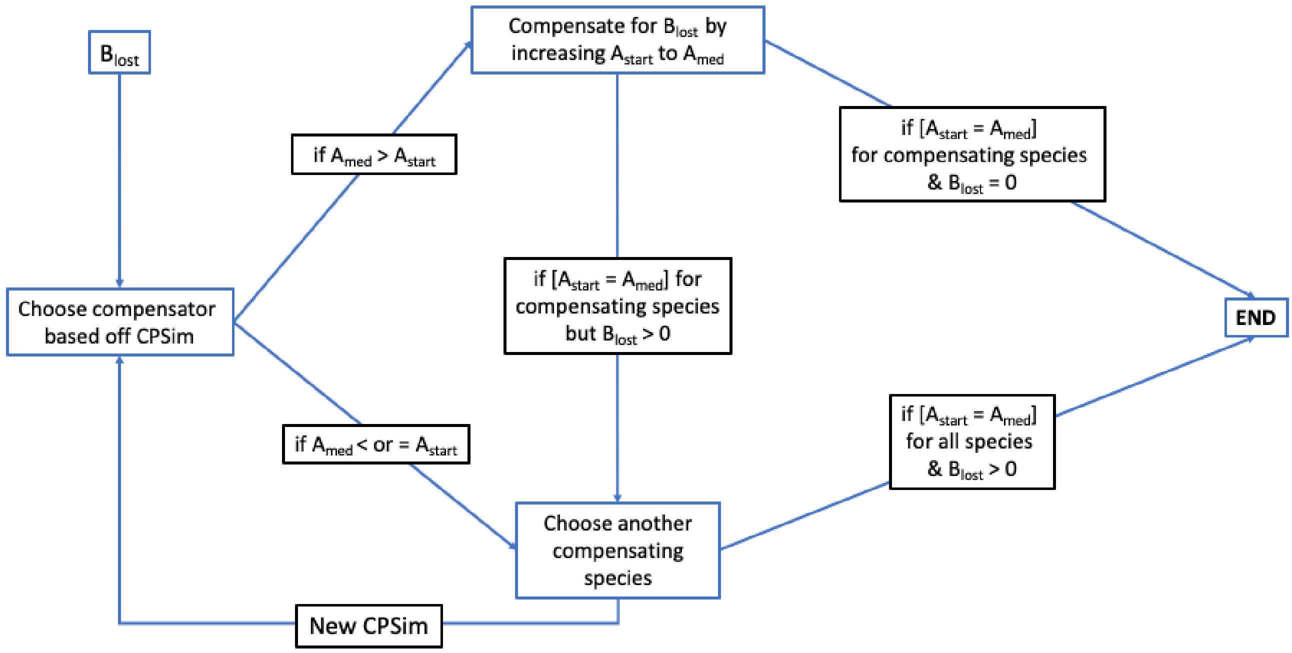

Table S4 | Summary of backward stepwise selection of best (bold) Generalised Additive Model (GAM) predicting bioturbation potential (BP_C) as a function of species richness (Nsp_active) and extinction scenario (Scenario), informed by the Akaike Information Criteria (AIC), the deviance explained (R^2) and smoothing criterion. Smoothing terms are carried out with the default thin plate splines (tp).

Model ID	Response Variable	Terms	n	AIC	R^2	Maximum Likelihood Smoothing Criterion
**3**	**BPc**	**s(Nsp_active, by = Scenario, bs = "tp") + Scenario**	**346414**	**4325256.88**	**0.85**	**2162766.09**
1		s(Nsp_active, by = Scenario, bs = "tp") + s(Nsp_active, bs = "tp") + Scenario	346414	4325280.06	0.85	2162781.08
5		s(Nsp_active, by = Scenario, bs = "tp")	346414	4366866.64	0.83	2183687.29
2		s(Nsp_active, by = Scenario, bs = "tp") + s(Nsp_active, bs = "tp")	346414	4366868.03	0.83	2183729.08
4		s(Nsp_active, bs = "tp") + Scenario	346414	4416076.03	0.80	2208056.58
6		s(Nsp_active, bs = "tp")	346414	4633264.20	0.63	2316653.17
7		Scenario	346414	4842915.22	0.33	2421450.61
8		1	346414	4981343.42	0.00	2490669.71

Figure S6 | Comparison of partial estimated slopes of linear models (dashed lines) with the same structure of the best GAM estimated with fREML (solid lines) that predict log(BP_c) as a response to species richness and extinction scenario (colour: B17-B13 | B17-B16 | B16-B15 | B15-Xs | Xs-B14| B14-B13).

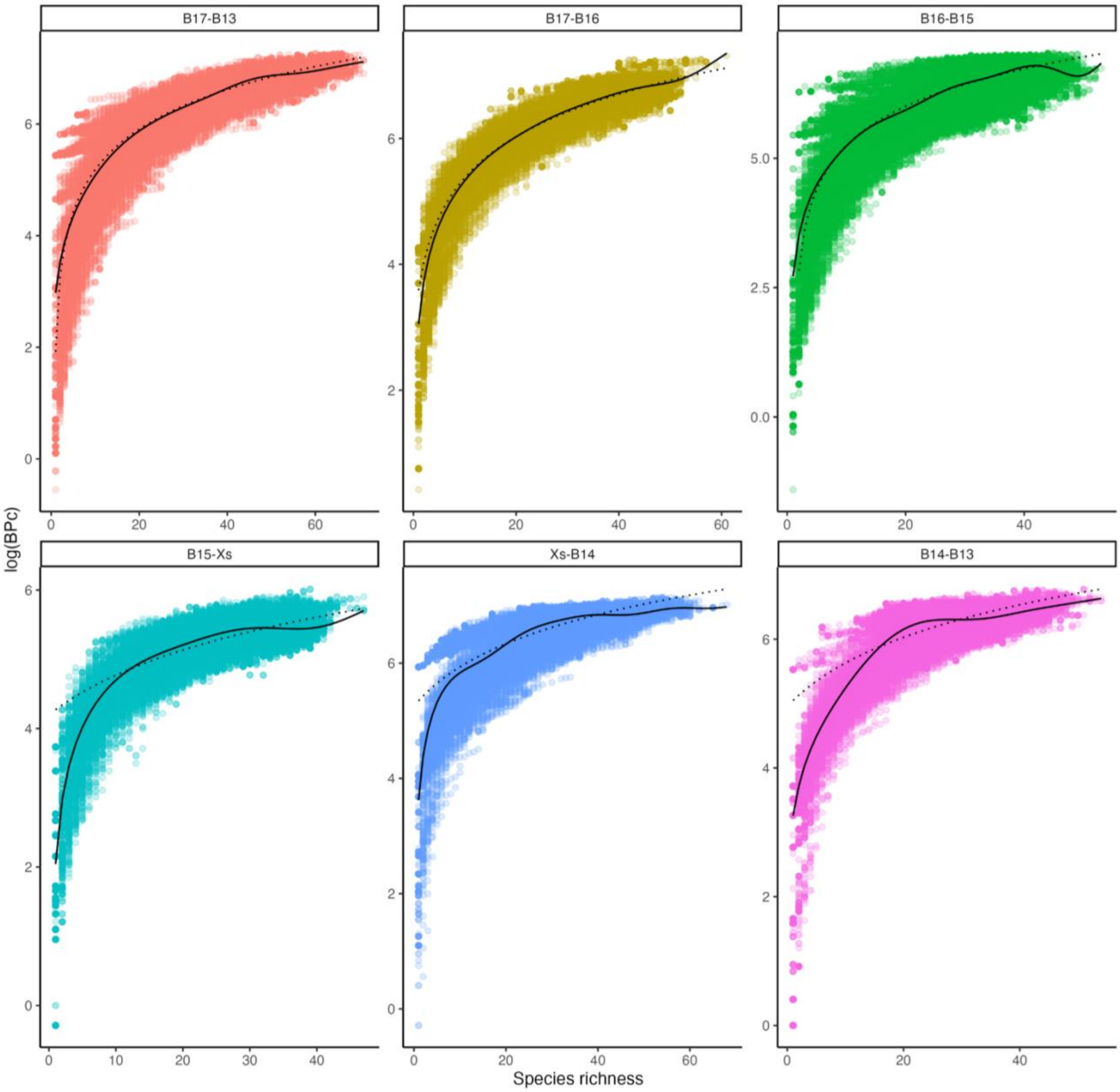

Table S5 | Summary of linear models used to investigate changes in the climate vulnerability (log extinction probability) of all species going extinct (ALL), species still present within the community (PRESENT), species going extinct as a result of climate vulnerabilities (CLIMATE) and species going extinct as a result of species co-dependencies (CO-EXT) as species richness declined. Pearson's correlation coefficient and significance (two-tailed) is stated.

Model	Extinction scenario	n	Pearson's r	R^2	p
log(extinction probability)$_{ALL}$ ~ species richness	B17-B16	346414	0.19	0.04	< 0.001
	B16-B15	346414	0.03	< 0.01	< 0.001
	B15-Xs	346414	0.13	0.02	< 0.001
	Xs-B14	346414	0.13	0.02	< 0.001
	B14-B13	346414	0.01	< 0.01	< 0.001
	B17-B13	346414	0.11	0.01	< 0.001
log(extinction probability)$_{PRESENT}$ ~ species richness	B17-B16	346414	0.37	0.14	< 0.001
	B16-B15	346414	-0.03	< 0.01	< 0.001
	B15-Xs	346414	0.11	0.01	< 0.001
	Xs-B14	346414	0.18	0.03	< 0.001
	B14-B13	346414	0.54	0.29	< 0.001
	B17-B13	346414	0.29	0.09	< 0.001
log(extinction probability)$_{CLIMATE}$ ~ species richness	B17-B16	346414	0.21	0.05	< 0.001
	B16-B15	346414	0.06	< 0.01	< 0.001
	B15-Xs	346414	0.14	0.02	< 0.001
	Xs-B14	346414	0.15	0.02	< 0.001
	B14-B13	346414	0.11	0.01	< 0.001
	B17-B13	346414	0.13	0.02	< 0.001
log(extinction probability)$_{CO\text{-}EXT}$ ~ species richness	B17-B16	346414	0.19	0.04	< 0.001
	B16-B15	346414	0.02	< 0.01	0.006
	B15-Xs	346414	-0.14	0.02	< 0.001
	Xs-B14	346414	0.1	0.01	< 0.001
	B14-B13	346414	0.26	0.07	< 0.001
	B17-B13	346414	0.16	0.03	< 0.001

Supplementary Code S1-S8

Each supplementary code chunk referenced in the manuscript can also be found in R markdown file “Williams_et_al_CoExt_ChAOS_SupplementaryCode.Rmd” available at https://doi.org/10.6084/m9.figshare.28062653

```
sessionInfo()

## R version 4.3.2 (2023-10-31)
## Platform: aarch64-apple-darwin20 (64-bit)
## Running under: macOS Sonoma 14.2
##
## Matrix products: default
## BLAS:   /Library/Frameworks/R.framework/Versions/4.3-
arm64/Resources/lib/libRblas.0.dylib
## LAPACK: /Library/Frameworks/R.framework/Versions/4.3-
arm64/Resources/lib/libRlapack.dylib;  LAPACK version 3.11.0
##
## locale:
## [1] en_US.UTF-8/en_US.UTF-8/en_US.UTF-8/C/en_US.UTF-8/en_US.UTF-8
##
## time zone: Europe/London
## tzcode source: internal
##
## attached base packages:
## [1] stats     graphics  grDevices utils     datasets  methods   base
##
## loaded via a namespace (and not attached):
##  [1] compiler_4.3.2    fastmap_1.2.0     cli_3.6.3         formatR_1.14
##  [5] tools_4.3.2       htmltools_0.5.8.1 rstudioapi_0.16.0 yaml_2.3.10
##  [9] rmarkdown_2.28    knitr_1.48        xfun_0.47         digest_0.6.37
## [13] rlang_1.1.4       evaluate_1.0.0
```

This script performs the supplementary code required for the extinction and compensation model on local (station B17 to station B16 | station B16 to station B15 | station B15 to station Xs | station Xs to station B14 | station B14 to B13) and transect-wide (station B17 to station B13) macrobenthic biodiversity data in the Barents Sea (Arctic), collected during the 2018 summer research expedition aboard the RRS James Clark Ross (Solan et al., 2020). Each supplementary code chunk is referenced in the manuscript and explained in detail below.

Loading of packages

```
packages <- c("tidyverse", "Hmisc", "qgraph", "rio", "patchwork",
    "MetBrewer", "ggpmisc", "mgcv")
# Install packages not yet installed
installed_packages <- packages %in% rownames(installed.packages())
if (any(installed_packages == FALSE)) {
    install.packages(packages[!installed_packages], repos = "http://cran.us.r-
project.org")
}

# Packages loading
invisible(lapply(packages, library, character.only = TRUE))

## ── Attaching core tidyverse packages ──────────────────────── tidyverse
2.0.0 ──
```

```
## ✓ dplyr     1.1.4     ✓ readr     2.1.5
## ✓ forcats   1.0.0     ✓ stringr   1.5.1
## ✓ ggplot2   3.5.1     ✓ tibble    3.2.1
## ✓ lubridate 1.9.3     ✓ tidyr     1.3.1
## ✓ purrr     1.0.2
## ── Conflicts ──────────────────────────────────────────
tidyverse_conflicts() ──
## ✗ dplyr::filter() masks stats::filter()
## ✗ dplyr::lag()    masks stats::lag()
## i Use the conflicted package (<http://conflicted.r-lib.org/>) to force all
conflicts to become errors

## Warning: package 'Hmisc' was built under R version 4.3.3

##
## Attaching package: 'Hmisc'
##
## The following objects are masked from 'package:dplyr':
##
##     src, summarize
##
## The following objects are masked from 'package:base':
##
##     format.pval, units

## Warning: package 'rio' was built under R version 4.3.3

## Warning: package 'patchwork' was built under R version 4.3.3

## Warning: package 'ggpmisc' was built under R version 4.3.3

## Loading required package: ggpp

## Warning: package 'ggpp' was built under R version 4.3.3

## Registered S3 methods overwritten by 'ggpp':
##   method                  from
##   heightDetails.titleGrob ggplot2
##   widthDetails.titleGrob  ggplot2
##
## Attaching package: 'ggpp'
##
## The following object is masked from 'package:ggplot2':
##
##     annotate
##
## Loading required package: nlme

## Warning: package 'nlme' was built under R version 4.3.3

##
## Attaching package: 'nlme'
##
## The following object is masked from 'package:dplyr':
##
##     collapse
```

```
##
## This is mgcv 1.9-1. For overview type 'help("mgcv-package")'.
```

Initial load of data and data wrangling

```
ChAOS_macrofauna_2018 <- read.csv("ChAOS_2018_macrofauna_data.csv")  # or
file.choose()

## Stations, Habitats and Scenarios ####
ChAOS_stations_2018 <- data.frame(Station = c("B17", "B16", "B15",
    "Xs", "B14", "B13", "B17"), Habitat = c("Arctic", "Arctic",
    "Boreal", "Boreal", "Boreal", "Boreal", "Boreal"), Scenario = c("B17-B16",
    "B16-B15", "B15-Xs", "Xs-B14", "B14-B13", "None", "B17-B13"))

ChAOS_macrofauna_stations_2018 <- merge(ChAOS_macrofauna_2018,
    ChAOS_stations_2018, by = "Station", all.x = T)

## mean and total abundance and biomass for 2018 ####
ChAOS_2018_mean <- ChAOS_macrofauna_stations_2018 %>%
    group_by(ScientificName_accepted, Station) %>%
    summarise(Habitat, Scenario, Mi = Mi, Ri = Ri, Bi = mean(Biomass),
        Ai = mean(Abundance), Btot = sum(Biomass), Atot = sum(Abundance)) %>%
    tibble()

## Warning: Returning more (or less) than 1 row per `summarise()` group was
deprecated in
## dplyr 1.1.0.
## ℹ Please use `reframe()` instead.
## ℹ When switching from `summarise()` to `reframe()`, remember that
`reframe()`
##   always returns an ungrouped data frame and adjust accordingly.
## Call `lifecycle::last_lifecycle_warnings()` to see where this warning was
## generated.

## `summarise()` has grouped output by 'ScientificName_accepted', 'Station'.
You
## can override using the `.groups` argument.

ChAOS_2018_mean <- unique(ChAOS_2018_mean)

# Mean biomass per North (Arctic) and South (Boreal)
# habitats as defined by an already established gradient in
# the benthos [Solan et al. 2020] and environment (Polar
# Front; [Loeng, 1991])

ChAOS_2018_mean <- ChAOS_2018_mean %>%
    group_by(ScientificName_accepted, Habitat) %>%
    summarise(Station, Scenario, Mi = Mi, Ri = Ri, Bi, Ai, Btot,
        Atot, Bind_Habitat = sum(Btot)/sum(Atot))

## Warning: Returning more (or less) than 1 row per `summarise()` group was
deprecated in
## dplyr 1.1.0.
## ℹ Please use `reframe()` instead.
## ℹ When switching from `summarise()` to `reframe()`, remember that
`reframe()`
##   always returns an ungrouped data frame and adjust accordingly.
```

```
## Call `lifecycle::last_lifecycle_warnings()` to see where this warning was
## generated.

## `summarise()` has grouped output by 'ScientificName_accepted', 'Habitat'.
You
## can override using the `.groups` argument.

ChAOS_2018_mean[is.na(ChAOS_2018_mean)] <- 0

# Scenario-based Bind ####

ChAOS_2018_mean$Bind_Scenario = ChAOS_2018_mean$Bind_Habitat

# For the B16-B15 and B17-B13 scenarios, this crosses the
# Polar Front (i.e. moving from 'Arctic' to 'boreal').
# Hence, Bind is slightly different. If species are found
# at both stations, then the allocated Bind is based off
# boreal Bind. If species are only found at B16/B17, then
# the allocated Bind is based off the Arctic.
ChAOS_2018_Species <-
as.character(unique(ChAOS_2018_mean$ScientificName_accepted))

for (i in ChAOS_2018_Species) {
    if (ChAOS_2018_mean[which(ChAOS_2018_mean$Station == "B16" &
        ChAOS_2018_mean$ScientificName_accepted == i), ]$Bi >
        0 && ChAOS_2018_mean[which(ChAOS_2018_mean$Station ==
        "B15" & ChAOS_2018_mean$ScientificName_accepted == i),
        ]$Bi == 0) {
        ChAOS_2018_mean[which(ChAOS_2018_mean$Scenario == "B16-B15" &
            ChAOS_2018_mean$ScientificName_accepted == i), ]$Bind_Scenario <-
unique(ChAOS_2018_mean[which(ChAOS_2018_mean$Habitat ==
            "Arctic" & ChAOS_2018_mean$ScientificName_accepted ==
            i), ]$Bind_Habitat)
    } else {
        ChAOS_2018_mean[which(ChAOS_2018_mean$Scenario == "B16-B15" &
            ChAOS_2018_mean$ScientificName_accepted == i), ]$Bind_Scenario <-
unique(ChAOS_2018_mean[which(ChAOS_2018_mean$Habitat ==
            "Boreal" & ChAOS_2018_mean$ScientificName_accepted ==
            i), ]$Bind_Habitat)
    }
}

for (i in ChAOS_2018_Species) {
    if (ChAOS_2018_mean[which(ChAOS_2018_mean$Station == "B17" &
        ChAOS_2018_mean$Scenario == "B17-B13" &
ChAOS_2018_mean$ScientificName_accepted ==
        i), ]$Bi > 0 && ChAOS_2018_mean[which(ChAOS_2018_mean$Station ==
        "B13" & ChAOS_2018_mean$ScientificName_accepted == i),
        ]$Bi == 0) {
        ChAOS_2018_mean[which(ChAOS_2018_mean$Scenario == "B17-B13" &
            ChAOS_2018_mean$ScientificName_accepted == i), ]$Bind_Scenario <-
unique(ChAOS_2018_mean[which(ChAOS_2018_mean$Habitat ==
            "Arctic" & ChAOS_2018_mean$ScientificName_accepted ==
            i), ]$Bind_Habitat)
    } else {
        ChAOS_2018_mean[which(ChAOS_2018_mean$Scenario == "B17-B13" &
            ChAOS_2018_mean$ScientificName_accepted == i), ]$Bind_Scenario <-
```

```
unique(ChAOS_2018_mean[which(ChAOS_2018_mean$Habitat ==
            "Boreal" & ChAOS_2018_mean$ScientificName_accepted ==
            i), ]$Bind_Habitat)
    }
}


rm(ChAOS_stations_2018)
```

Code S1: Vulnerability of species

Ranked vulnerabilities to each step in the climatic-driven environmental transition (B17-B16 | B16-B15 | B15-Xs | Xs-B14 | B14-B13 | B17-B13), calculated from the percentage differences in macrofaunal biomass between the pre-extinction community (northernmost station, e.g. B17) and the reference post-extinction community (southernmost station, e.g. B16) for all taxa in the regional species pool

```
# Calculate percentage differences in biomass between
# starting station and reference station #### B17- B16
E16 <- (ChAOS_2018_mean[which(ChAOS_2018_mean$Station == "B16"),
    ]$Bi - ChAOS_2018_mean[which(ChAOS_2018_mean$Station == "B17" &
    ChAOS_2018_mean$Scenario == "B17-B16"),
]$Bi)/ChAOS_2018_mean[which(ChAOS_2018_mean$Station ==
    "B17" & ChAOS_2018_mean$Scenario == "B17-B16"), ]$Bi

# B16- B15
E15 <- (ChAOS_2018_mean[which(ChAOS_2018_mean$Station == "B15"),
    ]$Bi - ChAOS_2018_mean[which(ChAOS_2018_mean$Station == "B16" &
    ChAOS_2018_mean$Scenario == "B16-B15"),
]$Bi)/ChAOS_2018_mean[which(ChAOS_2018_mean$Station ==
    "B16" & ChAOS_2018_mean$Scenario == "B16-B15"), ]$Bi

# B15- Xs
EXs <- (ChAOS_2018_mean[which(ChAOS_2018_mean$Station == "Xs"),
    ]$Bi - ChAOS_2018_mean[which(ChAOS_2018_mean$Station == "B15"),
    ]$Bi)/ChAOS_2018_mean[which(ChAOS_2018_mean$Station == "B15"),
    ]$Bi

# Xs- B14
E14 <- (ChAOS_2018_mean[which(ChAOS_2018_mean$Station == "B14"),
    ]$Bi - ChAOS_2018_mean[which(ChAOS_2018_mean$Station == "Xs"),
    ]$Bi)/ChAOS_2018_mean[which(ChAOS_2018_mean$Station == "Xs"),
    ]$Bi

# B14- B13
E13 <- (ChAOS_2018_mean[which(ChAOS_2018_mean$Station == "B13"),
    ]$Bi - ChAOS_2018_mean[which(ChAOS_2018_mean$Station == "B14"),
    ]$Bi)/ChAOS_2018_mean[which(ChAOS_2018_mean$Station == "B14"),
    ]$Bi

# B17 - B13
Eall <- (ChAOS_2018_mean[which(ChAOS_2018_mean$Station == "B13"),
    ]$Bi - ChAOS_2018_mean[which(ChAOS_2018_mean$Station == "B17" &
    ChAOS_2018_mean$Scenario == "B17-B13"),
]$Bi)/ChAOS_2018_mean[which(ChAOS_2018_mean$Station ==
    "B17" & ChAOS_2018_mean$Scenario == "B17-B13"), ]$Bi
```

```
# row bind all the percentage differences ####
ChAOS_2018_Vulnerabilties_Biomass <- do.call(rbind, Map(data.frame,
    ScientificName_accepted = unique(ChAOS_2018_mean$ScientificName_accepted),
    E16 = E16, E15 = E15, EXs = EXs, E14 = E14, E13 = E13, Eall = Eall))
ChAOS_2018_Vulnerabilties_Biomass[is.na(ChAOS_2018_Vulnerabilties_Biomass)] <-
0

# now rank them from most to least vulnerable based on
# percetange differences #### B17 - B16
order.scores <- order(ChAOS_2018_Vulnerabilties_Biomass$E16)
ChAOS_2018_Vulnerabilties_Biomass <-
ChAOS_2018_Vulnerabilties_Biomass[order.scores,
    ]
ChAOS_2018_Vulnerabilties_Biomass$Biomass_E16rank <-
rank(ChAOS_2018_Vulnerabilties_Biomass$E16)
ChAOS_2018_Vulnerabilties_Biomass

# B16 - B15
order.scores <- order(ChAOS_2018_Vulnerabilties_Biomass$E15)
ChAOS_2018_Vulnerabilties_Biomass <-
ChAOS_2018_Vulnerabilties_Biomass[order.scores,
    ]
ChAOS_2018_Vulnerabilties_Biomass$Biomass_E15rank <-
rank(ChAOS_2018_Vulnerabilties_Biomass$E15)
ChAOS_2018_Vulnerabilties_Biomass

# B15 - Xs
order.scores <- order(ChAOS_2018_Vulnerabilties_Biomass$EXs)
ChAOS_2018_Vulnerabilties_Biomass <-
ChAOS_2018_Vulnerabilties_Biomass[order.scores,
    ]
ChAOS_2018_Vulnerabilties_Biomass$Biomass_EXsrank <-
rank(ChAOS_2018_Vulnerabilties_Biomass$EXs)
ChAOS_2018_Vulnerabilties_Biomass

# Xs - B14
order.scores <- order(ChAOS_2018_Vulnerabilties_Biomass$E14)
ChAOS_2018_Vulnerabilties_Biomass <-
ChAOS_2018_Vulnerabilties_Biomass[order.scores,
    ]
ChAOS_2018_Vulnerabilties_Biomass$Biomass_E14rank <-
rank(ChAOS_2018_Vulnerabilties_Biomass$E14)
ChAOS_2018_Vulnerabilties_Biomass

# B14 - B13
order.scores <- order(ChAOS_2018_Vulnerabilties_Biomass$E13)
ChAOS_2018_Vulnerabilties_Biomass <-
ChAOS_2018_Vulnerabilties_Biomass[order.scores,
    ]
ChAOS_2018_Vulnerabilties_Biomass$Biomass_E13rank <-
rank(ChAOS_2018_Vulnerabilties_Biomass$E13)
ChAOS_2018_Vulnerabilties_Biomass

# B17 - B13
order.scores <- order(ChAOS_2018_Vulnerabilties_Biomass$Eall)
```

```
ChAOS_2018_Vulnerabilties_Biomass <-
ChAOS_2018_Vulnerabilties_Biomass[order.scores,
    ]
ChAOS_2018_Vulnerabilties_Biomass$Biomass_Eallrank <-
rank(ChAOS_2018_Vulnerabilties_Biomass$Eall)
ChAOS_2018_Vulnerabilties_Biomass

# Finally, merge vulnerabilities with starting data ####
ChAOS_2018_mean$B_Vulnerability <- NA

ChAOS_2018_Species <-
as.character(unique(ChAOS_2018_mean$ScientificName_accepted))

for (i in ChAOS_2018_Species) {
    ChAOS_2018_mean[which(ChAOS_2018_mean$Scenario == "B17-B16" &
        ChAOS_2018_mean$ScientificName_accepted == i), ]$B_Vulnerability <-
ChAOS_2018_Vulnerabilties_Biomass[which(ChAOS_2018_Vulnerabilties_Biomass$Scien
tificName_accepted ==
        i), ]$Biomass_E16rank
    ChAOS_2018_mean[which(ChAOS_2018_mean$Scenario == "B16-B15" &
        ChAOS_2018_mean$ScientificName_accepted == i), ]$B_Vulnerability <-
ChAOS_2018_Vulnerabilties_Biomass[which(ChAOS_2018_Vulnerabilties_Biomass$Scien
tificName_accepted ==
        i), ]$Biomass_E15rank
    ChAOS_2018_mean[which(ChAOS_2018_mean$Scenario == "B15-Xs" &
        ChAOS_2018_mean$ScientificName_accepted == i), ]$B_Vulnerability <-
ChAOS_2018_Vulnerabilties_Biomass[which(ChAOS_2018_Vulnerabilties_Biomass$Scien
tificName_accepted ==
        i), ]$Biomass_EXsrank
    ChAOS_2018_mean[which(ChAOS_2018_mean$Scenario == "Xs-B14" &
        ChAOS_2018_mean$ScientificName_accepted == i), ]$B_Vulnerability <-
ChAOS_2018_Vulnerabilties_Biomass[which(ChAOS_2018_Vulnerabilties_Biomass$Scien
tificName_accepted ==
        i), ]$Biomass_E14rank
    ChAOS_2018_mean[which(ChAOS_2018_mean$Scenario == "B14-B13" &
        ChAOS_2018_mean$ScientificName_accepted == i), ]$B_Vulnerability <-
ChAOS_2018_Vulnerabilties_Biomass[which(ChAOS_2018_Vulnerabilties_Biomass$Scien
tificName_accepted ==
        i), ]$Biomass_E13rank
    ChAOS_2018_mean[which(ChAOS_2018_mean$Scenario == "B17-B13" &
        ChAOS_2018_mean$ScientificName_accepted == i), ]$B_Vulnerability <-
ChAOS_2018_Vulnerabilties_Biomass[which(ChAOS_2018_Vulnerabilties_Biomass$Scien
tificName_accepted ==
        i), ]$Biomass_Eallrank
}

rm(E16, E15, EXs, E14, E13, Eall)
rm(order.scores)
```

Code S2: Calculating the median abundance above and below the ecotone Polar Front (Solan et al. 2020; Loeng, 1991)

For B16-B15 & B17-B13 scenario, switch to Amed of boreal stations (B15-B13) as that is the abundance of the incoming species from the local pool (migrating northward with Atlantification)

```
## B17-B16 #####
ChAOS_Species_18_Compensation <- ChAOS_macrofauna_2018 %>%
    select(ScientificName_accepted, Mi, Ri, Year, Station, Replicate,
        Abundance, Biomass)
ChAOS_Species_18_Compensation$Abundance[ChAOS_Species_18_Compensation$Abundance
==
    0] <- NaN
ChAOS_Species_18_Compensation$Biomass[ChAOS_Species_18_Compensation$Biomass ==
    0] <- NaN
Species <- unique(ChAOS_Species_18_Compensation$ScientificName_accepted)


for (z in Species) {
    med_Abundance_B17_B16 <-
median(ChAOS_Species_18_Compensation[which(ChAOS_Species_18_Compensation$Scient
ificName_accepted ==
        z & c(ChAOS_Species_18_Compensation$Station == "B16" |
        ChAOS_Species_18_Compensation$Station == "B17")), ]$Abundance,
        na.rm = T)

    print(med_Abundance_B17_B16)


ChAOS_Species_18_Compensation[ChAOS_Species_18_Compensation$ScientificName_acce
pted ==
        z, "med_Abundance_B17_B16"] <- med_Abundance_B17_B16
}

# cant have Na!!
ChAOS_Species_18_Compensation$med_Abundance_B17_B16[is.na(ChAOS_Species_18_Comp
ensation$med_Abundance_B17_B16)] <- 0

## B15-B13 #####
for (z in Species) {
    med_Abundance_B15_B13 <-
median(ChAOS_Species_18_Compensation[which(ChAOS_Species_18_Compensation$Scient
ificName_accepted ==
        z & c(ChAOS_Species_18_Compensation$Station == "Xs" |
        ChAOS_Species_18_Compensation$Station == "B15" |
ChAOS_Species_18_Compensation$Station ==
        "B14" | ChAOS_Species_18_Compensation$Station == "B13")),
        ]$Abundance, na.rm = T)
    print(med_Abundance_B15_B13)


ChAOS_Species_18_Compensation[ChAOS_Species_18_Compensation$ScientificName_acce
pted ==
        z, "med_Abundance_B15_B13"] <- med_Abundance_B15_B13
}

# cant have Na!!
ChAOS_Species_18_Compensation$med_Abundance_B15_B13[is.na(ChAOS_Species_18_Comp
ensation$med_Abundance_B15_B13)] <- 0

## subsetting median abundances and adding to start dataset
## for model #####
```

```
ChAOS_2018_Compensation <- ChAOS_Species_18_Compensation %>%
    select(ScientificName_accepted, med_Abundance_B17_B16,
med_Abundance_B15_B13) %>%
    unique()

ChAOS_2018_mean$Amed <- NA

for (i in Species) {
    ChAOS_2018_mean[which(ChAOS_2018_mean$Scenario == "B17-B16" &
        ChAOS_2018_mean$ScientificName_accepted == i), ]$Amed <-
ChAOS_2018_Compensation[which(ChAOS_2018_Compensation$ScientificName_accepted
==
        i), ]$med_Abundance_B17_B16
    ChAOS_2018_mean[which(ChAOS_2018_mean$Scenario == "B16-B15" &
        ChAOS_2018_mean$ScientificName_accepted == i), ]$Amed <-
ChAOS_2018_Compensation[which(ChAOS_2018_Compensation$ScientificName_accepted
==
        i), ]$med_Abundance_B15_B13
    ChAOS_2018_mean[which(ChAOS_2018_mean$Scenario == "B15-Xs" &
        ChAOS_2018_mean$ScientificName_accepted == i), ]$Amed <-
ChAOS_2018_Compensation[which(ChAOS_2018_Compensation$ScientificName_accepted
==
        i), ]$med_Abundance_B15_B13
    ChAOS_2018_mean[which(ChAOS_2018_mean$Scenario == "Xs-B14" &
        ChAOS_2018_mean$ScientificName_accepted == i), ]$Amed <-
ChAOS_2018_Compensation[which(ChAOS_2018_Compensation$ScientificName_accepted
==
        i), ]$med_Abundance_B15_B13
    ChAOS_2018_mean[which(ChAOS_2018_mean$Scenario == "B14-B13" &
        ChAOS_2018_mean$ScientificName_accepted == i), ]$Amed <-
ChAOS_2018_Compensation[which(ChAOS_2018_Compensation$ScientificName_accepted
==
        i), ]$med_Abundance_B15_B13
    ChAOS_2018_mean[which(ChAOS_2018_mean$Scenario == "B17-B13" &
        ChAOS_2018_mean$ScientificName_accepted == i), ]$Amed <-
ChAOS_2018_Compensation[which(ChAOS_2018_Compensation$ScientificName_accepted
==
        i), ]$med_Abundance_B15_B13
}

write.csv(ChAOS_2018_mean, "ChAOS_2018_macrofauna_model_ready.csv")
```

Code S3: Calculating correlations for species co-extinctions and co-compensations

```
# Removing rare species from the correlation calculations
# ####

Sum_Abundance <- ChAOS_macrofauna_2018 %>%
    group_by(ScientificName_accepted) %>%
    summarise(suma = sum(Abundance/25))

Rare_Species <- subset(Sum_Abundance, subset = suma == 1, select =
ScientificName_accepted)

ChAOS_BiomassMatrix_18_no_rares <- ChAOS_macrofauna_2018 %>%
    select(ScientificName_accepted, Station, Replicate, Biomass) %>%
    pivot_wider(names_from = c(Station, Replicate), values_from = Biomass) %>%
```

```
    dplyr::filter(!ScientificName_accepted %in%
Rare_Species$ScientificName_accepted) %>%
    pivot_longer(-ScientificName_accepted) %>%
    pivot_wider(names_from = ScientificName_accepted, values_from = value)

# Biomass networks - All stations ####

All_stations_Community <- cor(ChAOS_BiomassMatrix_18_no_rares[,
    2:70], method = "pearson", use = "everything")
All_stations_Community <- cov2cor(All_stations_Community)

ChAOS_BiomassMatrix_18_allstations_CoOc <- data.frame(x =
rownames(All_stations_Community)[row(All_stations_Community)],
    y = colnames(All_stations_Community)[col(All_stations_Community)],
    correlation_coefficient = c(All_stations_Community))

# remove correlations between a species and itself
ChAOS_BiomassMatrix_18_allstations_CoOc <-
ChAOS_BiomassMatrix_18_allstations_CoOc[which(ChAOS_BiomassMatrix_18_allstation
s_CoOc$x !=
    ChAOS_BiomassMatrix_18_allstations_CoOc$y), ]

# Getting cut off lists #### finding out mean and standard
# deviations
qqnorm(ChAOS_BiomassMatrix_18_allstations_CoOc$correlation_coefficient)

mean(ChAOS_BiomassMatrix_18_allstations_CoOc$correlation_coefficient)
sd(ChAOS_BiomassMatrix_18_allstations_CoOc$correlation_coefficient)
uppercutoff <-
mean(ChAOS_BiomassMatrix_18_allstations_CoOc$correlation_coefficient) +
    (sd(ChAOS_BiomassMatrix_18_allstations_CoOc$correlation_coefficient) *
        1.5)
lowercutoff <-
mean(ChAOS_BiomassMatrix_18_allstations_CoOc$correlation_coefficient) -
    (sd(ChAOS_BiomassMatrix_18_allstations_CoOc$correlation_coefficient) *
        1.5)

ChAOS_BiomassMatrix_18_standarddev <- data.frame(mean =
mean(ChAOS_BiomassMatrix_18_allstations_CoOc$correlation_coefficient),
    sd = sd(ChAOS_BiomassMatrix_18_allstations_CoOc$correlation_coefficient *
        1.5))


# Cut off determination - 1.5 s.d. either side of mean
# correlation Co-occurring species
ChAOS_BiomassMatrix_18_allstations_CoOc_cutoff <-
subset(ChAOS_BiomassMatrix_18_allstations_CoOc,
    subset = correlation_coefficient >= uppercutoff)
ChAOS_BiomassMatrix_18_allstations_CoOc_cutoff$x <- gsub(" ",
    "_", ChAOS_BiomassMatrix_18_allstations_CoOc_cutoff$x)
ChAOS_BiomassMatrix_18_allstations_CoOc_cutoff$y <- gsub(" ",
    "_", ChAOS_BiomassMatrix_18_allstations_CoOc_cutoff$y)
ChAOS_BiomassMatrix_18_allstations_CoOc_cutoff$x <-
as.factor(ChAOS_BiomassMatrix_18_allstations_CoOc_cutoff$x)

write.csv(ChAOS_BiomassMatrix_18_allstations_CoOc_cutoff, file =
```

```
"ChAOS_2018_allstations_Co_Occurrence[based_off_Biomass].csv")

## Competing species
ChAOS_BiomassMatrix_18_allstations_Comp_cutoff <-
subset(ChAOS_BiomassMatrix_18_allstations_CoOc,
    subset = correlation_coefficient <= lowercutoff)
ChAOS_BiomassMatrix_18_allstations_Comp_cutoff$x <- gsub(" ",
    "_", ChAOS_BiomassMatrix_18_allstations_Comp_cutoff$x)
ChAOS_BiomassMatrix_18_allstations_Comp_cutoff$y <- gsub(" ",
    "_", ChAOS_BiomassMatrix_18_allstations_Comp_cutoff$y)
ChAOS_BiomassMatrix_18_allstations_Comp_cutoff$x <-
as.factor(ChAOS_BiomassMatrix_18_allstations_Comp_cutoff$x)

write.csv(ChAOS_BiomassMatrix_18_allstations_Comp_cutoff, file =
"ChAOS_2018_allstations_Competitors[based_off_Biomass].csv")

## subsetting Co-Occurrence files by species ####
ChAOS_BiomassMatrix_18_allstations_CoOc_cutoff_list_x <-
split(ChAOS_BiomassMatrix_18_allstations_CoOc_cutoff,
    with(ChAOS_BiomassMatrix_18_allstations_CoOc_cutoff, x),
    drop = TRUE)

export(ChAOS_BiomassMatrix_18_allstations_CoOc_cutoff_list_x,
    "ChAOS_2018_Coextinctionlist_Biomass.xlsx")

## subsetting Competitors files by species ####
ChAOS_BiomassMatrix_18_allstations_Comp_cutoff_list_x <-
split(ChAOS_BiomassMatrix_18_allstations_Comp_cutoff,
    with(ChAOS_BiomassMatrix_18_allstations_Comp_cutoff, x),
    drop = TRUE)

export(ChAOS_BiomassMatrix_18_allstations_Comp_cutoff_list_x,
    "ChAOS_2018_Competitorlist_Biomass.xlsx")
```

Code S4: Code for deciding co extinctions if (taken from williams_etal_CoExt_CHAOS_model.Rmd file)

```
CO_EXT <- TRUE

if (CO_EXT == T) {
    # Code S4: CO-EXTINCTION
    # Pick a second species to go extinct (species y), based on the highest
positive correlation with the species x
    # First, find species x co-extinction file from the list in the global
environment, and choose the highest co-occurring species
    # there are a few species that have no correlations - so check first
    if(!is.null(startCoextinctions[[species_x]])) {

    species_x_TopCoextinction <- top_n(startCoextinctions[[species_x]], 1,
correlation_coefficient)
    species_y <- as.character(species_x_TopCoextinction$y[1])

    # sometimes there will be multiple species that have the same correlation
(i.e. 0.978), so need to include all of them
    # in the ChAOS data, the top correlations of nine species are with more
than one species
```

```
    if(nrow(species_x_TopCoextinction) >1 ) {
      species_z <- as.character(species_x_TopCoextinction$y[2])
      }

    # additionally, one or both of the top correlating species (species y OR z) 
may have gone extinct in a previous round
    # in this case, need to re-choose species y & z
    # if ALL correlating species have already gone extinct (species y & z stay 
NA), have to just stop looking for co-extinctions
    # run a 21x for function with conditions, as the most correlations for a 
species is 22 (i+1)
    for (i in 1:21) {
      if(is.na(species_y) && is.na(species_z)) {break} else( # if species y has 
already gone extinct and there is no species z, then move down the correlation 
list of species x and re-choose species y
        if(start$EPSim[start$ScientificName_accepted == species_y] == 0 && 
is.na(species_z)) {
          nth_correlation <- 
nth(startCoextinctions[[species_x]]$correlation_coefficient, i+1)
species_x_TopCoextinctions <- 
startCoextinctions[[species_x]]$y[startCoextinctions[[species_x]]$correlation_c
oefficient == nth_correlation]
species_y <- as.character(species_x_TopCoextinctions[1])
} else ( # if species y has already gone extinct but species z has not, then 
species z becomes species y
         if(start$EPSim[start$ScientificName_accepted == species_y] == 0 && 
start$EPSim[start$ScientificName_accepted == species_z] != 0) {
         species_y <- species_z
         species_z <- NA
       } else ( # if species y and species z have already gone extinct, then 
move down the correlation list of species x and re-choose species y and z
       if(start$EPSim[start$ScientificName_accepted == species_y] == 0 && 
start$EPSim[start$ScientificName_accepted == species_z] == 0) {
         nth_correlation <- 
nth(startCoextinctions[[species_x]]$correlation_coefficient, i+1)
species_x_TopCoextinctions <- 
startCoextinctions[[species_x]]$y[startCoextinctions[[species_x]]$correlation_c
oefficient == nth_correlation]
species_y <- as.character(species_x_TopCoextinctions[1])
species_z <- as.character(species_x_TopCoextinctions[2])
       }
)
)
)
       }


    # Find which row species y is in the starting data
    # If species y is NA, Extinct 2 becomes an empty integer
    Extinct2 <- which(start == species_y, arr.ind=FALSE)
    Extinct3 <- which(start == species_z, arr.ind=FALSE)
}
}
    # How much biomass will be lost with the doomed species
    ## 1st species
    BiomassLost <- start[Extinct,"BiSim"]
```

```
    ## 2nd species (if there is one)
    BiomassLost2 <- if(is.null(startCoextinctions[[species_x]]) ||
is.na(species_y)) {
                              0} else{
                        start[Extinct2, "BiSim"]}

    ## 3rd species (if there is one)
    BiomassLost3 <- if(is.null(startCoextinctions[[species_x]]) ||
is.na(species_z)) {
                              0} else{
                        start[Extinct3, "BiSim"]}

    # total biomass lost
    BiomassLost <- BiomassLost + BiomassLost2 + BiomassLost3

    # Record ID of who has gone extinct at each iteration
    ## 1st species
    output[output$Simulation == sim_count & output$Nsp==sp_count,"ExtSp"] <-
as.character(start$ScientificName_accepted[Extinct])
    output[output$Simulation == sim_count &
output$Nsp==sp_count,"ExtSpExtProb"] <- start[Extinct, "ExtProb"]

    ## 2nd species (if there is one)
    if(!is.null(startCoextinctions[[species_x]]) && !is.na(species_y)) {
                          output[output$Simulation == sim_count &
output$Nsp==sp_count,"CoExtSp"] <-
as.character(start$ScientificName_accepted[Extinct2])
                          output[output$Simulation == sim_count &
output$Nsp==sp_count,"CoExtSpExtProb"] <- start[Extinct2, "ExtProb"]}

    ## 3nd species (if there is one)
    if(!is.null(startCoextinctions[[species_x]]) && !is.na(species_z)) {
                          output[output$Simulation == sim_count &
output$Nsp==sp_count,"CoExtSp2"] <-
as.character(start$ScientificName_accepted[Extinct3])
                          output[output$Simulation == sim_count &
output$Nsp==sp_count,"CoExtSp2ExtProb"] <- start[Extinct3, "ExtProb"]}
```

Code S5: Recalculating species probability to compensate based on negative correlations (taken from williams_etal_CoExt_CHAOS_model.Rmd file)

```
# Code S5 COMPENSATION Species going extinct (x, y and z)
# cannot compensate
start[Extinct, "CPSim"] <- 0
if (!is.na(species_y)) {
    start[Extinct2, "CPSim"] <- 0
}
if (!is.na(species_z)) {
    start[Extinct3, "CPSim"] <- 0
}

# Normalise for loss of species
start$CPSim <- start$CPSim/sum(start$CPSim)

# Create a temp dataframe of the species that correlate
```

```
# +vely with the now extinct species, (and another temp
# dataframe for those that correlate -vely)
Positivelist <- data.frame(species = startCoextinctions[[species_x]]$y,
    CoorV = startCoextinctions[[species_x]]$correlation_coefficient)
Negativelist <- data.frame(species = startCompetitors[[species_x]]$y,
    CoorV = startCompetitors[[species_x]]$correlation_coefficient)
Combinedlist <- rbind(Positivelist, Negativelist)

# Get a list of species names from the temp dataframes
Co_species <- as.character(Combinedlist$species)

# Calculate new compensation probabilities for correlating
# species
for (i in Co_species) {
    NewCPSim <- (start[which(start$ScientificName_accepted ==
        i), ]$CPSim - (Combinedlist[which(Combinedlist$species ==
        i), ]$CoorV * start[which(start$ScientificName_accepted ==
        i), ]$CPSim))
    start[start$ScientificName_accepted == i, "NewCPSim"] <- NewCPSim
}


# Normalise new compensation probabilities
start$NewCPSim <- start$NewCPSim/sum(start$NewCPSim)  # Normalise
```

Code S6: Calculating co-compensations if there is still biomass left over from the extinctions (taken from williams_etal_CoExt_CHAOS_model.Rmd file)

```
counter <- 1
# Code S6
# Compensation happens, with the amount of biomass corresponding to the
difference between starting and median abundance
# First, check there is still some lost biomass left to compensate (while
function). Not as important first time around but very important if there is
multiple compensating species. If there is not, then compensation is complete
(while function finishes).
# We use the lowest possible biomass found across the entire regional species
pool as the threshold.
# Secondly, have to check if there are still species available to compensate.
If there is not, then compensation is complete (while function finishes).
     while(BiomassLost > 0.000625 &&
           sum(start[which(start$CPSim !=0),]$AiSim) <
sum(start[which(start$CPSim !=0),]$Amed)) {

    # Next, check that median abundance is more than starting abundance -
otherwise, choose another compensating species.
    # Once we find an appropriate species, we break this loop.

     repeat{
       ifelse(start$Amed[Compensate] - start$AiSim[Compensate]<=0,
              Compensate <- which(cumsum(start$NewCPSim)>=runif(1))[1],
              break
       )}

    # now, we calculate the difference in abundance between the median and the
starting abundance for the compensating species
```

```
    Abundancediff <- start$Amed[Compensate] - start$AiSim[Compensate]

    # next, we calculate how much biomass this will increase by
    Biomassdiff <- start$Bind[Compensate]*Abundancediff

    # if it is higher than total biomass lost from the system, we adjust the
change in abundance to this maximum
    if(Biomassdiff - BiomassLost > 0) {
           Abundancediff <- BiomassLost/start$Bind[Compensate]}

    # next, we allow the compensating species to increase in number
corresponding to that difference and the amount of biomass there is
    start$AiSim[Compensate] <- start$AiSim[Compensate] + Abundancediff

    # next, we re-calculate how much biomass will increase by with the new
change in abundance
    Biomassdiff <- start$Bind[Compensate]*Abundancediff

    # then, we allow its biomass to increase by that amount
    start$BiSim[Compensate] <- start$BiSim[Compensate] + Biomassdiff

    # finally, we remove the compensated biomass from the lost biomass
    BiomassLost <- BiomassLost - Biomassdiff

    # now to record everything
    if (counter == 1) {

    # Record amount of compensation abundance - add output$CompRep ==
countvalue
    output[output$Simulation == sim_count & output$Nsp==sp_count &
output$CompRep == counter,"AbnComp"] <- Abundancediff
    # Record amount of compensation biomass
    output[output$Simulation == sim_count & output$Nsp==sp_count &
output$CompRep == counter,"BioComp"] <- Biomassdiff
    # Record the compensation species
    output[output$Simulation == sim_count & output$Nsp==sp_count &
output$CompRep == counter,"CompSp"] <-
as.character(start$ScientificName_accepted[Compensate])

    # Add 1 to counter
    counter <- counter + 1

    } else {
      # add row into output table
      output<- output %>%
      add_row(Simulation = sim_count, # that does not change
              Nsp=sp_count, # that does not change
              CompRep = counter, # that should now be at least 2 (the 1 would
have been your normal line allocation)
              Nsp_active = Nsp_active, # does not change
              ExtSp = as.character(start$ScientificName_accepted[Extinct]), #
that doesn’t change
              ExtSpExtProb = start[Extinct, "ExtProb"], # that doesn't change
              CoExtSp = if(!is.null(startCoextinctions[[species_x]]) &&
!is.na(species_y)) {
```

```
as.character(start$ScientificName_accepted[Extinct2])}, # that doesn't change
              CoExtSpExtProb = if(!is.null(startCoextinctions[[species_x]]) &&
!is.na(species_y)) {
                            start[Extinct2, "ExtProb"]}, # that doesn't change
              CoExtSp2 = if(!is.null(startCoextinctions[[species_x]]) &&
!is.na(species_z)) {

as.character(start$ScientificName_accepted[Extinct3])},
              CoExtSp2ExtProb = if(!is.null(startCoextinctions[[species_x]]) &&
!is.na(species_z)) {
                            start[Extinct3, "ExtProb"]}, # that doesn't change#
that doesn't change
              CompSp = as.character(start$ScientificName_accepted[Compensate]),
# change that by the second comp species name
              AbnComp = Abundancediff, # change that by the second comp species
abundance
              BioComp = Biomassdiff, # change that by the second comp species
biomass
              BPc = BPc, # that is just for row completion
              TOC = TOC, # same as BPc
              NH4 = NH4) # same as BPc

      # Add 1 to counter
      counter <- counter + 1

    } # end of the else function for adding rows to output file

      } # end of the else function for this round of compensating species
```

Code S7: Generalised Additive Models (GAMs) of each simulated biodiversity-function relationship following co-extinctions and co-compensations.

```
# Import output data from model simulations ####
# Full model (co-extinctions and co-compensations)
B17_B16_BPc <- read.csv(file.choose()) # ChAOS_2018_B17-
B16_Biomass_Fullmodel_output.csv

B16_B15_BPc <- read.csv(file.choose()) # ChAOS_2018_B16-
B15_Biomass_Fullmodel_output.csv

B15_Xs_BPc <- read.csv(file.choose()) # ChAOS_2018_B15-
Xs_Biomass_Fullmodel_output.csv

Xs_B14_BPc <- read.csv(file.choose()) # ChAOS_2018_Xs-
B14_Biomass_Fullmodel_output.csv

B14_B13_BPc <- read.csv(file.choose()) # ChAOS_2018_B14-
B13_Biomass_Fullmodel_output.csv

B17_B13_BPc <- read.csv(file.choose()) # ChAOS_2018_B17-
B13_Biomass_Fullmodel_output.csv


B17_B13_BPc <- B17_B13_BPc %>%
  add_column(Scenario = "B17-B13")
```

```
B17_B16_BPc <- B17_B16_BPc %>%
  add_column(Scenario = "B17-B16")

B16_B15_BPc <- B16_B15_BPc %>%
  add_column(Scenario = "B16-B15")

B15_Xs_BPc <- B15_Xs_BPc %>%
  add_column(Scenario = "B15-Xs")

Xs_B14_BPc <- Xs_B14_BPc %>%
  add_column(Scenario = "Xs-B14")

B14_B13_BPc <- B14_B13_BPc %>%
  add_column(Scenario = "B14-B13")

B17_B13_BPc <- B17_B13_BPc %>%
  add_column(Scenario = "B17-B13")

# bind all scenarios into one dataframe
ChAOS_Scenarios_BPc <- bind_rows(B17_B16_BPc,
                                 B16_B15_BPc,
                                 B15_Xs_BPc,
                                 Xs_B14_BPc,
                                 B14_B13_BPc,
                                 B17_B13_BPc)

ChAOS_Scenarios_BPc$Scenario <- factor(ChAOS_Scenarios_BPc$Scenario, levels =
c("B17-B16", "B16-B15", "B15-Xs", "Xs-B14", "B14-B13", "B17-B13"))

# GAM ####
# select only necessary columns
ChAOS_Scenarios_BPc_GAM <- ChAOS_Scenarios_BPc %>%
  select(BPc,Nsp,Nsp_active,Scenario) %>%
  filter(complete.cases(.))

rm(ChAOS_Scenarios_BPc)

hist(ChAOS_Scenarios_BPc_GAM$BPc)

ChAOS_Scenarios_BPc_GAM <- ChAOS_Scenarios_BPc_GAM %>%
  mutate(Scenario = factor(Scenario)) %>%
  mutate(Scenario = fct_relevel(Scenario, c("B17-B13", "B17-B16", "B16-B15",
"B15-Xs", "Xs-B14", "B14-B13")))

levels(ChAOS_Scenarios_BPc_GAM$Scenario)

# Max Nsp_active is specific for each scenario (stations have different
starting species richness)
# Also, species richness increases beyond starting community due to incoming
compensating species > species going extinct
max(ChAOS_Scenarios_BPc_GAM[which(ChAOS_Scenarios_BPc_GAM$Scenario == "B17-
B16"),]$Nsp_active) #61
max(ChAOS_Scenarios_BPc_GAM[which(ChAOS_Scenarios_BPc_GAM$Scenario == "B16-
B15"),]$Nsp_active) #53
max(ChAOS_Scenarios_BPc_GAM[which(ChAOS_Scenarios_BPc_GAM$Scenario == "B15-
Xs"),]$Nsp_active) #47
```

```
max(ChAOS_Scenarios_BPc_GAM[which(ChAOS_Scenarios_BPc_GAM$Scenario == "Xs-
B14"),]$Nsp_active) #68
max(ChAOS_Scenarios_BPc_GAM[which(ChAOS_Scenarios_BPc_GAM$Scenario == "B14-
B13"),]$Nsp_active) #54
max(ChAOS_Scenarios_BPc_GAM[which(ChAOS_Scenarios_BPc_GAM$Scenario == "B17-
B13"),]$Nsp_active) #71

## GAMs with interactive term - model fit tested using restricted maximum
likelihodd (REML) ----
# ML or REML fit can be too much computational effort when number of points
>500,000
# BUT the restricted maximum likelihood method is more robust for small sample
sizes and ecological datasets.
# LMtest for comparision of complex and simple GAMs; http://cran.r-
project.org/web/packages/lmtest/index.html
# Can modify the penalty "m" or dimensionality "k" for smooths  -
https://www.seascapemodels.org/rstats/2021/03/27/common-GAM-problems.html
# The bam function in mgcv package is similar to gam but optimized for large
datasets.
# The method fREML stands for "fast Restricted Maximum Likelihood" and is used
in the mgcv package in R, specifically with the bam function, which is designed
for fitting Generalized Additive Models (GAMs) on large datasets.
# The discrete option in bam allows for further speed improvements by
discretizing the smoothing problem, which reduces the computational burden.

library(parallel)

# Create a cluster for parallel processing
cluster <- makeCluster(detectCores() - 1)

# Fit the GAM model with thin-plate regression splines (default in mgcv) and
fREML method
GAM1.1 <- bam(BPc ~ s(Nsp_active, by = Scenario, bs = "tp") +
                s(Nsp_active, bs = "tp") +
                Scenario,
              data = ChAOS_Scenarios_BPc_GAM, method = "fREML", cluster =
cluster)

# Stop the cluster
stopCluster(cluster)

tiff("ChAOS_ExtScn_GAM_model_visualisation.tiff", units="in", width=10,
height=10, res=300)

print(visreg::visreg(GAM1.1, xvar = "Nsp_active", yvar = "BPc",
             by = "Scenario",
             gg = T,
             rug = F,
             partial = T,
             band=F,
             ylab = "BPc", xlab = "Species Richness",
             main = "ChAOS 2018 Co-extinction GAM - Biodiversity-Function
Relationship") +
          facet_wrap(~Scenario, scales = "free") +
          scale_y_log10(limits = c(-2,10)) +
          theme_classic())
```

```
dev.off()

# Different combinations of terms and smooths in reduced model - compare using
maximum likelihood ----

cluster <- makeCluster(detectCores() - 1)

GAM1.1<-bam(BPc~s(Nsp_active, by = Scenario, bs = "tp")+ s(Nsp_active, bs =
"tp") + Scenario,
            data=ChAOS_Scenarios_BPc_GAM, method = "ML", cluster = cluster)

GAM1.1_summary <- summary(GAM1.1)

GAM1.2<-bam(BPc~s(Nsp_active, by = Scenario, bs = "tp") + s(Nsp_active, bs =
"tp"),
            data=ChAOS_Scenarios_BPc_GAM, method = "ML", cluster = cluster)

GAM1.2_summary <- summary(GAM1.2)

GAM1.3<-bam(BPc~s(Nsp_active, by = Scenario, bs = "tp") + Scenario,
            data=ChAOS_Scenarios_BPc_GAM, method = "ML", cluster = cluster)

GAM1.3_summary <- summary(GAM1.3)

GAM1.4<-bam(BPc~s(Nsp_active, bs = "tp") + Scenario,
            data=ChAOS_Scenarios_BPc_GAM, method = "ML", cluster = cluster)

GAM1.4_summary <- summary(GAM1.4)

GAM1.5<-bam(BPc~s(Nsp_active, by = Scenario, bs = "tp"),
            data=ChAOS_Scenarios_BPc_GAM, method = "ML", cluster = cluster)

GAM1.5_summary <- summary(GAM1.5)

GAM1.6<-bam(BPc~s(Nsp_active, bs = "tp"),
            data=ChAOS_Scenarios_BPc_GAM, method = "ML", cluster = cluster)

GAM1.6_summary <- summary(GAM1.6)

GAM1.7<-bam(BPc~Scenario,
            data=ChAOS_Scenarios_BPc_GAM, method = "ML", cluster = cluster)

GAM1.7_summary <- summary(GAM1.7)

GAM1.8<-gam(BPc~1,
            data=ChAOS_Scenarios_BPc_GAM, method = "ML")

GAM1.8_summary <- summary(GAM1.8)

# Stop the cluster
stopCluster(cluster)

model_summaries <- list(GAM1.1_summary, GAM1.2_summary, GAM1.3_summary,
GAM1.4_summary, GAM1.5_summary,
                         GAM1.6_summary, GAM1.7_summary, GAM1.8_summary)
```

```r
AIC_scores <- AIC(GAM1.1, GAM1.2, GAM1.3, GAM1.4, GAM1.5, GAM1.6, GAM1.7,
GAM1.8)

# Create an empty data frame to store model information
    model_info <- tibble(
        response_variable = character(),
        terms = character(),
        n = integer(),
        aic = numeric(),
        r_squared = numeric(),
        ML_smoothing_criterion = numeric()
    )

for(i in 1:length(model_summaries)){
    # Extract information from the current model summary
        response_variable <- as.character(model_summaries[[i]]$formula)[[2]]
        terms <- as.character(model_summaries[[i]]$formula)[[3]]
        n <- model_summaries[[i]]$n
        aic <- AIC_scores$AIC[[i]]
        r_squared <- model_summaries[[i]]$r.sq
        ML_smoothing_criterion <- model_summaries[[i]]$sp.criterion["ML"][[1]]

        # Append the extracted information to the model_info data frame
        model_info <- model_info %>%
            add_row(
                response_variable = response_variable,
                terms = terms,
                n = n,
                aic = aic,
                r_squared = r_squared,
                ML_smoothing_criterion = ML_smoothing_criterion
            )
        }

write.csv(model_info, "ChAOS_ExtScn_GAM_summaries.csv")

# GAM check of best model - BACK TO REML METHOD FOR ECOLOGICAL DATASETS ----
# If the model has not converged, results are likely incorrect. This can happen
when there are too many parameters in the model for not enough data.
# If the model has converged successfully, it means that the estimation process
has likely produced reliable parameter estimates.
# Below, we see a table of basis checking results. This shows a statistical
test for patterns in model residuals, which should be random.
# Each line reports the test results for one smooth. It shows the k value or
number of basis functions, the effective degrees of freedom, a test statistic,
and p-value.
# Here, small p-values indicate that residuals are not randomly distributed.
This often means there are not enough basis functions. This is an approximate
test.
# Always visualize your results too, and compare the k and edf values in
addition to looking at the p-value.
# Q-Q plot, which compares the model residuals to a normal distribution. A
well-fit model's residuals will be close to a straight line.
# On bottom left is a histogram of residuals. We would expect this to have a
symmetrical bell shape.
```

```
# On top-right is a plot of residual values. These should be evenly distributed
around zero.
# Finally, on the bottom-right is plot of response against fitted values. We
don't expect a perfect model, but we do expect the pattern to overlap

# Parametric coefficients are currently for ANOVAs of mean values between B17-
B13 and all other scenarios Need relevellng if want to do all pairwise
comparisons.
# edf: effective degrees of freedom of smooth terms.  This value represents the
complexity of the smooth.
# An edf of 1 is equivalent to a straight line between x and y. An edf of 2 is
equivalent to a quadratic curve, and so on, with higher edfs describing more
wiggly curves.
# The Ref.df and F columns are test statistics used in an ANOVA test to test
overall significance of the smooth.
# a significant smooth term is one where you can not draw a horizontal line
through the 95% confidence interval.

# Create a cluster for parallel processing
cluster <- makeCluster(detectCores() - 1)

# Fit the GAM model with thin-plate regression splines (default in mgcv) and
fREML method
GAM3.1 <- bam(BPc ~ s(Nsp_active, by = Scenario, bs = "tp") +
                Scenario,
              data = ChAOS_Scenarios_BPc_GAM, method = "fREML", cluster =
cluster)

# Stop the cluster
stopCluster(cluster)

sink("ChAOS_ExtScn_GAM_model_check.txt")
print(gam.check(GAM3.1))
sink()

tiff("ChAOS_ExtScn_GAM_model_check.tiff", units="in", width=10, height=10,
res=300)
visibly::plot_gam_check(GAM3.1)
dev.off()

tiff("ChAOS_ExtScn_GAM_model_visualisation.tiff", units="in", width=10,
height=10, res=300)

print(visreg::visreg(GAM3.1, xvar = "Nsp_active", yvar = "BPc",
             by = "Scenario",
             gg = T,
             rug = F,
             partial = T,
             band=F,
             ylab = "BPc", xlab = "Species Richness",
             main = "ChAOS 2018 Co-extinction GAM - Biodiversity-Function
Relationship") +
          facet_wrap(~Scenario, scales = "free") +
          theme_classic())
dev.off()
```

```r
sink("ChAOS_ExtScn_GAM_model_summary.txt")
print(summary(GAM3.1))
sink()

tiff("ChAOS_ExtScn_GAM_model_summary.tiff", units="in", width=10, height=10,
res=300)
plot(GAM3.1, pages = 1, all.terms = TRUE, residuals = TRUE, seWithMean = TRUE,
shift = coef(GAM1.1)[1])
dev.off()

# Partial estimated slopes of BPc as a response to species richness

# Extract the estimated effects of species richness from the GAM model
# Note: We are simplifying the interpretation here, as GAM coefficients are not
as straightforward as in linear models
# Predict the smooth term effects for Nsp_active by Scenario from the GAM model


# Predict the overall effects from the GAM model
gam_pred <- predict(GAM3.1, newdata = ChAOS_Scenarios_BPc_GAM, type =
"response")

# Fit a linear model with the same structure as the best GAM model
lm_model <- lm(BPc ~ Nsp_active * Scenario, data = ChAOS_Scenarios_BPc_GAM)

# Summary of the linear model
summary(lm_model)

# Predict the linear model results
new_data <- ChAOS_Scenarios_BPc_GAM
new_data$Scenario <- factor(new_data$Scenario, levels =
levels(new_data$Scenario))
lm_pred <- predict(lm_model, newdata = new_data)


# Extract the coefficients (partial estimated slopes)
lm_coefficients <- summary(lm_model)$coefficients

# Extract the partial slope of Nsp_active and its interaction with Scenario
lm_partial_slope_Nsp_active <- lm_coefficients["Nsp_active", "Estimate"]
lm_interaction_slopes <- lm_coefficients[grep("Nsp_active:Scenario",
rownames(lm_coefficients)), "Estimate"]

# Print the partial estimated slopes
cat("Partial slope of Nsp_active from the linear model:",
lm_partial_slope_Nsp_active, "\n")
cat("Interaction slopes from the linear model:\n")
print(lm_interaction_slopes)

# Combine data for plotting
plot_data <- ChAOS_Scenarios_BPc_GAM %>%
  mutate(GAM_Pred = gam_pred,
         LM_Pred = lm_pred)

# Create the plot using ggplot2
ggplot(plot_data, aes(x = Nsp_active, y = log(BPc), color = Scenario)) +
```

```r
  geom_point(alpha = 0.2) +
  geom_line(aes(y = log(GAM_Pred)), linetype = "solid", col = "black") +
  geom_line(aes(y = log(LM_Pred)), linetype = "dotted", col = "black") +
  facet_wrap(~ Scenario, scales = "free") +
  labs(title = "Comparison of GAM and LM effects",
       x = "Species richness",
       y = "log(BPc)") +
  theme_classic() +
  theme(legend.position = "none")

ggsave("ChAOS_ExtScn_GAM_LM_comparison_plot.png", ,
       plot = last_plot(),
       width = 10, height = 10, units = "in", dpi = 300)


## Then for the prediction; dummy data ----
# There are six Scenario factors and Nsp_active from 1 to total number of
species for each scenario

DUMDAT<-data.frame(Scenario= factor(c(rep('B17-B16', 200),
                                       rep('B16-B15', 200),
                                       rep('B15-Xs', 200),
                                       rep('Xs-B14', 200),
                                       rep('B14-B13', 200),
                                       rep('B17-B13', 200))
                                     ),
                   Nsp_active=c(rep(seq(from=1, to=61, length=200)),
                                 rep(seq(from=1, to=53, length=200)),
                                 rep(seq(from=1, to=47, length=200)),
                                 rep(seq(from=1, to=68, length=200)),
                                 rep(seq(from=1, to=54, length=200)),
                                 rep(seq(from=1, to=71, length=200))
                                ))

# Check that the species richness matches the scenario
max(DUMDAT[which(DUMDAT$Scenario == "B17-B16"),]$Nsp_active) #61
max(DUMDAT[which(DUMDAT$Scenario == "B16-B15"),]$Nsp_active) #53
max(DUMDAT[which(DUMDAT$Scenario == "B15-Xs"),]$Nsp_active) #47
max(DUMDAT[which(DUMDAT$Scenario == "Xs-B14"),]$Nsp_active) #68
max(DUMDAT[which(DUMDAT$Scenario == "B14-B13"),]$Nsp_active) #54
max(DUMDAT[which(DUMDAT$Scenario == "B17-B13"),]$Nsp_active) #71


## Predict and bind dummy data and prediction ----

P1.1<-predict(GAM3.1, newdata=DUMDAT, se=T)
PRED1.1<-cbind(DUMDAT,P1.1)

PRED1.1$Scenario <- factor(PRED1.1$Scenario, levels = c("B17-B16", "B16-B15",
"B15-Xs", "Xs-B14", "B14-B13", "B17-B13"))

## Plotting ----

ggplot(PRED1.1,aes(x=Nsp_active,y=log(fit), colour=Scenario, group=Scenario,
fill=Scenario))+
  geom_line()+
```

```
  geom_ribbon(aes(ymin= log(fit) + 1.96*(log(se.fit)),
                ymax= log(fit) - 1.96*(log(se.fit))),alpha=.3)+
  theme_classic()+
  scale_y_continuous(name ="", limits = c(-2,10))+
  scale_x_continuous(limits=c(0,71), name="Species richness")+
  # facet_wrap(~Scenario) +
  theme(legend.position="right",
        axis.text.x  = element_text(size=9),
        axis.text.y  = element_text(size=9))+
  scale_colour_discrete(name="Climate\nScenario",
                      breaks=c("B17-B16", "B16-B15", "B15-Xs", "Xs-B14",
"B14-B13", "B17-B13"),
                      labels=c("B17-B16", "B16-B15", "B15-Xs", "Xs-B14",
"B14-B13", "B17-B13"))+
  scale_fill_discrete(name="Climate\nScenario",
                    breaks=c("B17-B16", "B16-B15", "B15-Xs", "Xs-B14", "B14-
B13", "B17-B13"),
                    labels=c("B17-B16", "B16-B15", "B15-Xs", "Xs-B14", "B14-
B13", "B17-B13"))

## logged and facet_wrap ----
BPc.A <- ggplot(PRED1.1,aes(x=Nsp_active,y=log(fit), colour=Scenario,
group=Scenario, fill=Scenario)) +
  geom_vline(data=filter(PRED1.1, Scenario=="B17-B16"), aes(xintercept=52),
lty=2) + # starting community sp.richness
  geom_vline(data=filter(PRED1.1, Scenario=="B16-B15"), aes(xintercept=42),
lty=2) + # starting community sp.richness
  geom_vline(data=filter(PRED1.1, Scenario=="B15-Xs"), aes(xintercept=40),
lty=2) + # starting community sp.richness
  geom_vline(data=filter(PRED1.1, Scenario=="Xs-B14"), aes(xintercept=41),
lty=2) + # starting community sp.richness
  geom_vline(data=filter(PRED1.1, Scenario=="B14-B13"), aes(xintercept=27),
lty=2) + # starting community sp.richness
  geom_vline(data=filter(PRED1.1, Scenario=="B17-B13"), aes(xintercept=52),
lty=2) + # starting community sp.richness
  geom_line()+
  geom_ribbon(aes(ymin= log(fit) + 1.96*(log(se.fit)),
                ymax= log(fit) - 1.96*(log(se.fit))),alpha=.3)+
  theme_bw()+
  scale_y_continuous(name ="log(BPc)")+
  scale_x_continuous(limits=c(0,71), name="Species richness")+
  facet_grid(~Scenario) +
  theme(legend.position="right",
        axis.text.x  = element_text(size=9),
        axis.text.y  = element_text(size=9))+
  scale_colour_discrete(name="Climate\nScenario",
                      breaks=c("B17-B16", "B16-B15", "B15-Xs", "Xs-B14",
"B14-B13", "B17-B13"),
                      labels=c("B17-B16", "B16-B15", "B15-Xs", "Xs-B14",
"B14-B13", "B17-B13"))+
  scale_fill_discrete(name="Climate\nScenario",
                    breaks=c("B17-B16", "B16-B15", "B15-Xs", "Xs-B14", "B14-
B13", "B17-B13"),
                    labels=c("B17-B16", "B16-B15", "B15-Xs", "Xs-B14", "B14-
B13", "B17-B13"))
```

```
### Add space between local scenarios and region-wide scenario (B17-B13) ----
gt = ggplot_gtable(ggplot_build(BPc.A))
gt$widths[14] = 4*gt$widths[1]
grid::grid.draw(gt)
```

Code S8: Model output figures

```
# Import output data from model simulations ####
# Full model (co-extinctions and co-compensations)
B17_B16_BPc <- read.csv(file.choose()) # ChAOS_2018_B17-
B16_Biomass_Fullmodel_output.csv
B17_B16_Contributions <- readRDS(file.choose()) # ChAOS_2018_B17-
B16_Biomass_Fullmodel_contributions.rds
B16_B15_BPc <- read.csv(file.choose()) # ChAOS_2018_B16-
B15_Biomass_Fullmodel_output.csv
B16_B15_Contributions <- readRDS(file.choose()) # ChAOS_2018_B16-
B15_Biomass_Fullmodel_contributions.rds
B15_Xs_BPc <- read.csv(file.choose()) # ChAOS_2018_B15-
Xs_Biomass_Fullmodel_output.csv
B15_Xs_Contributions <- readRDS(file.choose()) # ChAOS_2018_B15-
Xs_Biomass_Fullmodel_contributions.rds
Xs_B14_BPc <- read.csv(file.choose()) # ChAOS_2018_Xs-
B14_Biomass_Fullmodel_output.csv
Xs_B14_Contributions <- readRDS(file.choose()) # ChAOS_2018_Xs-
B14_Biomass_Fullmodel_contributions.rds
B14_B13_BPc <- read.csv(file.choose()) # ChAOS_2018_B14-
B13_Biomass_Fullmodel_output.csv
B14_B13_Contributions <- readRDS(file.choose()) # ChAOS_2018_B14-
B13_Biomass_Fullmodel_contributions.rds
B17_B13_BPc <- read.csv(file.choose()) #ChAOS_2018_B17-
B13_Biomass_Fullmodel_output.csv
B17_B13_Contributions <- readRDS(file.choose()) # ChAOS_2018_B17-
B13_Biomass_Fullmodel_contributions.rds

# Reduced model (co-extinctions and no compensations)
B17_B16_CoExt_NoComp_BPc <- read.csv(file.choose()) # ChAOS_2018_B17-
B16_Biomass_Coextmodel_output.csv
B17_B16_CoExt_NoComp_Contributions <- readRDS(file.choose()) # ChAOS_2018_B17-
B16_Biomass_Coextmodel_contributions.rds
B16_B15_CoExt_NoComp_BPc <- read.csv(file.choose()) # ChAOS_2018_B16-
B15_Biomass_Coextmodel_output.csv
B16_B15_CoExt_NoComp_Contributions <- readRDS(file.choose()) # ChAOS_2018_B16-
B15_Biomass_Coextmodel_contributions.rds
B15_Xs_CoExt_NoComp_BPc <- read.csv(file.choose()) # ChAOS_2018_B15-
Xs_Biomass_Coextmodel_output.csv
B15_Xs_CoExt_NoComp_Contributions <- readRDS(file.choose()) # ChAOS_2018_B15-
Xs_Biomass_Coextmodel_contributions.rds
Xs_B14_CoExt_NoComp_BPc <- read.csv(file.choose()) # ChAOS_2018_Xs-
B14_Biomass_Coextmodel_output.csv
Xs_B14_CoExt_NoComp_Contributions <- readRDS(file.choose()) # ChAOS_2018_Xs-
B14_Biomass_Coextmodel_contributions.rds
B14_B13_CoExt_NoComp_BPc <- read.csv(file.choose()) # ChAOS_2018_B14-
B13_Biomass_Coextmodel_output.csv
B14_B13_CoExt_NoComp_Contributions <- readRDS(file.choose()) # ChAOS_2018_B14-
B13_Biomass_Coextmodel_contributions.rds
B17_B13_CoExt_NoComp_BPc <- read.csv(file.choose()) # ChAOS_2018_B17-
B13_Biomass_Coextmodel_output.csv
```

```
B17_B13_CoExt_NoComp_Contributions <- readRDS(file.choose()) # ChAOS_2018_B17-
B13_Biomass_Coextmodel_contributions.rds

# Simple model (no co-extinctions and no compensations)
B17_B16_NoCoExt_NoComp_BPc <- read.csv(file.choose()) # ChAOS_2018_B17-
B16_Biomass_emptymodel_output.csv
B17_B16_NoCoExt_NoComp_Contributions <- readRDS(file.choose()) #
ChAOS_2018_B17-B16_Biomass_emptymodel_contributions.rds
B16_B15_NoCoExt_NoComp_BPc <- read.csv(file.choose()) # ChAOS_2018_B16-
B15_Biomass_emptymodel_output.csv
B16_B15_NoCoExt_NoComp_Contributions <- readRDS(file.choose()) #
ChAOS_2018_B16-B15_Biomass_emptymodel_contributions.rds
B15_Xs_NoCoExt_NoComp_BPc <- read.csv(file.choose()) # ChAOS_2018_B15-
Xs_Biomass_emptymodel_output.csv
B15_Xs_NoCoExt_NoComp_Contributions <- readRDS(file.choose()) # ChAOS_2018_B15-
Xs_Biomass_emptymodel_contributions.rds
Xs_B14_NoCoExt_NoComp_BPc <- read.csv(file.choose()) # ChAOS_2018_Xs-
B14_Biomass_emptymodel_output.csv
Xs_B14_NoCoExt_NoComp_Contributions <- readRDS(file.choose()) # ChAOS_2018_Xs-
B14_Biomass_emptymodel_contributions.rds
B14_B13_NoCoExt_NoComp_BPc <- read.csv(file.choose()) # ChAOS_2018_B14-
B13_Biomass_emptymodel_output.csv
B14_B13_NoCoExt_NoComp_Contributions <- readRDS(file.choose()) #
ChAOS_2018_B14-B13_Biomass_emptymodel_contributions.rds
B17_B13_NoCoExt_NoComp_BPc <- read.csv(file.choose()) # ChAOS_2018_B17-
B13_Biomass_emptymodel_output.csv
B17_B13_NoCoExt_NoComp_Contributions <- readRDS(file.choose()) #
ChAOS_2018_B17-B13_Biomass_emptymodel_contributions.rds


# Figure 1: No coextinctions, no compensations, full models ####

plot1 <- ggplot(B17_B16_NoCoExt_NoComp_BPc,aes(x=Nsp_active,y=log(BPc)))+
  geom_point(colour="grey",alpha=0.5)+
  stat_density2d(aes(fill=after_stat(level), alpha=after_stat(level)),
               size=3, bins=20, geom='polygon') +
  geom_vline(xintercept = 17, col = "red", linetype = "dashed", linewidth =
0.8) +
  geom_vline(xintercept = 52, col = "green", linewidth = 0.8) +
  scale_x_continuous(limits = c(0,70)) +
  scale_y_continuous(limits = c(-2,8)) +
  geom_text(size = 12, x = 60, y = -0.1, label = "a") +
  theme_classic()+
  theme(legend.position = "none", panel.grid.major = element_blank(),
panel.grid.minor = element_blank(),
        panel.background = element_blank(), axis.line = element_line(colour =
"black")) +
  labs(x = "Species richness",
       y = "log(BPc)")

plot2 <- ggplot(B17_B16_CoExt_NoComp_BPc,aes(x=Nsp_active,y=log(BPc)))+
  geom_point(colour="grey",alpha=0.5)+
  stat_density2d(aes(fill=after_stat(level), alpha=after_stat(level)),
               size=3, bins=20, geom='polygon') +
  geom_vline(xintercept = 17, col = "red", linetype = "dashed", linewidth =
0.8) +
```

```
  geom_vline(xintercept = 52, col = "green", linewidth = 0.8) +
  scale_x_continuous(limits = c(0,70)) +
  scale_y_continuous(limits = c(-2,8)) +
    geom_text(size = 12, x = 60, y = -0.1, label = "g") +
  theme_classic()+
  theme(legend.position = "none", panel.grid.major = element_blank(),
panel.grid.minor = element_blank(),
        panel.background = element_blank(), axis.line = element_line(colour =
"black")) +
  labs(x = "Species richness",
       y = "log(BPc)")

plot3 <- ggplot(B17_B16_BPc,aes(x=Nsp_active,y=log(BPc)))+
  geom_point(colour="grey",alpha=0.5)+
  stat_density2d(aes(fill=after_stat(level), alpha=after_stat(level)),
                 size=3, bins=20, geom='polygon') +
  geom_vline(xintercept = 17, col = "red", linetype = "dashed", linewidth =
0.8) +
  geom_vline(xintercept = 52, col = "green", linewidth = 0.8) +
  scale_x_continuous(limits = c(0,70)) +
  scale_y_continuous(limits = c(-2,8)) +
    geom_text(size = 12, x = 60, y = -0.1, label = "m") +
  theme_classic()+
  theme(legend.position = "none", panel.grid.major = element_blank(),
panel.grid.minor = element_blank(),
        panel.background = element_blank(), axis.line = element_line(colour =
"black")) +
  labs(x = "Species richness",
       y = "log(BPc)")

plot4 <- ggplot(B16_B15_NoCoExt_NoComp_BPc,aes(x=Nsp_active,y=log(BPc)))+
  geom_point(colour="grey",alpha=0.5)+
  stat_density2d(aes(fill=after_stat(level), alpha=after_stat(level)),
                 size=3, bins=20, geom='polygon') +
  geom_vline(xintercept = 15, col = "red", linetype = "dashed", linewidth =
0.8) +
  geom_vline(xintercept = 42, col = "green", linewidth = 0.8) +
  scale_x_continuous(limits = c(0,70)) +
  scale_y_continuous(limits = c(-2,8)) +
    geom_text(size = 12, x = 60, y = -0.1, label = "b") +
  theme_classic()+
  theme(legend.position = "none", panel.grid.major = element_blank(),
panel.grid.minor = element_blank(),
        panel.background = element_blank(), axis.line = element_line(colour =
"black")) +
  labs(x = "Species richness",
       y = "log(BPc)")

plot5 <- ggplot(B16_B15_CoExt_NoComp_BPc,aes(x=Nsp_active,y=log(BPc)))+
  geom_point(colour="grey",alpha=0.5)+
  stat_density2d(aes(fill=after_stat(level), alpha=after_stat(level)),
                 size=3, bins=20, geom='polygon') +
  geom_vline(xintercept = 15, col = "red", linetype = "dashed", linewidth =
0.8) +
  geom_vline(xintercept = 42, col = "green", linewidth = 0.8) +
  scale_x_continuous(limits = c(0,70)) +
```

```
  scale_y_continuous(limits = c(-2,8)) +
    geom_text(size = 12, x = 60, y = -0.1, label = "h") +
  theme_classic()+
  theme(legend.position = "none", panel.grid.major = element_blank(),
panel.grid.minor = element_blank(),
        panel.background = element_blank(), axis.line = element_line(colour =
"black")) +
  labs(x = "Species richness",
       y = "Log(BPc)")

plot6 <- ggplot(B16_B15_BPc,aes(x=Nsp_active,y=log(BPc)))+
  geom_point(colour="grey",alpha=0.5)+
  stat_density2d(aes(fill=after_stat(level), alpha=after_stat(level)),
                 size=3, bins=20, geom='polygon') +
  geom_vline(xintercept = 15, col = "red", linetype = "dashed", linewidth =
0.8) +
  geom_vline(xintercept = 42, col = "green", linewidth = 0.8) +
  scale_x_continuous(limits = c(0,70)) +
  scale_y_continuous(limits = c(-2,8)) +
    geom_text(size = 12, x = 60, y = -0.1, label = "n") +
  theme_classic()+
  theme(legend.position = "none", panel.grid.major = element_blank(),
panel.grid.minor = element_blank(),
        panel.background = element_blank(), axis.line = element_line(colour =
"black")) +
  labs(x = "Species richness",
       y = "Log(BPc)")

plot7 <- ggplot(B15_Xs_NoCoExt_NoComp_BPc,aes(x=Nsp_active,y=log(BPc)))+
  geom_point(colour="grey",alpha=0.5)+
  stat_density2d(aes(fill=after_stat(level), alpha=after_stat(level)),
                 size=3, bins=20, geom='polygon') +
  geom_vline(xintercept = 16, col = "red", linetype = "dashed", linewidth =
0.8) +
  geom_vline(xintercept = 40, col = "green", linewidth = 0.8) +
  scale_x_continuous(limits = c(0,70)) +
  scale_y_continuous(limits = c(-2,8)) +
    geom_text(size = 12, x = 60, y = -0.1, label = "c") +
  theme_classic()+
  theme(legend.position = "none", panel.grid.major = element_blank(),
panel.grid.minor = element_blank(),
        panel.background = element_blank(), axis.line = element_line(colour =
"black")) +
  labs(x = "Species richness",
       y = "Log(BPc)")

plot8 <- ggplot(B15_Xs_CoExt_NoComp_BPc,aes(x=Nsp_active,y=log(BPc)))+
  geom_point(colour="grey",alpha=0.5)+
  stat_density2d(aes(fill=after_stat(level), alpha=after_stat(level)),
                 size=3, bins=20, geom='polygon') +
  geom_vline(xintercept = 16, col = "red", linetype = "dashed", linewidth =
0.8) +
  geom_vline(xintercept = 40, col = "green", linewidth = 0.8) +
  scale_x_continuous(limits = c(0,70)) +
  scale_y_continuous(limits = c(-2,8)) +
    geom_text(size = 12, x = 60, y = -0.1, label = "i") +
```

```
  theme_classic()+
  theme(legend.position = "none", panel.grid.major = element_blank(),
panel.grid.minor = element_blank(),
        panel.background = element_blank(), axis.line = element_line(colour =
"black")) +
  labs(x = "Species richness",
       y = "Log(BPc)")

plot9 <- ggplot(B15_Xs_BPc,aes(x=Nsp_active,y=log(BPc)))+
  geom_point(colour="grey",alpha=0.5)+
  stat_density2d(aes(fill=after_stat(level), alpha=after_stat(level)),
                 size=3, bins=20, geom='polygon') +
  geom_vline(xintercept = 16, col = "red", linetype = "dashed", linewidth =
0.8) +
  geom_vline(xintercept = 40, col = "green", linewidth = 0.8) +
  scale_x_continuous(limits = c(0,70)) +
  scale_y_continuous(limits = c(-2,8)) +
    geom_text(size = 12, x = 60, y = -0.1, label = "o") +
  theme_classic()+
  theme(legend.position = "none", panel.grid.major = element_blank(),
panel.grid.minor = element_blank(),
        panel.background = element_blank(), axis.line = element_line(colour =
"black")) +
  labs(x = "Species richness",
       y = "Log(BPc)")

plot10 <- ggplot(Xs_B14_NoCoExt_NoComp_BPc,aes(x=Nsp_active,y=log(BPc)))+
  geom_point(colour="grey",alpha=0.5)+
  stat_density2d(aes(fill=after_stat(level), alpha=after_stat(level)),
                 size=3, bins=20, geom='polygon') +
  geom_vline(xintercept = 10, col = "red", linetype = "dashed", linewidth =
0.8) +
  geom_vline(xintercept = 41, col = "green", linewidth = 0.8) +
  scale_x_continuous(limits = c(0,70)) +
  scale_y_continuous(limits = c(-2,8)) +
    geom_text(size = 12, x = 60, y = -0.1, label = "d") +
  theme_classic()+
  theme(legend.position = "none", panel.grid.major = element_blank(),
panel.grid.minor = element_blank(),
        panel.background = element_blank(), axis.line = element_line(colour =
"black")) +
  labs(x = "Species richness",
       y = "Log(BPc)")

plot11 <- ggplot(Xs_B14_CoExt_NoComp_BPc,aes(x=Nsp_active,y=log(BPc)))+
  geom_point(colour="grey",alpha=0.5)+
  stat_density2d(aes(fill=after_stat(level), alpha=after_stat(level)),
                 size=3, bins=20, geom='polygon') +
  geom_vline(xintercept = 10, col = "red", linetype = "dashed", linewidth =
0.8) +
  geom_vline(xintercept = 41, col = "green", linewidth = 0.8) +
  scale_x_continuous(limits = c(0,70)) +
  scale_y_continuous(limits = c(-2,8)) +
    geom_text(size = 12, x = 60, y = -0.1, label = "j") +
  theme_classic()+
  theme(legend.position = "none", panel.grid.major = element_blank(),
```

```
panel.grid.minor = element_blank(),
        panel.background = element_blank(), axis.line = element_line(colour =
"black")) +
  labs(x = "Species richness",
       y = "Log(BPc)")

plot12 <- ggplot(Xs_B14_BPc,aes(x=Nsp_active,y=log(BPc)))+
  geom_point(colour="grey",alpha=0.5)+
  stat_density2d(aes(fill=after_stat(level), alpha=after_stat(level)),
                 size=3, bins=20, geom='polygon') +
  geom_vline(xintercept = 10, col = "red", linetype = "dashed", linewidth =
0.8) +
  geom_vline(xintercept = 41, col = "green", linewidth = 0.8) +
  scale_x_continuous(limits = c(0,70)) +
  scale_y_continuous(limits = c(-2,8)) +
    geom_text(size = 12, x = 60, y = -0.1, label = "p") +
  theme_classic()+
  theme(legend.position = "none", panel.grid.major = element_blank(),
panel.grid.minor = element_blank(),
        panel.background = element_blank(), axis.line = element_line(colour =
"black")) +
  labs(x = "Species richness",
       y = "Log(BPc)")

plot13 <- ggplot(B14_B13_NoCoExt_NoComp_BPc,aes(x=Nsp_active,y=log(BPc)))+
  geom_point(colour="grey",alpha=0.5)+
  stat_density2d(aes(fill=after_stat(level), alpha=after_stat(level)),
                 size=3, bins=20, geom='polygon') +
  geom_vline(xintercept = 11, col = "red", linetype = "dashed", linewidth =
0.8) +
  geom_vline(xintercept = 27, col = "green", linewidth = 0.8) +
  scale_x_continuous(limits = c(0,70)) +
  scale_y_continuous(limits = c(-2,8)) +
    geom_text(size = 12, x = 60, y = -0.1, label = "e") +
  theme_classic()+
  theme(legend.position = "none", panel.grid.major = element_blank(),
panel.grid.minor = element_blank(),
        panel.background = element_blank(), axis.line = element_line(colour =
"black")) +
  labs(x = "Species richness",
       y = "Log(BPc)")

plot14 <- ggplot(B14_B13_CoExt_NoComp_BPc,aes(x=Nsp_active,y=log(BPc)))+
  geom_point(colour="grey",alpha=0.5)+
  stat_density2d(aes(fill=after_stat(level), alpha=after_stat(level)),
                 size=3, bins=20, geom='polygon') +
  geom_vline(xintercept = 11, col = "red", linetype = "dashed", linewidth =
0.8) +
  geom_vline(xintercept = 27, col = "green", linewidth = 0.8) +
  scale_x_continuous(limits = c(0,70)) +
  scale_y_continuous(limits = c(-2,8)) +
    geom_text(size = 12, x = 60, y = -0.1, label = "k") +
  theme_classic()+
  theme(legend.position = "none", panel.grid.major = element_blank(),
panel.grid.minor = element_blank(),
        panel.background = element_blank(), axis.line = element_line(colour =
```

```
"black")) +
  labs(x = "Species richness",
       y = "Log(BPc)")

plot15 <- ggplot(B14_B13_BPc,aes(x=Nsp_active,y=log(BPc)))+
  geom_point(colour="grey",alpha=0.5)+
  stat_density2d(aes(fill=after_stat(level), alpha=after_stat(level)),
                 size=3, bins=20, geom='polygon') +
  geom_vline(xintercept = 11, col = "red", linetype = "dashed", linewidth =
0.8) +
  geom_vline(xintercept = 27, col = "green", linewidth = 0.8) +
  scale_x_continuous(limits = c(0,70)) +
  scale_y_continuous(limits = c(-2,8)) +
    geom_text(size = 12, x = 60, y = -0.1, label = "q") +
  theme_classic()+
  theme(legend.position = "none", panel.grid.major = element_blank(),
panel.grid.minor = element_blank(),
        panel.background = element_blank(), axis.line = element_line(colour =
"black")) +
  labs(x = "Species richness",
       y = "Log(BPc)")

plot16 <- ggplot(B17_B13_NoCoExt_NoComp_BPc,aes(x=Nsp_active,y=log(BPc)))+
  geom_point(colour="grey",alpha=0.5)+
  stat_density2d(aes(fill=after_stat(level), alpha=after_stat(level)),
                 size=3, bins=20, geom='polygon') +
  geom_vline(xintercept = 11, col = "red", linetype = "dashed", linewidth =
0.8) +
  geom_vline(xintercept = 52, col = "green", linewidth = 0.8) +
  scale_x_continuous(limits = c(0,70)) +
  scale_y_continuous(limits = c(-2,8)) +
    geom_text(size = 12, x = 60, y = -0.1, label = "f") +
  theme_classic()+
  theme(legend.position = "none", panel.grid.major = element_blank(),
panel.grid.minor = element_blank(),
        panel.background = element_blank(), axis.line = element_line(colour =
"black")) +
  labs(x = "Species richness",
       y = "Log(BPc)")

plot17 <- ggplot(B17_B13_CoExt_NoComp_BPc,aes(x=Nsp_active,y=log(BPc)))+
  geom_point(colour="grey",alpha=0.5)+
  stat_density2d(aes(fill=after_stat(level), alpha=after_stat(level)),
                 size=3, bins=20, geom='polygon') +
  geom_vline(xintercept = 11, col = "red", linetype = "dashed", linewidth =
0.8) +
  geom_vline(xintercept = 52, col = "green", linewidth = 0.8) +
  scale_x_continuous(limits = c(0,70)) +
  scale_y_continuous(limits = c(-2,8)) +
  geom_text(size = 12, x = 60, y = -0.1, label = "l") +
  theme_classic()+
  theme(legend.position = "none", panel.grid.major = element_blank(),
panel.grid.minor = element_blank(),
        panel.background = element_blank(), axis.line = element_line(colour =
"black")) +
  labs(x = "Species richness",
```

```
      y = "log(BPc)")

plot18 <- ggplot(B17_B13_BPc,aes(x=Nsp_active,y=log(BPc)))+
  geom_point(colour="grey",alpha=0.5)+
  stat_density2d(aes(fill=after_stat(level), alpha=after_stat(level)),
                 size=3, bins=20, geom='polygon') +
  geom_vline(xintercept = 11, col = "red", linetype = "dashed", linewidth =
0.8) +
  geom_vline(xintercept = 52, col = "green", linewidth = 0.8) +
  scale_x_continuous(limits = c(0,70)) +
  scale_y_continuous(limits = c(-2,8)) +
  geom_text(size = 12, x = 60, y = -0.1, label = "r") +
  theme_classic()+
  theme(legend.position = "none", panel.grid.major = element_blank(),
panel.grid.minor = element_blank(),
        panel.background = element_blank(), axis.line = element_line(colour =
"black")) +
  labs(x = "Species richness",
      y = "log(BPc)")

ylab0 <- "Extinctions ordered by....."

Super_BPc_plot <-  wrap_elements(grid::textGrob("B17-B16", vjust = 5, rot = 0,
gp = grid::gpar(fontsize = 16))) +  wrap_elements(grid::textGrob("B16-B15",
vjust = 5, rot = 0, gp = grid::gpar(fontsize = 16))) +
  wrap_elements(grid::textGrob("B15-Xs", vjust = 5, rot = 0, gp =
grid::gpar(fontsize = 16))) +
  wrap_elements(grid::textGrob("Xs-B14", vjust = 5, rot = 0, gp =
grid::gpar(fontsize = 16))) +
  wrap_elements(grid::textGrob("B14-B13", vjust = 5, rot = 0, gp =
grid::gpar(fontsize = 16))) + plot_spacer() +
  wrap_elements(grid::textGrob("B17-B13", vjust = 5, rot = 0, gp =
grid::gpar(fontsize = 16))) +
  plot_spacer() +
  (plot1 & theme(axis.title.x = element_blank(),
                      axis.title.y = element_blank(),
                      axis.text.x = element_blank(),
                      axis.text.y = element_text(size = 16))) +
  (plot4 & theme(axis.title.x = element_blank(),
                      axis.title.y = element_blank(),
                      axis.text.x = element_blank(),
                      axis.text.y = element_blank())) +
  (plot7 & theme(axis.title.x = element_blank(),
                      axis.title.y = element_blank(),
                      axis.text.x = element_blank(),
                      axis.text.y = element_blank())) +
  (plot10 & theme(axis.title.x = element_blank(),
                      axis.title.y = element_blank(),
                      axis.text.x = element_blank(),
                      axis.text.y = element_blank())) +
  (plot13 & theme(axis.title.x = element_blank(),
                      axis.title.y = element_blank(),
                      axis.text.x = element_blank(),
                      axis.text.y = element_blank())) + plot_spacer() +
  (plot16 & theme(axis.title.x = element_blank(),
                      axis.title.y = element_blank(),
```

```r
                         axis.text.x = element_blank(),
                         axis.text.y = element_text(size = 16))) +
  wrap_elements(grid::textGrob("Climate vulnerability", vjust = 4, rot = -90,
gp = grid::gpar(fontsize = 16))) +
  (plot2 & theme(axis.title.x = element_blank(),
                         axis.title.y = element_blank(),
                         axis.text.x = element_blank(),
                         axis.text.y = element_text(size = 16))) +
  (plot5 & theme(axis.title.x = element_blank(),
                         axis.title.y = element_blank(),
                         axis.text.x = element_blank(),
                         axis.text.y = element_blank())) +
  (plot8 & theme(axis.title.x = element_blank(),
                         axis.title.y = element_blank(),
                         axis.text.x = element_blank(),
                         axis.text.y = element_blank())) +
  (plot11 & theme(axis.title.x = element_blank(),
                         axis.title.y = element_blank(),
                         axis.text.x = element_blank(),
                         axis.text.y = element_blank())) +
  (plot14 & theme(axis.title.x = element_blank(),
                         axis.title.y = element_blank(),
                         axis.text.x = element_blank(),
                         axis.text.y = element_blank()))  + plot_spacer() +
  (plot17 & theme(axis.title.x = element_blank(),
                         axis.title.y = element_blank(),
                         axis.text.x = element_blank(),
                         axis.text.y = element_text(size = 16))) +
  wrap_elements(grid::textGrob("Climate vulnerability \n and co-extinctions",
vjust = 1.8, rot = -90, gp = grid::gpar(fontsize = 16))) +
  (plot3 & theme(axis.title.x = element_blank(),
                         axis.title.y = element_blank(),
                         axis.text.x = element_text(size = 16),
                         axis.text.y = element_text(size = 16))) +
  (plot6 & theme(axis.title.x = element_blank(),
                         axis.title.y = element_blank(),
                         axis.text.x = element_text(size = 16),
                         axis.text.y = element_blank())) +
  (plot9 & theme(axis.title.x = element_blank(),
                         axis.title.y = element_blank(),
                         axis.text.x = element_text(size = 16),
                         axis.text.y = element_blank())) +
  (plot12 & theme(axis.title.x = element_blank(),
                         axis.title.y = element_blank(),
                         axis.text.x = element_text(size = 16),
                         axis.text.y = element_blank())) +
  (plot15 & theme(axis.title.x = element_blank(),
                         axis.title.y = element_blank(),
                         axis.text.x = element_text(size = 16),
                         axis.text.y = element_blank())) + plot_spacer() +
  (plot18 & theme(axis.title.x = element_blank(),
                         axis.title.y = element_blank(),
                         axis.text.x = element_text(size = 16),
                         axis.text.y = element_text(size = 16))) +
  wrap_elements(grid::textGrob("Climate vulnerability, \n co-extinctions and \n
co-compensations", vjust = 1.2, rot = -90, gp = grid::gpar(fontsize = 16))) +
```

```
  plot_layout(ncol = 8, nrow = 4, widths = c(1,1,1,1,1,0.3,1,1))

ggsave(
  "Figure1.tiff",
  plot = Super_BPc_plot,
  device = tiff,
  scale = 2,
  width = 24,
  height = 12,
  units = c("cm"),
  dpi = 300
)


# Figure 2: GAMs, compensation graphs ####
## Split GAMS by Scenario ####
split_GAMs <- split(PRED1.1, PRED1.1$Scenario)

B17_B16_GAM <- ggplot(split_GAMs[["B17-B16"]], aes(x=Nsp_active,y=log(fit))) +
  geom_vline(aes(xintercept=52), lty=1, col="green", linewidth = 0.8) +
    geom_vline(aes(xintercept=17), lty=2, col="red", linewidth = 0.8) +
  geom_line(col = "blue") +
  geom_ribbon(aes(ymin= log(fit) + 1.96*(log(se.fit)),
                ymax= log(fit) - 1.96*(log(se.fit))),alpha=.3, fill =
"blue")+
  theme_classic()+
  theme(axis.line.y = element_line()) +
  scale_y_continuous(name ="log(BPc)", limits = c(-1,16), breaks =
c(0,5,10,15))+
  scale_x_continuous(limits=c(0,71), name="Species richness")+
  geom_text(size = 12, x = 65, y = 14, label = "a") +
  theme(legend.position="right",
        axis.text.x  = element_text(size=9),
        axis.text.y  = element_text(size=9))

B16_B15_GAM <- ggplot(split_GAMs[["B16-B15"]], aes(x=Nsp_active,y=log(fit)))+
  geom_vline(aes(xintercept=42), lty=1, col="green", linewidth = 0.8) +
    geom_vline(aes(xintercept=15), lty=2, col="red", linewidth = 0.8) +
  geom_line(col = "blue") +
  geom_ribbon(aes(ymin= log(fit) + 1.96*(log(se.fit)),
                ymax= log(fit) - 1.96*(log(se.fit))),alpha=.3, fill =
"blue")+
  theme_classic()+
  theme(axis.line.y = element_line()) +
  scale_y_continuous(name ="log(BPc)", limits = c(-1,16), breaks =
c(0,5,10,15))+
  scale_x_continuous(limits=c(0,71), name="Species richness")+
  geom_text(size = 12, x = 65, y = 14, label = "b") +
  theme(legend.position="right",
        axis.text.x  = element_text(size=9),
        axis.text.y  = element_text(size=9))


B15_Xs_GAM <- ggplot(split_GAMs[["B15-Xs"]], aes(x=Nsp_active,y=log(fit)))+
  geom_vline(aes(xintercept=40), lty=1, col="green", linewidth = 0.8) +
  geom_vline(aes(xintercept=16), lty=2, col="red", linewidth = 0.8) +
```

```r
  geom_line(col = "blue") +
  geom_ribbon(aes(ymin= log(fit) + 1.96*(log(se.fit)),
                  ymax= log(fit) - 1.96*(log(se.fit))),alpha=.3, fill =
"blue")+
  theme_classic()+
  theme(axis.line.y = element_line()) +
  scale_y_continuous(name ="Log(BPc)", limits = c(-1,16), breaks =
c(0,5,10,15))+
  scale_x_continuous(limits=c(0,71), name="Species richness")+
  geom_text(size = 12, x = 65, y = 14, label = "c") +
  theme(legend.position="right",
        axis.text.x  = element_text(size=9),
        axis.text.y  = element_text(size=9))

Xs_B14_GAM <- ggplot(split_GAMs[["Xs-B14"]], aes(x=Nsp_active,y=log(fit)))+
  geom_vline(aes(xintercept=41), lty=1, col="green", linewidth = 0.8) +
    geom_vline(aes(xintercept=10), lty=2, col="red", linewidth = 0.8) +
  geom_line(col = "blue") +
  geom_ribbon(aes(ymin= log(fit) + 1.96*(log(se.fit)),
                  ymax= log(fit) - 1.96*(log(se.fit))),alpha=.3, fill =
"blue")+
  theme_classic()+
  theme(axis.line.y = element_line()) +
  scale_y_continuous(name ="Log(BPc)", limits = c(-1,16), breaks =
c(0,5,10,15))+
  scale_x_continuous(limits=c(0,71), name="Species richness")+
  geom_text(size = 12, x = 65, y = 14, label = "d") +
  theme(legend.position="right",
        axis.text.x  = element_text(size=9),
        axis.text.y  = element_text(size=9))

B14_B13_GAM <- ggplot(split_GAMs[["B14-B13"]], aes(x=Nsp_active,y=log(fit)))+
  geom_vline(aes(xintercept=27), lty=1, col="green", linewidth = 0.8) +
    geom_vline(aes(xintercept=11), lty=2, col="red", linewidth = 0.8) +
  geom_line(col = "blue") +
  geom_ribbon(aes(ymin= log(fit) + 1.96*(log(se.fit)),
                  ymax= log(fit) - 1.96*(log(se.fit))),alpha=.3, fill =
"blue")+
  theme_classic()+
  theme(axis.line.y = element_line()) +
  scale_y_continuous(name ="Log(BPc)", limits = c(-1,16), breaks =
c(0,5,10,15))+
  scale_x_continuous(limits=c(0,71), name="Species richness")+
  geom_text(size = 12, x = 65, y = 14, label = "e") +
  theme(legend.position="right",
        axis.text.x  = element_text(size=9),
        axis.text.y  = element_text(size=9))

B17_B13_GAM <- ggplot(split_GAMs[["B17-B13"]], aes(x=Nsp_active,y=log(fit)))+
  geom_vline(aes(xintercept=52), lty=1, col="green", linewidth = 0.8) +
  geom_vline(aes(xintercept=11), lty=2, col="red", linewidth = 0.8) +
  geom_line(col = "blue") +
  geom_ribbon(aes(ymin= log(fit) + 1.96*(log(se.fit)),
                  ymax= log(fit) - 1.96*(log(se.fit))),alpha=.3, fill =
"blue")+
  theme_classic()+
```

```
  theme(axis.line.y = element_line()) +
  scale_y_continuous(name ="Log(BPc)", limits = c(-1,16), breaks =
c(0,5,10,15))+
  scale_x_continuous(limits=c(0,71), name="Species richness")+
  geom_text(size = 12, x = 65, y = 14, label = "f") +
  theme(legend.position="right",
        axis.text.x  = element_text(size=9),
        axis.text.y  = element_text(size=9))

## Compensation graphs ####

B17_B16_Compensation_graph <- B17_B16_BPc %>%
  filter(!is.na(CompSp)) %>%
  select(Simulation, Nsp, Nsp_active, CompRep, CompSp) %>%
  group_by(Nsp_active) %>%
  summarise(CompRep.mean = mean(CompRep),
                           CompRep.sd = sd(CompRep),
                           CompRep.se = sd(CompRep)/sqrt(n()),
                           CompRep.max = max(CompRep),
            n = n()) %>%
  mutate(CompProp_mean = CompRep.mean/Nsp_active,
         CompProp_se = CompRep.se/Nsp_active) %>%
  ggplot(aes(x= Nsp_active, y=CompRep.mean))+
  geom_line(col="gold", aes(y = CompRep.mean), linewidth = 1.2) +
  geom_ribbon(fill="gold", alpha = 0.3, aes(ymin = CompRep.mean-CompRep.se,
ymax = CompRep.mean+CompRep.se)) +
  geom_line(col="purple", aes(y = CompProp_mean*10), linewidth = 1.2) +
  geom_ribbon(fill="purple", alpha = 0.3, aes(ymin = (CompProp_mean-
CompProp_se)*10, ymax = (CompProp_mean+CompProp_se)*10)) +
  geom_vline(xintercept = 17, col = "red", linetype = "dashed", linewidth =
0.8, alpha = 0.8) +
  geom_vline(xintercept = 52, col = "green", linewidth = 0.8, alpha = 0.8) +
  scale_x_continuous(limits = c(0,70)) +
  scale_y_continuous(limits = c(0,16),
                     sec.axis = sec_axis(~./10, name = "Proportion of \n
Compensation species")) +
  geom_text(size = 12, x = 65, y = 14, label = "g") +
  theme_classic()+
  theme(legend.position="right", panel.grid.major = element_blank(),
panel.grid.minor = element_blank(),
                    panel.background = element_blank(), axis.line =
element_line(colour = "black")) +
  labs(x = "Species richness",
                  y = "No. of \n compensating \n species")

B16_B15_Compensation_graph <-  B16_B15_BPc %>%
  filter(!is.na(CompSp)) %>%
  select(Simulation, Nsp, Nsp_active, CompRep, CompSp) %>%
  group_by(Nsp_active) %>%
  summarise(CompRep.mean = mean(CompRep),
                           CompRep.sd = sd(CompRep),
                           CompRep.se = sd(CompRep)/sqrt(n()),
                           CompRep.max = max(CompRep),
            n = n()) %>%
  mutate(CompProp_mean = CompRep.mean/Nsp_active,
         CompProp_se = CompRep.se/Nsp_active) %>%
```

```
  ggplot(aes(x= Nsp_active, y=CompRep.mean))+
  geom_line(col="gold", aes(y = CompRep.mean), linewidth = 1.2) +
  geom_ribbon(fill="gold", alpha = 0.3, aes(ymin = CompRep.mean-CompRep.se,
ymax = CompRep.mean+CompRep.se)) +
  geom_line(col="purple", aes(y = CompProp_mean*10), linewidth = 1.2) +
  geom_ribbon(fill="purple", alpha = 0.3, aes(ymin = (CompProp_mean-
CompProp_se)*10, ymax = (CompProp_mean+CompProp_se)*10)) +
  geom_vline(xintercept = 15, col = "red", linetype = "dashed", linewidth =
0.8, alpha = 0.8) +
  geom_vline(xintercept = 42, col = "green", linewidth = 0.8, alpha = 0.8) +
  scale_x_continuous(limits = c(0,70)) +
  scale_y_continuous(limits = c(0,16),
                     sec.axis = sec_axis(~./10, name = "Proportion of \n
compensating \n species")) +
  geom_text(size = 12, x = 65, y = 14, label = "h") +
  theme_classic()+
  theme(legend.position="right", panel.grid.major = element_blank(),
panel.grid.minor = element_blank(),
                    panel.background = element_blank(), axis.line =
element_line(colour = "black")) +
  labs(x = "Species richness",
                   y = "No. of \n compensating \n species")

B15_Xs_Compensation_graph <- B15_Xs_BPc %>%
  filter(!is.na(CompSp)) %>%
  select(Simulation, Nsp, Nsp_active, CompRep, CompSp) %>%
  group_by(Nsp_active) %>%
  summarise(CompRep.mean = mean(CompRep),
                            CompRep.sd = sd(CompRep),
                            CompRep.se = sd(CompRep)/sqrt(n()),
                            CompRep.max = max(CompRep),
            n = n()) %>%
  mutate(CompProp_mean = CompRep.mean/Nsp_active,
         CompProp_se = CompRep.se/Nsp_active) %>%
  ggplot(aes(x= Nsp_active, y=CompRep.mean))+
  geom_line(col="gold", aes(y = CompRep.mean), linewidth = 1.2) +
  geom_ribbon(fill="gold", alpha = 0.3, aes(ymin = CompRep.mean-CompRep.se,
ymax = CompRep.mean+CompRep.se)) +
  geom_line(col="purple", aes(y = CompProp_mean*10), linewidth = 1.2) +
  geom_ribbon(fill="purple", alpha = 0.3, aes(ymin = (CompProp_mean-
CompProp_se)*10, ymax = (CompProp_mean+CompProp_se)*10)) +
  geom_vline(xintercept = 16, col = "red", linetype = "dashed", linewidth =
0.8, alpha = 0.8) +
  geom_vline(xintercept = 40, col = "green", linewidth = 0.8, alpha = 0.8) +
  scale_x_continuous(limits = c(0,70)) +
  scale_y_continuous(limits = c(0,16),
                     sec.axis = sec_axis(~./10, name = "Proportion of \n
compensating \n species")) +
  geom_text(size = 12, x = 65, y = 14, label = "i") +
  theme_classic()+
  theme(legend.position="right", panel.grid.major = element_blank(),
panel.grid.minor = element_blank(),
                    panel.background = element_blank(), axis.line =
element_line(colour = "black")) +
  labs(x = "Species richness",
                   y = "No. of \n compensating \n species")
```

```
Xs_B14_Compensation_graph <- Xs_B14_BPc %>%
  filter(!is.na(CompSp)) %>%
  select(Simulation, Nsp, Nsp_active, CompRep, CompSp) %>%
  group_by(Nsp_active) %>%
  summarise(CompRep.mean = mean(CompRep),
                          CompRep.sd = sd(CompRep),
                          CompRep.se = sd(CompRep)/sqrt(n()),
                          CompRep.max = max(CompRep),
          n = n()) %>%
  mutate(CompProp_mean = CompRep.mean/Nsp_active,
         CompProp_se = CompRep.se/Nsp_active) %>%
  ggplot(aes(x= Nsp_active, y=CompRep.mean))+
  geom_line(col="gold", aes(y = CompRep.mean), linewidth = 1.2) +
  geom_ribbon(fill="gold", alpha = 0.3, aes(ymin = CompRep.mean-CompRep.se,
ymax = CompRep.mean+CompRep.se)) +
  geom_line(col="purple", aes(y = CompProp_mean*10), linewidth = 1.2) +
  geom_ribbon(fill="purple", alpha = 0.3, aes(ymin = (CompProp_mean-
CompProp_se)*10, ymax = (CompProp_mean+CompProp_se)*10)) +
  geom_vline(xintercept = 10, col = "red", linetype = "dashed", linewidth =
0.8, alpha = 0.8) +
  geom_vline(xintercept = 41, col = "green", linewidth = 0.8, alpha = 0.8) +
  scale_x_continuous(limits = c(0,70)) +
  scale_y_continuous(limits = c(0,16),
                     sec.axis = sec_axis(~./10, name = "Proportion of \n
compensating \n species")) +
  geom_text(size = 12, x = 65, y = 14, label = "j") +
  theme_classic()+
  theme(legend.position="right", panel.grid.major = element_blank(),
panel.grid.minor = element_blank(),
                    panel.background = element_blank(), axis.line =
element_line(colour = "black")) +
  labs(x = "Species richness",
                  y = "No. of \n compensating \n species")

B14_B13_Compensation_graph <-B14_B13_BPc %>%
  filter(!is.na(CompSp)) %>%
  select(Simulation, Nsp, Nsp_active, CompRep, CompSp) %>%
  group_by(Nsp_active) %>%
  summarise(CompRep.mean = mean(CompRep),
                          CompRep.sd = sd(CompRep),
                          CompRep.se = sd(CompRep)/sqrt(n()),
                          CompRep.max = max(CompRep),
          n = n()) %>%
  mutate(CompProp_mean = CompRep.mean/Nsp_active,
         CompProp_se = CompRep.se/Nsp_active) %>%
  ggplot(aes(x= Nsp_active, y=CompRep.mean))+
  geom_line(col="gold", aes(y = CompRep.mean), linewidth = 1.2) +
  geom_ribbon(fill="gold", alpha = 0.3, aes(ymin = CompRep.mean-CompRep.se,
ymax = CompRep.mean+CompRep.se)) +
  geom_line(col="purple", aes(y = CompProp_mean*10), linewidth = 1.2) +
  geom_ribbon(fill="purple", alpha = 0.3, aes(ymin = (CompProp_mean-
CompProp_se)*10, ymax = (CompProp_mean+CompProp_se)*10)) +
  geom_vline(xintercept = 11, col = "red", linetype = "dashed", linewidth =
0.8, alpha = 0.8) +
  geom_vline(xintercept = 27, col = "green", linewidth = 0.8, alpha = 0.8) +
```

```
  scale_x_continuous(limits = c(0,70)) +
  scale_y_continuous(limits = c(0,16),
                     sec.axis = sec_axis(~./10, name = "Proportion of \n
compensating \n species")) +
  geom_text(size = 12, x = 65, y = 14, label = "k") +
  theme_classic()+
  theme(legend.position="right", panel.grid.major = element_blank(),
panel.grid.minor = element_blank(),
                    panel.background = element_blank(), axis.line =
element_line(colour = "black")) +
  labs(x = "Species richness",
                  y = "No. of \n compensating \n species")

B17_B13_Compensation_graph <- B17_B13_BPc %>%
  filter(!is.na(CompSp)) %>%
  select(Simulation, Nsp, Nsp_active, CompRep, CompSp) %>%
  group_by(Nsp_active) %>%
  summarise(CompRep.mean = mean(CompRep),
                            CompRep.sd = sd(CompRep),
                            CompRep.se = sd(CompRep)/sqrt(n()),
                            CompRep.max = max(CompRep),
            n = n()) %>%
  mutate(CompProp_mean = CompRep.mean/Nsp_active,
         CompProp_se = CompRep.se/Nsp_active) %>%
  ggplot(aes(x= Nsp_active, y=CompRep.mean))+
  geom_line(col="gold", aes(y = CompRep.mean), linewidth = 1.2) +
  geom_ribbon(fill="gold", alpha = 0.3, aes(ymin = CompRep.mean-CompRep.se,
ymax = CompRep.mean+CompRep.se)) +
  geom_line(col="purple", aes(y = CompProp_mean*10), linewidth = 1.2) +
  geom_ribbon(fill="purple", alpha = 0.3, aes(ymin = (CompProp_mean-
CompProp_se)*10, ymax = (CompProp_mean+CompProp_se)*10)) +
  geom_vline(xintercept = 11, col = "red", linetype = "dashed", linewidth =
0.8, alpha = 0.8) +
  geom_vline(xintercept = 52, col = "green", linewidth = 0.8, alpha = 0.8) +
  scale_x_continuous(limits = c(0,70)) +
  scale_y_continuous(limits = c(0,16),
                     sec.axis = sec_axis(~./10, name = "Proportion of \n
compensating \n species")) +
  geom_text(size = 12, x = 65, y = 14, label = "l") +
  theme_classic()+
  theme(legend.position="right", panel.grid.major = element_blank(),
panel.grid.minor = element_blank(),
                    panel.background = element_blank(), axis.line =
element_line(colour = "black")) +
  labs(x = "Species richness",
                  y = "No. of \n compensating \n species")

## Mi ####
B17_B16_Mi <- B17_B16_Contributions %>%
  filter(AiSim != 0) %>% #remove species that are not alive
  unique() %>% # remove duplicates of same species during co-compensations
  select(Simulation,Iteration,Nsp_active, Mi, Ri) %>%
  group_by(Nsp_active, Simulation,Iteration, Mi) %>%
  dplyr::summarise(freq = n()) %>%
  group_by(Nsp_active,Mi) %>%
  dplyr::summarise(mean = mean(freq),
```

```
                    median = median(freq)) %>%
  filter(!is.na(Mi)) %>%
  ggplot() +
  geom_bar(aes(x = Nsp_active, fill = as.factor(Mi), y = mean), na.rm = T,
position = "fill", stat = "identity") +
  geom_vline(xintercept = 17, col = "red", linetype = "dashed", linewidth =
0.8, alpha = 0.8) +
  geom_vline(xintercept = 52, col = "green", linewidth = 0.8, alpha = 0.8) +
  geom_text(size = 12, x = 65, y = 0.9, label = "m") +
  scale_x_continuous(name = "Species richness", limits = c(0,70)) +
  scale_y_continuous(name = "Relative Mi") +
  scale_fill_brewer(palette = "Purples", name = "Mobility classification",
labels = c("Fixed tube", "Limited movement", "Slow, free movement", "Burrow
system")) +
  theme_classic()

B16_B15_Mi <- B16_B15_Contributions %>%
  filter(AiSim != 0) %>% #remove species that are not alive
  unique() %>% # remove duplicates of same species during co-compensations
  select(Simulation,Iteration,Nsp_active, Mi, Ri) %>%
  group_by(Nsp_active, Simulation,Iteration, Mi) %>%
  dplyr::summarise(freq = n()) %>%
  group_by(Nsp_active,Mi) %>%
  dplyr::summarise(mean = mean(freq),
                    median = median(freq)) %>%
  filter(!is.na(Mi)) %>%
  ggplot() +
  geom_bar(aes(x = Nsp_active, fill = as.factor(Mi), y = mean), na.rm = T,
position = "fill", stat = "identity") +
  geom_vline(xintercept = 15, col = "red", linetype = "dashed", linewidth =
0.8, alpha = 0.8) +
  geom_vline(xintercept = 42, col = "green", linewidth = 0.8, alpha = 0.8) +
  geom_text(size = 12, x = 65, y = 0.9, label = "n") +
  scale_x_continuous(name = "Species richness", limits = c(0,70)) +
  scale_y_continuous(name = "Relative Mi") +
  scale_fill_brewer(palette = "Purples", name = "Mobility classification",
labels = c("Fixed tube", "Limited movement", "Slow, free movement", "Burrow
system")) +
  theme_classic()

B15_Xs_Mi <- B15_Xs_Contributions %>%
  filter(AiSim != 0) %>% #remove species that are not alive
  unique() %>% # remove duplicates of same species during co-compensations
  select(Simulation,Iteration,Nsp_active, Mi, Ri) %>%
  group_by(Nsp_active, Simulation,Iteration, Mi) %>%
  dplyr::summarise(freq = n()) %>%
  group_by(Nsp_active,Mi) %>%
  dplyr::summarise(mean = mean(freq),
                    median = median(freq)) %>%
  filter(!is.na(Mi)) %>%
  ggplot() +
  geom_bar(aes(x = Nsp_active, fill = as.factor(Mi), y = mean), na.rm = T,
position = "fill", stat = "identity") +
  geom_vline(xintercept = 16, col = "red", linetype = "dashed", linewidth =
0.8, alpha = 0.8) +
  geom_vline(xintercept = 40, col = "green", linewidth = 0.8, alpha = 0.8) +
```

```r
  geom_text(size = 12, x = 65, y = 0.9, label = "o") +
  scale_x_continuous(name = "Species richness", limits = c(0,70)) +
  scale_y_continuous(name = "Relative Mi") +
  scale_fill_brewer(palette = "Purples", name = "Mobility classification",
labels = c("Fixed tube", "Limited movement", "Slow, free movement", "Burrow
system")) +
  theme_classic()

Xs_B14_Mi <- Xs_B14_Contributions %>%
  filter(AiSim != 0) %>% #remove species that are not alive
  unique() %>% # remove duplicates of same species during co-compensations
  select(Simulation,Iteration,Nsp_active, Mi, Ri) %>%
  group_by(Nsp_active, Simulation,Iteration, Mi) %>%
  dplyr::summarise(freq = n()) %>%
  group_by(Nsp_active,Mi) %>%
  dplyr::summarise(mean = mean(freq),
                   median = median(freq)) %>%
  filter(!is.na(Mi)) %>%
  ggplot() +
  geom_bar(aes(x = Nsp_active, fill = as.factor(Mi), y = mean), na.rm = T,
position = "fill", stat = "identity") +
  geom_vline(xintercept = 10, col = "red", linetype = "dashed", linewidth =
0.8, alpha = 0.8) +
  geom_vline(xintercept = 41, col = "green", linewidth = 0.8, alpha = 0.8) +
  geom_text(size = 12, x = 65, y = 0.9, label = "p") +
  scale_x_continuous(name = "Species richness", limits = c(0,70)) +
  scale_y_continuous(name = "Relative Mi") +
  scale_fill_brewer(palette = "Purples", name = "Mobility classification",
labels = c("Fixed tube", "Limited movement", "Slow, free movement", "Burrow
system")) +
  theme_classic()

B14_B13_Mi <- B14_B13_Contributions %>%
  filter(AiSim != 0) %>% #remove species that are not alive
  unique() %>% # remove duplicates of same species during co-compensations
  select(Simulation,Iteration,Nsp_active, Mi, Ri) %>%
  group_by(Nsp_active, Simulation,Iteration, Mi) %>%
  dplyr::summarise(freq = n()) %>%
  group_by(Nsp_active,Mi) %>%
  dplyr::summarise(mean = mean(freq),
                   median = median(freq)) %>%
  filter(!is.na(Mi)) %>%
  ggplot() +
  geom_bar(aes(x = Nsp_active, fill = as.factor(Mi), y = mean), na.rm = T,
position = "fill", stat = "identity") +
  geom_vline(xintercept = 11, col = "red", linetype = "dashed", linewidth =
0.8, alpha = 0.8) +
  geom_vline(xintercept = 27, col = "green", linewidth = 0.8, alpha = 0.8) +
  geom_text(size = 12, x = 65, y = 0.9, label = "q") +
  scale_x_continuous(name = "Species richness", limits = c(0,70)) +
  scale_y_continuous(name = "Relative Mi") +
  scale_fill_brewer(palette = "Purples", name = "Mobility classification",
labels = c("Fixed tube", "Limited movement", "Slow, free movement", "Burrow
system")) +
  theme_classic()
```

```r
B17_B13_Mi <- B17_B13_Contributions %>%
  filter(AiSim != 0) %>% #remove species that are not alive
  unique() %>% # remove duplicates of same species during co-compensations
  select(Simulation,Iteration,Nsp_active, Mi, Ri) %>%
  group_by(Nsp_active, Simulation,Iteration, Mi) %>%
  dplyr::summarise(freq = n()) %>%
  group_by(Nsp_active,Mi) %>%
  dplyr::summarise(mean = mean(freq),
                   median = median(freq)) %>%
  filter(!is.na(Mi)) %>%
  ggplot() +
  geom_bar(aes(x = Nsp_active, fill = as.factor(Mi), y = mean), na.rm = T,
position = "fill", stat = "identity") +
  geom_vline(xintercept = 11, col = "red", linetype = "dashed", linewidth =
0.8, alpha = 0.8) +
  geom_vline(xintercept = 52, col = "green", linewidth = 0.8, alpha = 0.8) +
  geom_text(size = 12, x = 65, y = 0.9, label = "r") +
  scale_x_continuous(name = "Species richness", limits = c(0,70)) +
  scale_y_continuous(name = "Relative Mi") +
  scale_fill_brewer(palette = "Purples", name = "Mobility classification",
labels = c("Fixed tube", "Limited movement", "Slow, free movement", "Burrow
system")) +
  theme_classic()

## Ri ####
B17_B16_Ri <- B17_B16_Contributions %>%
  filter(AiSim != 0) %>% #remove species that are not alive
  unique() %>% # remove duplicates of same species during co-compensations
  select(Simulation,Iteration,Nsp_active, Mi, Ri) %>%
  group_by(Nsp_active, Simulation,Iteration, Ri) %>%
  dplyr::summarise(freq = n()) %>%
  group_by(Nsp_active,Ri) %>%
  dplyr::summarise(mean = mean(freq),
                   median = median(freq)) %>%
  filter(!is.na(Ri)) %>%
  ggplot() +
  geom_bar(aes(x = Nsp_active, fill = as.factor(Ri), y = mean), na.rm = T,
position = "fill", stat = "identity") +
  geom_vline(xintercept = 17, col = "red", linetype = "dashed", linewidth =
0.8, alpha = 0.8) +
  geom_vline(xintercept = 52, col = "green", linewidth = 0.8, alpha = 0.8) +
  geom_text(size = 12, x = 65, y = 0.9, label = "s") +
  scale_x_continuous(name = "Species richness", limits = c(0,70)) +
  scale_y_continuous(name = "Relative Ri") +
  scale_fill_brewer(name = "Reworking mode", labels = c("Epifauna", "Surficial
Modifier", "Upward/Downward Conveyor", "Biodiffusor", "Regenerator")) +
  theme_classic()

B16_B15_Ri <- B16_B15_Contributions %>%
  filter(AiSim != 0) %>% #remove species that are not alive
  unique() %>% # remove duplicates of same species during co-compensations
  select(Simulation,Iteration,Nsp_active, Mi, Ri) %>%
  group_by(Nsp_active, Simulation,Iteration, Ri) %>%
  dplyr::summarise(freq = n()) %>%
  group_by(Nsp_active,Ri) %>%
  dplyr::summarise(mean = mean(freq),
```

```
                    median = median(freq)) %>%
  filter(!is.na(Ri)) %>%
  ggplot() +
  geom_bar(aes(x = Nsp_active, fill = as.factor(Ri), y = mean), na.rm = T,
position = "fill", stat = "identity") +
  geom_vline(xintercept = 15, col = "red", linetype = "dashed", linewidth =
0.8, alpha = 0.8) +
  geom_vline(xintercept = 42, col = "green", linewidth = 0.8, alpha = 0.8) +
  geom_text(size = 12, x = 65, y = 0.9, label = "t") +
  scale_x_continuous(name = "Species richness", limits = c(0,70)) +
  scale_y_continuous(name = "Relative Ri") +
  scale_fill_brewer(name = "Reworking mode", labels = c("Epifauna", "Surficial
Modifier", "Upward/Downward Conveyor", "Biodiffusor", "Regenerator")) +
  theme_classic()

B15_Xs_Ri <- B15_Xs_Contributions %>%
  filter(AiSim != 0) %>% #remove species that are not alive
  unique() %>% # remove duplicates of same species during co-compensations
  select(Simulation,Iteration,Nsp_active, Mi, Ri) %>%
  group_by(Nsp_active, Simulation,Iteration, Ri) %>%
  dplyr::summarise(freq = n()) %>%
  group_by(Nsp_active,Ri) %>%
  dplyr::summarise(mean = mean(freq),
                    median = median(freq)) %>%
  filter(!is.na(Ri)) %>%
  ggplot() +
  geom_bar(aes(x = Nsp_active, fill = as.factor(Ri), y = mean), na.rm = T,
position = "fill", stat = "identity") +
  geom_vline(xintercept = 16, col = "red", linetype = "dashed", linewidth =
0.8, alpha = 0.8) +
  geom_vline(xintercept = 40, col = "green", linewidth = 0.8, alpha = 0.8) +
  geom_text(size = 12, x = 65, y = 0.9, label = "u") +
  scale_x_continuous(name = "Species richness", limits = c(0,70)) +
  scale_y_continuous(name = "Relative Ri") +
 scale_fill_brewer(name = "Reworking mode", labels = c("Epifauna", "Surficial
Modifier", "Upward/Downward Conveyor", "Biodiffusor", "Regenerator")) +
  theme_classic()

Xs_B14_Ri <- Xs_B14_Contributions %>%
  filter(AiSim != 0) %>% #remove species that are not alive
  unique() %>% # remove duplicates of same species during co-compensations
  select(Simulation,Iteration,Nsp_active, Mi, Ri) %>%
  group_by(Nsp_active, Simulation,Iteration, Ri) %>%
  dplyr::summarise(freq = n()) %>%
  group_by(Nsp_active,Ri) %>%
  dplyr::summarise(mean = mean(freq),
                    median = median(freq)) %>%
  filter(!is.na(Ri)) %>%
  ggplot() +
  geom_bar(aes(x = Nsp_active, fill = as.factor(Ri), y = mean), na.rm = T,
position = "fill", stat = "identity") +
  geom_vline(xintercept = 10, col = "red", linetype = "dashed", linewidth =
0.8, alpha = 0.8) +
  geom_vline(xintercept = 41, col = "green", linewidth = 0.8, alpha = 0.8) +
  geom_text(size = 12, x = 65, y = 0.9, label = "v") +
  scale_x_continuous(name = "Species richness", limits = c(0,70)) +
```

```r
  scale_y_continuous(name = "Relative Ri") +
  scale_fill_brewer(name = "Reworking mode", labels = c("Epifauna", "Surficial
Modifier", "Upward/Downward Conveyor", "Biodiffusor", "Regenerator")) +
  theme_classic()

B14_B13_Ri <- B14_B13_Contributions %>%
  filter(AiSim != 0) %>% #remove species that are not alive
  unique() %>% # remove duplicates of same species during co-compensations
  select(Simulation,Iteration,Nsp_active, Mi, Ri) %>%
  group_by(Nsp_active, Simulation,Iteration, Ri) %>%
  dplyr::summarise(freq = n()) %>%
  group_by(Nsp_active,Ri) %>%
  dplyr::summarise(mean = mean(freq),
                   median = median(freq)) %>%
  filter(!is.na(Ri)) %>%
  ggplot() +
  geom_bar(aes(x = Nsp_active, fill = as.factor(Ri), y = mean), na.rm = T,
position = "fill", stat = "identity") +
  geom_vline(xintercept = 11, col = "red", linetype = "dashed", linewidth =
0.8, alpha = 0.8) +
  geom_vline(xintercept = 27, col = "green", linewidth = 0.8, alpha = 0.8) +
  geom_text(size = 12, x = 65, y = 0.9, label = "w") +
  scale_x_continuous(name = "Species richness", limits = c(0,70)) +
  scale_y_continuous(name = "Relative Ri") +
  scale_fill_brewer(name = "Reworking mode", labels = c("Epifauna", "Surficial
Modifier", "Upward/Downward Conveyor", "Biodiffusor", "Regenerator")) +
  theme_classic()

B17_B13_Ri <- B17_B13_Contributions %>%
  filter(AiSim != 0) %>% #remove species that are not alive
  unique() %>% # remove duplicates of same species during co-compensations
  select(Simulation,Iteration,Nsp_active, Mi, Ri) %>%
  group_by(Nsp_active, Simulation,Iteration, Ri) %>%
  dplyr::summarise(freq = n()) %>%
  group_by(Nsp_active,Ri) %>%
  dplyr::summarise(mean = mean(freq),
                   median = median(freq)) %>%
  filter(!is.na(Ri)) %>%
  ggplot() +
  geom_bar(aes(x = Nsp_active, fill = as.factor(Ri), y = mean), na.rm = T,
position = "fill", stat = "identity") +
  geom_vline(xintercept = 11, col = "red", linetype = "dashed", linewidth =
0.8, alpha = 0.8) +
  geom_vline(xintercept = 52, col = "green", linewidth = 0.8, alpha = 0.8) +
  geom_text(size = 12, x = 65, y = 0.9, label = "x") +
  scale_x_continuous(name = "Species richness", limits = c(0,70)) +
  scale_y_continuous(name = "Relative Ri") +
  scale_fill_brewer(name = "Reworking mode", labels = c("Epifauna", "Surficial
Modifier", "Upward/Downward Conveyor", "Biodiffusor", "Regenerator")) +
  theme_classic()

GAM_BPc_plot <- wrap_elements(grid::textGrob("B17-B16", vjust = 4, rot = 0, gp
= grid::gpar(fontsize = 16))) +  wrap_elements(grid::textGrob("B16-B15", vjust
= 4, rot = 0, gp = grid::gpar(fontsize = 16))) +
  wrap_elements(grid::textGrob("B15-Xs", vjust = 4, rot = 0, gp =
grid::gpar(fontsize = 16))) +
```

```r
  wrap_elements(grid::textGrob("Xs-B14", vjust = 4, rot = 0, gp =
grid::gpar(fontsize = 16))) +
  wrap_elements(grid::textGrob("B14-B13", vjust = 4, rot = 0, gp =
grid::gpar(fontsize = 16))) +
    plot_spacer() +
  wrap_elements(grid::textGrob("B17-B13", vjust = 4, rot = 0, gp =
grid::gpar(fontsize = 16))) +
    B17_B16_GAM + theme(axis.title.x = element_blank(), axis.text.x.bottom =
element_blank(), axis.title.y = element_text(size = 20)) +
  B16_B15_GAM + theme(axis.title.y = element_blank(), axis.text.y.left =
element_blank(), axis.title.x = element_blank(), axis.text.x.bottom =
element_blank()) +
  B15_Xs_GAM + theme(axis.title.y = element_blank(), axis.text.y.left =
element_blank(), axis.title.x = element_blank(), axis.text.x.bottom =
element_blank()) +
  Xs_B14_GAM + theme(axis.title.y = element_blank(), axis.text.y.left =
element_blank(), axis.title.x = element_blank(), axis.text.x.bottom =
element_blank()) +
  B14_B13_GAM + theme(axis.title.y = element_blank(), axis.text.y.left =
element_blank(), axis.title.x = element_blank(), axis.text.x.bottom =
element_blank()) + plot_spacer() +
  B17_B13_GAM + theme(axis.title.y = element_blank(), axis.text.y.left =
element_blank(), axis.text.y.right = element_blank(), axis.title.x =
element_blank(), axis.text.x.bottom = element_blank()) +
  B17_B16_Compensation_graph + theme(axis.title.x = element_blank(),
axis.title.y.left = element_text(size = 20), axis.text.y.right =
element_blank(), axis.title.y.right = element_blank(), axis.text.x.bottom =
element_blank()) +
  B16_B15_Compensation_graph + theme(axis.title.y = element_blank(),
axis.text.y.left = element_blank(), axis.text.y.right = element_blank(),
axis.title.x = element_blank(), axis.text.x.bottom = element_blank()) +
  B15_Xs_Compensation_graph + theme(axis.title.y = element_blank(),
axis.text.y.left = element_blank(), axis.text.y.right = element_blank(),
axis.title.x = element_blank(), axis.text.x.bottom = element_blank()) +
  Xs_B14_Compensation_graph + theme(axis.title.y = element_blank(),
axis.text.y.left = element_blank(), axis.text.y.right = element_blank(),
axis.title.x = element_blank(), axis.text.x.bottom = element_blank()) +
  B14_B13_Compensation_graph + theme(axis.title.y = element_blank(),
axis.text.y.left = element_blank(), axis.text.y.right = element_blank(),
axis.title.x = element_blank(), axis.text.x.bottom = element_blank()) +
plot_spacer() +
  B17_B13_Compensation_graph + theme(axis.title.y.left = element_blank(),
axis.text.y.left = element_blank(), axis.title.y.right = element_text(size =
20), axis.title.x = element_blank(), axis.text.x.bottom = element_blank()) +
  B17_B16_Mi + theme(axis.title.x = element_blank(), axis.text.x.bottom =
element_blank(), axis.title.y = element_text(size = 20)) +
  B16_B15_Mi + theme(axis.title.y = element_blank(), axis.text.y.left =
element_blank(), axis.title.x = element_blank(), axis.text.x.bottom =
element_blank()) +
  B15_Xs_Mi + theme(axis.title.y = element_blank(), axis.text.y.left =
element_blank(), axis.title.x = element_blank(), axis.text.x.bottom =
element_blank()) +
  Xs_B14_Mi + theme(axis.title.y = element_blank(), axis.text.y.left =
element_blank(), axis.title.x = element_blank(), axis.text.x.bottom =
element_blank()) +
  B14_B13_Mi + theme(axis.title.y = element_blank(), axis.text.y.left =
```

```r
element_blank(), axis.title.x = element_blank(), axis.text.x.bottom =
element_blank()) + plot_spacer() +
  B17_B13_Mi + theme(axis.title.y = element_blank(), axis.text.y.left =
element_blank(), axis.title.x = element_blank(), axis.text.x.bottom =
element_blank()) +
  B17_B16_Ri + theme(axis.title.x = element_blank(), axis.title.y =
element_text(size = 20)) +
  B16_B15_Ri + theme(axis.title.y = element_blank(), axis.text.y.left =
element_blank(), axis.title.x = element_blank()) +
  B15_Xs_Ri + theme(axis.title.y = element_blank(), axis.text.y.left =
element_blank(), axis.title.x = element_blank()) +
  Xs_B14_Ri + theme(axis.title.y = element_blank(), axis.text.y.left =
element_blank(), axis.title.x = element_blank()) +
  B14_B13_Ri + theme(axis.title.y = element_blank(), axis.text.y.left =
element_blank(), axis.title.x = element_blank()) + plot_spacer() +
  B17_B13_Ri + theme(axis.title.y = element_blank(), axis.text.y.left =
element_blank(), axis.title.x = element_blank()) +
  plot_layout(guides = "collect", ncol = 7, widths = c(1,1,1,1,1,0.3,1))

GAM_BPc_plot & theme(axis.text.x = element_text(size = 16),
                       axis.text.y = element_text(size = 16))

ggsave(
  "Figure2.tiff",
  plot = last_plot(),
  device = tiff,
  scale = 2,
  width = 22,
  height = 16,
  units = c("cm"),
  dpi = 300
)


# Figure 3: Taxonomy graphs ####

B17_B16_Contributions$species <- gsub("_", " ", B17_B16_Contributions$species)

top_B17_B16_Contributions <- B17_B16_Contributions %>%
  group_by(species, Nsp_active) %>%
  summarise(mean_contribution = mean(species_contribution, na.rm = T))

top20_B17_B16_Contributions <- top_B17_B16_Contributions %>%
  group_by(species) %>%
  summarise(Nsp_active = Nsp_active,
            mean_contribution = mean_contribution,
            species_mean_contribution = mean(mean_contribution),
            species_starting_contribution = mean(mean_contribution[Nsp_active
== 52]),
            species_final_contribution = mean(mean_contribution[Nsp_active ==
17]))


# select only top 20 species based off species mean contribution at the
starting richness
top20_B17_B16_Contributions <- top20_B17_B16_Contributions %>%
```

```
  arrange(-species_starting_contribution) %>%
  group_by(Nsp_active) %>%
  slice(1:20)

rm(top_B17_B16_Contributions)

B16_B15_Contributions$species <- gsub("_", " ", B16_B15_Contributions$species)

top_B16_B15_Contributions <- B16_B15_Contributions %>%
  group_by(species, Nsp_active) %>%
  summarise(mean_contribution = mean(species_contribution, na.rm = T))

top20_B16_B15_Contributions <- top_B16_B15_Contributions %>%
  group_by(species) %>%
  summarise(Nsp_active = Nsp_active,
            mean_contribution = mean_contribution,
            species_mean_contribution = mean(mean_contribution),
            species_starting_contribution = mean(mean_contribution[Nsp_active
== 42]),
            species_final_contribution = mean(mean_contribution[Nsp_active ==
15]))


# select only top 20 species based off species mean contribution at the
starting richness
top20_B16_B15_Contributions <- top20_B16_B15_Contributions %>%
  arrange(-species_starting_contribution) %>%
  group_by(Nsp_active) %>%
  slice(1:20)

rm(top_B16_B15_Contributions)

B15_Xs_Contributions$species <- gsub("_", " ", B15_Xs_Contributions$species)

top_B15_Xs_Contributions <- B15_Xs_Contributions %>%
  group_by(species, Nsp_active) %>%
  summarise(mean_contribution = mean(species_contribution, na.rm = T))

top20_B15_Xs_Contributions <- top_B15_Xs_Contributions %>%
  group_by(species) %>%
  summarise(Nsp_active = Nsp_active,
            mean_contribution = mean_contribution,
            species_mean_contribution = mean(mean_contribution),
            species_starting_contribution = mean(mean_contribution[Nsp_active
== 40]),
            species_final_contribution = mean(mean_contribution[Nsp_active ==
16]))


# select only top 20 species based off species mean contribution at the
starting richness
top20_B15_Xs_Contributions <- top20_B15_Xs_Contributions %>%
  arrange(-species_starting_contribution) %>%
  group_by(Nsp_active) %>%
  slice(1:20)
```

```
rm(top_B15_Xs_Contributions)

Xs_B14_Contributions$species <- gsub("_", " ", Xs_B14_Contributions$species)

top_Xs_B14_Contributions <- Xs_B14_Contributions %>%
  group_by(species, Nsp_active) %>%
  summarise(mean_contribution = mean(species_contribution, na.rm = T))

top20_Xs_B14_Contributions <- top_Xs_B14_Contributions %>%
  group_by(species) %>%
  summarise(Nsp_active = Nsp_active,
            mean_contribution = mean_contribution,
            species_mean_contribution = mean(mean_contribution),
            species_starting_contribution = mean(mean_contribution[Nsp_active
== 41]),
            species_final_contribution = mean(mean_contribution[Nsp_active ==
10]))


# select only top 20 species based off species mean contribution at the
starting richness
top20_Xs_B14_Contributions <- top20_Xs_B14_Contributions %>%
  arrange(-species_starting_contribution) %>%
  group_by(Nsp_active) %>%
  slice(1:20)

rm(top_Xs_B14_Contributions)

B14_B13_Contributions$species <- gsub("_", " ", B14_B13_Contributions$species)

top_B14_B13_Contributions <- B14_B13_Contributions %>%
  group_by(species, Nsp_active) %>%
  summarise(mean_contribution = mean(species_contribution, na.rm = T))

top20_B14_B13_Contributions <- top_B14_B13_Contributions %>%
  group_by(species) %>%
  summarise(Nsp_active = Nsp_active,
            mean_contribution = mean_contribution,
            species_mean_contribution = mean(mean_contribution),
            species_starting_contribution = mean(mean_contribution[Nsp_active
== 27]),
            species_final_contribution = mean(mean_contribution[Nsp_active ==
11]))


# select only top 20 species based off species mean contribution at the
starting richness
top20_B14_B13_Contributions <- top20_B14_B13_Contributions %>%
  arrange(-species_starting_contribution) %>%
  group_by(Nsp_active) %>%
  slice(1:20)

rm(top_B14_B13_Contributions)

B17_B13_Contributions$species <- gsub("_", " ", B17_B13_Contributions$species)
```

```r
top_B17_B13_Contributions <- B17_B13_Contributions %>%
  group_by(species, Nsp_active) %>%
  summarise(mean_contribution = mean(species_contribution, na.rm = T))

top20_B17_B13_Contributions <- top_B17_B13_Contributions %>%
  group_by(species) %>%
  summarise(Nsp_active = Nsp_active,
            mean_contribution = mean_contribution,
            species_mean_contribution = mean(mean_contribution),
            species_starting_contribution = mean(mean_contribution[Nsp_active
== 52]),
            species_final_contribution = mean(mean_contribution[Nsp_active ==
11]))


# select only top 20 species based off species mean contribution at the
starting richness
top20_B17_B13_Contributions <- top20_B17_B13_Contributions %>%
  arrange(-species_starting_contribution) %>%
  group_by(Nsp_active) %>%
  slice(1:20)

rm(top_B17_B13_Contributions)

B17_B16_Taxon_graph <- ggplot(top20_B17_B16_Contributions, aes(x = Nsp_active,
y = reorder(species,species_starting_contribution), fill = mean_contribution))
+ geom_tile() +
  coord_cartesian(xlim = c(0,70)) +
  geom_vline(xintercept = 17, col = "red", linetype = "dashed", size =1.1) +
  geom_vline(xintercept = 52, col = "green", size = 1.1) +
  scale_fill_gradient(low = "azure", high = "#648FFF", limits = c(0,20)) +
  theme_classic() +
  theme(legend.position="right", axis.text.y = element_text(face = "italic")) +
  labs(x = "Species richness", y = "B17-B16",
       fill = "Contribution to BPc (%)")

B16_B15_Taxon_graph <- ggplot(top20_B16_B15_Contributions, aes(x = Nsp_active,
y = reorder(species,species_starting_contribution), fill = mean_contribution))
+ geom_tile() +
  coord_cartesian(xlim = c(0,70)) +
  geom_vline(xintercept = 15, col = "red", linetype = "dashed", size =1.1) +
  geom_vline(xintercept = 42, col = "green", size = 1.1) +
  scale_fill_gradient(low = "azure", high = "#648FFF", limits = c(0,20)) +
  theme_classic() +
  theme(legend.position="right", axis.text.y = element_text(face = "italic")) +
  labs(x = "Species richness", y = "B16-B15",
       fill = "Contribution to BPc (%)")

B15_Xs_Taxon_graph <- ggplot(top20_B15_Xs_Contributions, aes(x = Nsp_active, y
= reorder(species,species_starting_contribution), fill = mean_contribution)) +
geom_tile() +
  coord_cartesian(xlim = c(0,70)) +
  geom_vline(xintercept = 16, col = "red", linetype = "dashed", size =1.1) +
  geom_vline(xintercept = 40, col = "green", size = 1.1) +
  scale_fill_gradient(low = "azure", high = "#648FFF", limits = c(0,20)) +
  theme_classic() +
```

```
  theme(legend.position="right", axis.text.y = element_text(face = "italic")) +
  labs(x = "Species richness", y = "B15-Xs",
       fill = "Contribution to BPc (%)")

Xs_B14_Taxon_graph <- ggplot(top20_Xs_B14_Contributions, aes(x = Nsp_active, y
= reorder(species,species_starting_contribution), fill = mean_contribution)) +
geom_tile() +
  coord_cartesian(xlim = c(0,70)) +
  geom_vline(xintercept = 10, col = "red", linetype = "dashed", size =1.1) +
  geom_vline(xintercept = 41, col = "green", size = 1.1) +
  scale_fill_gradient(low = "azure", high = "#648FFF", limits = c(0,20)) +
  theme_classic() +
  theme(legend.position="right", axis.text.y = element_text(face = "italic")) +
  labs(x = "Species richness", y = "Xs-B14",
       fill = "Contribution to BPc (%)")

B14_B13_Taxon_graph <- ggplot(top20_B14_B13_Contributions, aes(x = Nsp_active,
y = reorder(species,species_starting_contribution), fill = mean_contribution))
+ geom_tile() +
  coord_cartesian(xlim = c(0,70)) +
  geom_vline(xintercept = 11, col = "red", linetype = "dashed", size =1.1) +
  geom_vline(xintercept = 27, col = "green", size = 1.1) +
  scale_fill_gradient(low = "azure", high = "#648FFF", limits = c(0,20)) +
  theme_classic() +
  theme(legend.position="right", axis.text.y = element_text(face = "italic")) +
  labs(x = "Species richness", y = "B14-B13",
       fill = "Contribution to BPc (%)")

B17_B13_Taxon_graph <- ggplot(top20_B17_B13_Contributions, aes(x = Nsp_active,
y = reorder(species,species_starting_contribution), fill = mean_contribution))
+ geom_tile() +
  coord_cartesian(xlim = c(0,70)) +
  geom_vline(xintercept = 11, col = "red", linetype = "dashed", size =1.1) +
  geom_vline(xintercept = 52, col = "green", size = 1.1) +
  scale_fill_gradient(low = "azure", high = "#648FFF", limits = c(0,20)) +
  theme_classic() +
  theme(legend.position="right", axis.text.y = element_text(face = "italic")) +
  labs(x = "Species richness", y = "B17-B13",
       fill = "Contribution to BPc (%)")

    Taxon_plots <- wrap_elements(grid::textGrob("B17-B16", vjust = 8, rot = 0,
gp = grid::gpar(fontsize = 24))) +  wrap_elements(grid::textGrob("B16-B15",
vjust = 8, rot = 0, gp = grid::gpar(fontsize = 24))) +
  wrap_elements(grid::textGrob("B15-Xs", vjust = 8, rot = 0, gp =
grid::gpar(fontsize = 24))) +
  wrap_elements(grid::textGrob("Xs-B14", vjust = 8, rot = 0, gp =
grid::gpar(fontsize = 24))) +
  wrap_elements(grid::textGrob("B14-B13", vjust = 8, rot = 0, gp =
grid::gpar(fontsize = 24))) +
    plot_spacer() +
  wrap_elements(grid::textGrob("B17-B13", vjust = 8, rot = 0, gp =
grid::gpar(fontsize = 24))) +
      B17_B16_Taxon_graph + theme(axis.title = element_blank()) +
  B16_B15_Taxon_graph + theme(axis.title = element_blank()) +
  B15_Xs_Taxon_graph + theme(axis.title = element_blank()) +
  Xs_B14_Taxon_graph + theme(axis.title = element_blank()) +
```

```
  B14_B13_Taxon_graph + theme(axis.title = element_blank()) +
      plot_spacer() +
  B17_B13_Taxon_graph  + theme(axis.title = element_blank()) +
    plot_layout(ncol = 7, guides = "collect", widths = c(1,1,1,1,1,0.5,1))

Taxon_plots & theme(axis.text.x = element_text(size = 24),
                       axis.text.y = element_text(size = 12),
                    legend.key.size = unit(1,"cm"),
                    legend.title = element_text(size = 20),
                    legend.text = element_text(size = 12))

ggsave(
  "Figure3.tiff",
  plot = last_plot(),
  device = tiff,
  scale = 2,
  width = 50,
  height = 12,
  units = c("cm"),
  dpi = 300
)

# Figure 4: Climate vulnerability ~ Scenario ####
plot1 <- B17_B16_BPc %>%
  filter(!is.na(Nsp_active)) %>%
  group_by(Simulation, Nsp, Nsp_active) %>%
  reframe(Alive_Vulnerability = Alive_Vulnerability,
          AllExtSpExtProb =
mean(c(ExtSpExtProb,CoExtSpExtProb,CoExtSp2ExtProb), na.rm=T)) %>%
  filter(!is.nan(AllExtSpExtProb)) %>%
  ggplot(.,aes(x=Nsp_active,y=log(Alive_Vulnerability)))+
  geom_point(colour="grey",alpha=0.1, size = 1)+
  geom_point(aes(y=log(AllExtSpExtProb)), alpha=0.05, col = "purple", size = 1)
+
  geom_smooth(size= 4,method = lm, se = TRUE, aes(y =log(AllExtSpExtProb), x=
Nsp_active), col = "darkorchid4") +
  geom_smooth(size= 4,method = lm, se = TRUE, aes(y =log(Alive_Vulnerability),
x=  Nsp_active), col = "grey42") +
  geom_vline(xintercept = 17, col = "red", linetype = "dashed", size = 0.8) +
  geom_vline(xintercept = 52, col = "green", size = 0.8) +
  geom_text(size = 12,x = 65, y = -6.1, label = "a") +
  scale_y_continuous(name = expression(low %<-% "Climate change pressure (log)"
%->% high), limits = c(-6.5,-4)) +
  scale_x_continuous(limits = c(0,70)) +
  theme_classic()+
  theme(legend.position="right") +
  labs(y = "Vulnerability",
       x = "Species Richness") +
  plot_layout(tag_level = 'new')

plot2 <- B17_B16_BPc %>%
  filter(!is.na(Nsp_active)) %>%
  group_by(Simulation, Nsp, Nsp_active) %>%
  reframe(Alive_Vulnerability = Alive_Vulnerability,
          Extinction_Vulnerability = ExtSpExtProb,
          CoExtinction_Vulnerability =
```

```
mean(c(CoExtSpExtProb,CoExtSp2ExtProb), na.rm=T)) %>%
  filter(!is.na(Extinction_Vulnerability)) %>%
  ggplot(., aes(x=Nsp_active,y=log(Alive_Vulnerability)))+
  geom_point(colour="grey",alpha=0.05, size = 1)+
  geom_point(aes(y=log(Extinction_Vulnerability)), alpha=0.05, colour = "blue",
size = 1) +
  geom_point(aes(y=log(CoExtinction_Vulnerability)), alpha=0.05, colour =
"yellow2", size = 1) +
  geom_smooth(size= 4,method = lm, se = TRUE, aes(y
=log(Extinction_Vulnerability), x=  Nsp_active), col = "blue") +
  geom_smooth(size= 4,method = lm, se = TRUE, aes(y =log(Alive_Vulnerability),
x=  Nsp_active), col = "grey42") +
  geom_smooth(size= 4,method = lm, se = TRUE, aes(y
=log(CoExtinction_Vulnerability), x=  Nsp_active), col = "yellow3") +
#   ggpubr::stat_cor(npcy = 0.95, npcx = 0.05,
#                    aes(Nsp_active, log(Extinction_Vulnerability),
#                        label = paste(..r.label.., ..rr.label.., ..p.label..,
"n = ", ..n.., sep = "~~~")),
#                    geom = "label_npc", size = 3, col = "blue", label.y.npc =
"top", label.x.npc = "left") +
#
#   ggpubr::stat_cor(npcy = 0.9, npcx = 0.05,
#                    aes(Nsp_active, log(CoExtinction_Vulnerability),
#                        label = paste(..r.label.., ..rr.label.., ..p.label..,
"n = ", ..n.., sep = "~~~")),
#                    geom = "label_npc", size = 3, col = "yellow3", label.y.npc
= "top", label.x.npc = "left") +
  geom_vline(xintercept = 17, col = "red", linetype = "dashed", size = 0.8) +
  geom_vline(xintercept = 52, col = "green", size = 0.8) +
    geom_text(size = 12,x = 65, y = -6.1, label = "g") +
  scale_y_continuous(name = expression(low %<-% "Climate change pressure (log)"
%->% high), limits = c(-6.5,-4)) +
  scale_x_continuous(limits = c(0,70)) +
  theme_classic()+
  theme(legend.position="right") +
  labs(x = "Species Richness") + plot_layout(tag_level = 'new')

plot3 <- B16_B15_BPc %>%
  filter(!is.na(Nsp_active)) %>%
  group_by(Simulation, Nsp, Nsp_active) %>%
  reframe(Alive_Vulnerability = Alive_Vulnerability,
          AllExtSpExtProb =
mean(c(ExtSpExtProb,CoExtSpExtProb,CoExtSp2ExtProb), na.rm=T)) %>%
  ggplot(.,aes(x=Nsp_active,y=log(Alive_Vulnerability)))+
  geom_point(colour="grey",alpha=0.05, size = 1)+
  geom_point(aes(y=log(AllExtSpExtProb)), alpha=0.05, col = "purple", size = 1)
+
  geom_smooth(size= 4,method = lm, se = TRUE, aes(y =log(AllExtSpExtProb), x=
Nsp_active), col = "darkorchid4") +
  geom_smooth(size= 4,method = lm, se = TRUE, aes(y =log(Alive_Vulnerability),
x=  Nsp_active), col = "grey42") +
#   ggpubr::stat_cor(npcy = 0.95, npcx = 0.05,
#                    aes(Nsp_active, log(AllExtSpExtProb),
#                        label = paste(..r.label.., ..rr.label.., ..p.label..,
"n = ", ..n.., sep = "~~~")),
#                    geom = "label_npc", size = 3, col = "purple", label.y.npc
```

```
= "top", label.x.npc = "left") +
#
#   ggpubr::stat_cor(npcy = 0.9, npcx = 0.05,
#                    aes(Nsp_active, log(Alive_Vulnerability),
#                        label = paste(..r.label.., ..rr.label.., ..p.label..,
"n = ", ..n.., sep = "~~~")),
#                    geom = "label_npc", size = 3, col = "grey42", label.y.npc
= "top", label.x.npc = "left") +
  geom_vline(xintercept = 15, col = "red", linetype = "dashed", size = 0.8) +
  geom_vline(xintercept = 42, col = "green", size = 0.8) +
    geom_text(size = 12,x = 65, y = -6.1, label = "b") +
  scale_y_continuous(name = expression(low %<-% "Climate change pressure (log)"
%->% high), limits = c(-6.5,-4)) +
  scale_x_continuous(limits = c(0,70)) +
  theme_classic()+
  theme(legend.position="right") +
  labs(y = "Vulnerability",
       x = "Species Richness") + plot_layout(tag_level = 'new')

plot4 <- B16_B15_BPc %>%
  filter(!is.na(Nsp_active)) %>%
  group_by(Simulation, Nsp, Nsp_active) %>%
  reframe(Alive_Vulnerability = Alive_Vulnerability,
            Extinction_Vulnerability = ExtSpExtProb,
            CoExtinction_Vulnerability =
mean(c(CoExtSpExtProb,CoExtSp2ExtProb), na.rm=T)) %>%
  ggplot(., aes(x=Nsp_active,y=log(Alive_Vulnerability)))+
  geom_point(colour="grey",alpha=0.05, size = 1)+
  geom_point(aes(y=log(Extinction_Vulnerability)), alpha=0.05, colour = "blue",
size = 1) +
  geom_point(aes(y=log(CoExtinction_Vulnerability)), alpha=0.05, colour =
"yellow2", size = 1) +
  geom_smooth(size= 4,method = lm, se = TRUE, aes(y
=log(Extinction_Vulnerability), x=  Nsp_active), col = "blue") +
  geom_smooth(size= 4,method = lm, se = TRUE, aes(y =log(Alive_Vulnerability),
x=  Nsp_active), col = "grey42") +
  geom_smooth(size= 4,method = lm, se = TRUE, aes(y
=log(CoExtinction_Vulnerability), x=  Nsp_active), col = "yellow3") +
#   ggpubr::stat_cor(npcy = 0.95, npcx = 0.05,
#                    aes(Nsp_active, log(Extinction_Vulnerability),
#                        label = paste(..r.label.., ..rr.label.., ..p.label..,
"n = ", ..n.., sep = "~~~")),
#                    geom = "label_npc", size = 3, col = "blue", label.y.npc =
"top", label.x.npc = "left") +
#
#   ggpubr::stat_cor(npcy = 0.9, npcx = 0.05,
#                    aes(Nsp_active, log(CoExtinction_Vulnerability),
#                        label = paste(..r.label.., ..rr.label.., ..p.label..,
"n = ", ..n.., sep = "~~~")),
#                    geom = "label_npc", size = 3, col = "yellow3", label.y.npc
= "top", label.x.npc = "left") +
  geom_vline(xintercept = 15, col = "red", linetype = "dashed", size = 0.8) +
  geom_vline(xintercept = 42, col = "green", size = 0.8) +
    geom_text(size = 12,x = 65, y = -6.1, label = "h") +
  scale_y_continuous(name = expression(low %<-% "Climate change pressure (log)"
%->% high), limits = c(-6.5,-4)) +
```

```
  scale_x_continuous(limits = c(0,70)) +
  theme_classic()+
  theme(legend.position="right") +
  labs(x = "Species Richness") + plot_layout(tag_level = 'new')

plot5 <- B15_Xs_BPc %>%
  filter(!is.na(Nsp_active)) %>%
  group_by(Simulation, Nsp, Nsp_active) %>%
  reframe(Alive_Vulnerability = Alive_Vulnerability,
            AllExtSpExtProb = mean(c(ExtSpExtProb,CoExtSpExtProb), na.rm=T))
%>%
  ggplot(.,aes(x=Nsp_active,y=log(Alive_Vulnerability)))+
  geom_point(colour="grey",alpha=0.05, size = 1)+
  geom_point(aes(y=log(AllExtSpExtProb)), alpha=0.05, col = "purple", size = 1)
+
  geom_smooth(size= 4,method = lm, se = TRUE, aes(y =log(AllExtSpExtProb), x=
Nsp_active), col = "darkorchid4") +
  geom_smooth(size= 4,method = lm, se = TRUE, aes(y =log(Alive_Vulnerability),
x=  Nsp_active), col = "grey42") +
#   ggpubr::stat_cor(npcy = 0.95, npcx = 0.05,
#                    aes(Nsp_active, log(AllExtSpExtProb),
#                        label = paste(..r.label.., ..rr.label.., ..p.label..,
"n = ", ..n.., sep = "~~~")),
#                    geom = "label_npc", size = 3, col = "purple", label.y.npc
= "top", label.x.npc = "left") +
#
#   ggpubr::stat_cor(npcy = 0.9, npcx = 0.05,
#                    aes(Nsp_active, log(Alive_Vulnerability),
#                        label = paste(..r.label.., ..rr.label.., ..p.label..,
"n = ", ..n.., sep = "~~~")),
#                    geom = "label_npc", size = 3, col = "grey42", label.y.npc
= "top", label.x.npc = "left") +
  geom_vline(xintercept = 16, col = "red", linetype = "dashed", size = 0.8) +
  geom_vline(xintercept = 40, col = "green", size = 0.8) +
    geom_text(size = 12,x = 65, y = -6.1, label = "c") +
  scale_y_continuous(name = expression(low %<-% "Climate change pressure (log)"
%->% high), limits = c(-6.5,-4)) +
  scale_x_continuous(limits = c(0,70)) +
  theme_classic()+
  theme(legend.position="right") +
  labs(y = "Vulnerability",
       x = "Species Richness") + plot_layout(tag_level = 'new')

plot6 <- B15_Xs_BPc %>%
  filter(!is.na(Nsp_active)) %>%
  ggplot(., aes(x=Nsp_active,y=log(Alive_Vulnerability)))+
  geom_point(colour="grey",alpha=0.05)+
  geom_point(aes(y=log(ExtSpExtProb)), alpha=0.05, colour = "blue", size = 1) +
  geom_point(aes(y=log(CoExtSpExtProb)), alpha=0.05, colour = "yellow2", size =
1) +
  geom_smooth(size= 4,method = lm, se = TRUE, aes(y =log(ExtSpExtProb), x=
Nsp_active), col = "blue") +
  geom_smooth(size= 4,method = lm, se = TRUE, aes(y =log(Alive_Vulnerability),
x=  Nsp_active), col = "grey40") +
  geom_smooth(size= 4,method = lm, se = TRUE, aes(y =log(CoExtSpExtProb), x=
Nsp_active), col = "yellow3") +
```

```
#   ggpubr::stat_cor(npcy = 0.95, npcx = 0.05,
#                    aes(Nsp_active, log(ExtSpExtProb),
#                        label = paste(..r.label.., ..rr.label.., ..p.label..,
"n = ", ..n.., sep = "~~~")),
#                    geom = "label_npc", size = 3, col = "blue", label.y.npc =
"top", label.x.npc = "left") +
#
#   ggpubr::stat_cor(npcy = 0.9, npcx = 0.05,
#                    aes(Nsp_active, log(CoExtSpExtProb),
#                        label = paste(..r.label.., ..rr.label.., ..p.label..,
"n = ", ..n.., sep = "~~~")),
#                    geom = "label_npc", size = 3, col = "yellow3", label.y.npc
= "top", label.x.npc = "left") +
  geom_vline(xintercept = 16, col = "red", linetype = "dashed", size = 0.8) +
  geom_vline(xintercept = 40, col = "green", size = 0.8) +
    geom_text(size = 12,x = 65, y = -6.1, label = "i") +
  scale_y_continuous(name = expression(low %<-% "Climate change pressure (log)"
%->% high), limits = c(-6.5,-4)) +
  scale_x_continuous(limits = c(0,70)) +
  theme_classic()+
  theme(legend.position="right") +
  labs(x = "Species Richness") + plot_layout(tag_level = 'new')


plot7 <- Xs_B14_BPc %>%
  filter(!is.na(Nsp_active)) %>%
  group_by(Simulation, Nsp, Nsp_active) %>%
  reframe(Alive_Vulnerability = Alive_Vulnerability,
            AllExtSpExtProb = mean(c(ExtSpExtProb,CoExtSpExtProb), na.rm=T))
%>%
  ggplot(.,aes(x=Nsp_active,y=log(Alive_Vulnerability)))+
  geom_point(colour="grey",alpha=0.05, size = 1)+
  geom_point(aes(y=log(AllExtSpExtProb)), alpha=0.05, col = "purple", size = 1)
+
  geom_smooth(size= 4,method = lm, se = TRUE, aes(y =log(AllExtSpExtProb), x=
Nsp_active), col = "darkorchid4") +
  geom_smooth(size= 4,method = lm, se = TRUE, aes(y =log(Alive_Vulnerability),
x=  Nsp_active), col = "grey42") +
#   ggpubr::stat_cor(npcy = 0.95, npcx = 0.05,
#                    aes(Nsp_active, log(AllExtSpExtProb),
#                        label = paste(..r.label.., ..rr.label.., ..p.label..,
"n = ", ..n.., sep = "~~~")),
#                    geom = "label_npc", size = 3, col = "purple", label.y.npc
= "top", label.x.npc = "left") +
#
#   ggpubr::stat_cor(npcy = 0.9, npcx = 0.05,
#                    aes(Nsp_active, log(Alive_Vulnerability),
#                        label = paste(..r.label.., ..rr.label.., ..p.label..,
"n = ", ..n.., sep = "~~~")),
#                    geom = "label_npc", size = 3, col = "grey42", label.y.npc
= "top", label.x.npc = "left") +
  geom_vline(xintercept = 10, col = "red", linetype = "dashed", size = 0.8) +
  geom_vline(xintercept = 41, col = "green", size = 0.8) +
  geom_text(size = 12,x = 65, y = -6.1, label = "d") +
  scale_y_continuous(name = expression(low %<-% "Climate change pressure (log)"
%->% high), limits = c(-6.5,-4)) +
```

```
  scale_x_continuous(limits = c(0,70)) +
  theme_classic()+
  theme(legend.position="right") +
  labs(y = "Vulnerability",
       x = "Species Richness") + plot_layout(tag_level = 'new')

plot8 <- Xs_B14_BPc %>%
  filter(!is.na(Nsp_active)) %>%
  ggplot(., aes(x=Nsp_active,y=log(Alive_Vulnerability)))+
  geom_point(colour="grey",alpha=0.05, size = 1)+
  geom_point(aes(y=log(ExtSpExtProb)), alpha=0.05, color = "blue", size = 1) +
  geom_point(aes(y=log(CoExtSpExtProb)), alpha=0.05, colour = "yellow2", size =
1) +
  geom_smooth(size= 4,method = lm, se = TRUE, aes(y =log(ExtSpExtProb), x=
Nsp_active), col = "blue") +
  geom_smooth(size= 4,method = lm, se = TRUE, aes(y =log(Alive_Vulnerability),
x=  Nsp_active), col = "grey40") +
  geom_smooth(size= 4,method = lm, se = TRUE, aes(y =log(CoExtSpExtProb), x=
Nsp_active), col = "yellow3") +
#   ggpubr::stat_cor(npcy = 0.95, npcx = 0.05,
#                    aes(Nsp_active, log(ExtSpExtProb),
#                        label = paste(..r.label.., ..rr.label.., ..p.label..,
"n = ", ..n.., sep = "~~~")),
#                    geom = "label_npc", size = 3, col = "blue", label.y.npc =
"top", label.x.npc = "left") +
#
#   ggpubr::stat_cor(npcy = 0.9, npcx = 0.05,
#                    aes(Nsp_active, log(CoExtSpExtProb),
#                        label = paste(..r.label.., ..rr.label.., ..p.label..,
"n = ", ..n.., sep = "~~~")),
#                    geom = "label_npc", size = 3, col = "yellow3", label.y.npc
= "top", label.x.npc = "left") +
  geom_vline(xintercept = 10, col = "red", linetype = "dashed", size = 0.8) +
  geom_vline(xintercept = 41, col = "green", size = 0.8) +
    geom_text(size = 12,x = 65, y = -6.1, label = "j") +
  scale_y_continuous(name = expression(low %<-% "Climate change pressure (log)"
%->% high), limits = c(-6.5,-4)) +
  scale_x_continuous(limits = c(0,70)) +
  theme_classic()+
  theme(legend.position="right") +
  labs(x = "Species Richness") + plot_layout(tag_level = 'new')

plot9 <- B14_B13_BPc %>%
  filter(!is.na(Nsp_active)) %>%
  group_by(Simulation, Nsp, Nsp_active) %>%
  reframe(Alive_Vulnerability = Alive_Vulnerability,
          AllExtSpExtProb = mean(c(ExtSpExtProb,CoExtSpExtProb), na.rm=T))
%>%
  ggplot(.,aes(x=Nsp_active,y=log(Alive_Vulnerability)))+
  geom_point(colour="grey",alpha=0.05, size = 1)+
  geom_point(aes(y=log(AllExtSpExtProb)), alpha=0.05, col = "purple", size = 1)
+
  geom_smooth(size= 4,method = lm, se = TRUE, aes(y =log(AllExtSpExtProb), x=
Nsp_active), col = "darkorchid4") +
  geom_smooth(size= 4,method = lm, se = TRUE, aes(y =log(Alive_Vulnerability),
x=  Nsp_active), col = "grey42") +
```

```
#   ggpubr::stat_cor(npcy = 0.95, npcx = 0.05,
#                    aes(Nsp_active, log(AllExtSpExtProb),
#                        label = paste(..r.label.., ..rr.label.., ..p.label..,
"n = ", ..n.., sep = "~~~")),
#                    geom = "label_npc", size = 3, col = "purple", label.y.npc
= "top", label.x.npc = "left") +
#
#   ggpubr::stat_cor(npcy = 0.9, npcx = 0.05,
#                    aes(Nsp_active, log(Alive_Vulnerability),
#                        label = paste(..r.label.., ..rr.label.., ..p.label..,
"n = ", ..n.., sep = "~~~")),
#                    geom = "label_npc", size = 3, col = "grey42", label.y.npc
= "top", label.x.npc = "left") +
  geom_vline(xintercept = 11, col = "red", linetype = "dashed", size = 0.8) +
  geom_vline(xintercept = 27, col = "green", size = 0.8) +
  geom_text(size = 12,x = 65, y = -6.1, label = "e") +
  scale_y_continuous(name = expression(low %<-% "Climate change pressure (log)"
%->% high), limits = c(-6.5,-4)) +
  scale_x_continuous(limits = c(0,70)) +
  theme_classic()+
  theme(legend.position="right") +
  labs(y = "Vulnerability",
       x = "Species Richness") + plot_layout(tag_level = 'new')

plot10 <-  B14_B13_BPc %>%
  filter(!is.na(Nsp_active)) %>%
  ggplot(., aes(x=Nsp_active,y=log(Alive_Vulnerability)))+
  geom_point(colour="grey",alpha=0.05, size = 1)+
  geom_point(aes(y=log(ExtSpExtProb)), alpha=0.05, color = "blue", size = 1) +
  geom_point(aes(y=log(CoExtSpExtProb)), alpha=0.05, colour = "yellow2", size =
1) +
  geom_smooth(size= 4,method = lm, se = TRUE, aes(y =log(ExtSpExtProb), x=
Nsp_active), col = "blue") +
  geom_smooth(size= 4,method = lm, se = TRUE, aes(y =log(Alive_Vulnerability),
x=  Nsp_active), col = "grey40") +
  geom_smooth(size= 4,method = lm, se = TRUE, aes(y =log(CoExtSpExtProb), x=
Nsp_active), col = "yellow3") +
#   ggpubr::stat_cor(npcy = 0.95, npcx = 0.05,
#                    aes(Nsp_active, log(ExtSpExtProb),
#                        label = paste(..r.label.., ..rr.label.., ..p.label..,
"n = ", ..n.., sep = "~~~")),
#                    geom = "label_npc", size = 3, col = "blue", label.y.npc =
"top", label.x.npc = "left") +
#
#   ggpubr::stat_cor(npcy = 0.9, npcx = 0.05,
#                    aes(Nsp_active, log(CoExtSpExtProb),
#                        label = paste(..r.label.., ..rr.label.., ..p.label..,
"n = ", ..n.., sep = "~~~")),
#                    geom = "label_npc", size = 3, col = "yellow3", label.y.npc
= "top", label.x.npc = "left") +
  geom_vline(xintercept = 11, col = "red", linetype = "dashed", size = 0.8) +
  geom_vline(xintercept = 27, col = "green", size = 0.8) +
    geom_text(size = 12,x = 65, y = -6.1, label = "k") +
  scale_y_continuous(name = expression(low %<-% "Climate change pressure (log)"
%->% high), limits = c(-6.5,-4)) +
  scale_x_continuous(limits = c(0,70)) +
```

```
  theme_classic()+
  theme(legend.position="right") +
  labs(x = "Species Richness") + plot_layout(tag_level = 'new')

plot11 <- B17_B13_BPc %>%
  filter(!is.na(Nsp_active)) %>%
  group_by(Simulation, Nsp, Nsp_active) %>%
  reframe(Alive_Vulnerability = Alive_Vulnerability,
          AllExtSpExtProb = mean(c(ExtSpExtProb,CoExtSpExtProb), na.rm=T))
%>%
  ggplot(.,aes(x=Nsp_active,y=log(Alive_Vulnerability)))+
  geom_point(colour="grey",alpha=0.05, size = 1)+
  geom_point(aes(y=log(AllExtSpExtProb)), alpha=0.05, col = "purple", size = 1)
+
  geom_smooth(size= 4,method = lm, se = TRUE, aes(y =log(AllExtSpExtProb), x=
Nsp_active), col = "darkorchid4") +
  geom_smooth(size= 4,method = lm, se = TRUE, aes(y =log(Alive_Vulnerability),
x=  Nsp_active), col = "grey42") +
#   ggpubr::stat_cor(npcy = 0.95, npcx = 0.05,
#                    aes(Nsp_active, log(AllExtSpExtProb),
#                        label = paste(..r.label.., ..rr.label.., ..p.label..,
"n = ", ..n.., sep = "~~~")),
#                    geom = "label_npc", size = 3, col = "purple", label.y.npc
= "top", label.x.npc = "left") +
#
#   ggpubr::stat_cor(npcy = 0.9, npcx = 0.05,
#                    aes(Nsp_active, log(Alive_Vulnerability),
#                        label = paste(..r.label.., ..rr.label.., ..p.label..,
"n = ", ..n.., sep = "~~~")),
#                    geom = "label_npc", size = 3, col = "grey42", label.y.npc
= "top", label.x.npc = "left") +
  geom_vline(xintercept = 11, col = "red", linetype = "dashed", size = 0.8) +
  geom_vline(xintercept = 52, col = "green", size = 0.8) +
  geom_text(size = 12,x = 65, y = -6.1, label = "f") +
  scale_y_continuous(name = expression(low %<-% "Climate change pressure (log)"
%->% high), limits = c(-6.5,-4)) +
  scale_x_continuous(limits = c(0,70)) +
  theme_classic()+
  theme(legend.position="right") +
  labs(y = "Vulnerability",
       x = "Species Richness") + plot_layout(tag_level = 'new')

plot12 <-  B17_B13_BPc %>%
  filter(!is.na(Nsp_active)) %>%
  ggplot(., aes(x=Nsp_active,y=log(Alive_Vulnerability)))+
  geom_point(colour="grey",alpha=0.05, size = 1)+
  geom_point(aes(y=log(ExtSpExtProb)), alpha=0.01, color = "blue", size = 1) +
  geom_point(aes(y=log(CoExtSpExtProb)), alpha =0.01, colour = "yellow2", size
= 1) +
  geom_smooth(size= 4,method = lm, se = TRUE, aes(y =log(ExtSpExtProb), x=
Nsp_active), col = "blue") +
  geom_smooth(size= 4,method = lm, se = TRUE, aes(y =log(Alive_Vulnerability),
x=  Nsp_active), col = "grey40") +
  geom_smooth(size= 4,method = lm, se = TRUE, aes(y =log(CoExtSpExtProb), x=
Nsp_active), col = "yellow3") +
#   ggpubr::stat_cor(npcy = 0.95, npcx = 0.05,
```

```
#                     aes(Nsp_active, log(ExtSpExtProb),
#                         label = paste(..r.label.., ..rr.label.., ..p.label..,
"n = ", ..n.., sep = "~~~")),
#                     geom = "label_npc", size = 3, col = "blue", label.y.npc =
"top", label.x.npc = "left") +
#
#   ggpubr::stat_cor(npcy = 0.9, npcx = 0.05,
#                     aes(Nsp_active, log(CoExtSpExtProb),
#                         label = paste(..r.label.., ..rr.label.., ..p.label..,
"n = ", ..n.., sep = "~~~")),
#                     geom = "label_npc", size = 3, col = "yellow3", label.y.npc
= "top", label.x.npc = "left") +
  geom_vline(xintercept = 11, col = "red", linetype = "dashed", size = 0.8) +
  geom_vline(xintercept = 52, col = "green", size = 0.8) +
  geom_text(size = 12,x = 65, y = -6.1, label = "L") +
  scale_y_continuous(name = expression(low %<-% "Climate change pressure (log)"
%->% high), limits = c(-6.5,-4)) +
  scale_x_continuous(limits = c(0,70)) +
  theme_classic()+
  theme(legend.position="right") +
  labs(x = "Species Richness")

Vulnerability_plots <- wrap_elements(grid::textGrob("B17-B16", vjust = 6, rot =
0, gp = grid::gpar(fontsize = 16))) +
  wrap_elements(grid::textGrob("B16-B15", vjust = 6, rot = 0, gp =
grid::gpar(fontsize = 16))) +
  wrap_elements(grid::textGrob("B15-Xs", vjust = 6, rot = 0, gp =
grid::gpar(fontsize = 16))) +
  wrap_elements(grid::textGrob("Xs-B14", vjust = 6, rot = 0, gp =
grid::gpar(fontsize = 16))) +
  wrap_elements(grid::textGrob("B14-B13", vjust = 6, rot = 0, gp =
grid::gpar(fontsize = 16))) +
  plot_spacer() +
  wrap_elements(grid::textGrob("B17-B13", vjust = 6, rot = 0, gp =
grid::gpar(fontsize = 16))) +
  (plot1 + theme(axis.title.x = element_blank(), axis.text.x = element_blank(),
axis.title.y = element_blank(), axis.text.y = element_text(size = 16))) +
  (plot3 + theme(axis.title.x = element_blank(), axis.text.x = element_blank(),
axis.title.y = element_blank(), axis.text.y = element_blank())) +
  (plot5 + theme(axis.title.x = element_blank(), axis.text.x = element_blank(),
axis.title.y = element_blank(),axis.text.y = element_blank())) +
  (plot7 + theme(axis.title.x = element_blank(), axis.text.x = element_blank(),
axis.title.y = element_blank(), axis.text.y = element_blank())) +
  (plot9 + theme(axis.title.x = element_blank(), axis.text.x = element_blank(),
axis.title.y = element_blank(), axis.text.y = element_blank())) +
  plot_spacer() +
  (plot11 + theme(axis.title.x = element_blank(), axis.text.x =
element_blank(), axis.title.y = element_blank(), axis.text.y =
element_text(size = 16))) +
  (plot2 + theme(axis.title.x = element_blank(), axis.title.y =
element_blank(), axis.text.x = element_text(size = 16),
                        axis.text.y = element_text(size = 16))) +
  (plot4 + theme(axis.title.x = element_blank(), axis.title.y =
element_blank(), axis.text.y = element_blank(), axis.text.x = element_text(size
= 16))) +
  (plot6 + theme(axis.title.x = element_blank(), axis.title.y =
```

```
element_blank(), axis.text.y = element_blank(), axis.text.x = element_text(size
= 16))) +
  (plot8 + theme(axis.title.x = element_blank(), axis.title.y =
element_blank(), axis.text.y = element_blank(), axis.text.x = element_text(size
= 16))) +
  (plot10 & theme(axis.title.x = element_blank(), axis.title.y =
element_blank(), axis.text.y = element_blank(), axis.text.x = element_text(size
= 16))) +
  plot_spacer() +
  (plot12 & theme(axis.title.x = element_blank(), axis.title.y =
element_blank(), axis.text.x = element_text(size = 16),
                       axis.text.y = element_text(size = 16))) +
  plot_layout(ncol = 7, nrow = 3, widths = c(1,1,1,1,1,0.3,1))

ggsave(
  "Figure4.tiff",
  plot = Vulnerability_plots,
  device = tiff,
  scale = 2,
  width = 24,
  height = 10,
  units = c("cm"),
  dpi = 300
)
```

References

Assis, J. et al**.** (2017) Bio-ORACLE v2.0: Extending marine data layers for bioclimatic modelling. *Global Ecology and Biogeography*, **27**, 277–284.

Bosch, S. & Fernandez, S. (2021) sdmpredictors: Species Distribution Modelling Predictor Datasets. R package version 0.2.10. Available at: *https://CRAN.R-project.org/package=sdmpredictors*

Degen, R. & Faulwetter, S. (2019) The Arctic Traits Database – a repository of Arctic benthic invertebrate traits. *Earth System Science Data*, **11**, 301–322.

Eriksen, E. et al. (2018) From single species surveys towards monitoring of the Barents Sea ecosystem. *Progress in Oceanography*, **166**, 4–14.

Fruchterman, T.M.J. & Reingold, E.M. (1991) Graph drawing by force-directed placement. *Software: Practice and Experience*, **21**, 1129–1164.

Gogina, M. et al. (2016) The Baltic Sea scale inventory of benthic faunal communities. *ICES Journal of Marine Science*, **73**, 1196–1213.

Loeng, H. (1991) Features of the physical oceanographic conditions of the Barents Sea. *Polar Research*, **10**, 5–18.

Morys, C. et al. (2017) Bioturbation in relation to the depth distribution of macrozoobenthos in the southwestern Baltic Sea. *Marine Ecology Progress Series*, **579**, 19–36.

Queirós, A.M. et al. (2013) A bioturbation classification of European marine infaunal invertebrates. *Ecology and Evolution*, **3**, 3958–3985.

Solan, M. et al. (2004) Extinction and ecosystem function in the marine benthos. *Science*, **306**, 1177–1180.

Solan, M. et al. (2020) Climate-driven benthic invertebrate activity and biogeochemical functioning across the Barents Sea polar front. *Philosophical Transactions of the Royal Society A: Mathematical, Physical and Engineering Sciences*, **378**, 20190365.

Tyberghein, L. et al. (2012) Bio-ORACLE: a global environmental dataset for marine species distribution modelling. *Global Ecology and Biogeography*, **21**, 272–281.

Vihtakari, M. et al. (2019) Barents Sea ocean-current arrows modified from Eriksen et al. (2018). Norwegian Polar Institute and Institute of Marine Research. Available at: *https://github.com/MikkoVihtakari/Barents-Sea-currents*

Williams, T.J. et al. (2024) Ocean warming and acidification adjust inter- and intra-specific variability in the functional trait expression of polar invertebrates. *Scientific Reports*, **14**, 14985.
